# Multi-disciplinary supportive end of life care in long-term care: an integrative approach to improving end of life

**DOI:** 10.1186/s12877-021-02271-1

**Published:** 2021-05-22

**Authors:** Patricia M. Harasym, Misha Afzaal, Sarah Brisbin, Aynharan Sinnarajah, Lorraine Venturato, Patrick Quail, Sharon Kaasalainen, Sharon Straus, Tamara Sussman, Navjot Virk, Jayna M. Holroyd-Leduc

**Affiliations:** 1 Cumming School of Medicine, University of Calgary, Calgary, Canada; 2 Faculty of Science, University of British Columbia, Vancouver, Canada; 3 Alberta Health Services, Calgary, Canada; 4 Faculty of Nursing, University of Calgary, Calgary, Canada; 5 School of Nursing, McMaster University, Hamilton, Canada; 6 Faculty of Medicine, University of Toronto, Toronto, Canada; 7 St Michael’s Hospital, Toronto, Canada; 8 School of Social Work, McGill University, Montreal, Canada; 9 Brenda Strafford Foundation, Calgary, Canada; 10 Foothills Medical Centre, 1403-29th Street NW, T2N 2T9 Calgary, Alberta Canada

**Keywords:** modified Delphi questionnaire, World Café Style workshop, 5-point supportive end of life care strategy for LTC

## Abstract

**Background:**

Optimal supportive end of life care for frail, older adults in long term care (LTC) homes involves symptom management, family participation, advance care plans, and organizational support. This 2-phase study aimed to combine multi-disciplinary opinions, build group consensus, and identify the top interventions needed to develop a supportive end of life care strategy for LTC.

**Methods:**

A consensus-building approach was undertaken in 2 Phases. The first phase deployed modified Delphi questionnaires to address and transform diverse opinions into group consensus. The second phase explored and prioritized the interventions needed to develop a supportive end of life care strategy for LTC. Development of the Delphi questionnaire was based on findings from published results of physician perspectives of barriers and facilitators to optimal supportive end of life care in LTC, a literature search of palliative care models in LTC, and published results of patient, family and nursing perspectives of supportive end of life care in long term care. The second phase involved World Café Style workshop discussions. A multi-disciplinary purposive sample of individuals inclusive of physicians; staff, administrators, residents, family members, and content experts in palliative care, and researchers in geriatrics and gerontology participated in round one of the modified Delphi questionnaire. A second purposive sample derived from round one participants completed the second round of the modified Delphi questionnaire. A third purposive sample (including participants from the Delphi panel) then convened to identify the top priorities needed to develop a supportive end-of-life care strategy for LTC.

**Results:**

19 participants rated 75 statements on a 9-point Likert scale during the first round of the modified Delphi questionnaire. 11 participants (participation rate 58 %) completed the second round of the modified Delphi questionnaire and reached consensus on the inclusion of 71candidate statements. 35 multidisciplinary participants discussed the 71 statements remaining and prioritized the top clinical practice, communication, and policy interventions needed to develop a supportive end of life strategy for LTC.

**Conclusions:**

Multi-disciplinary stakeholders identified and prioritized the top interventions needed to develop a 5-point supportive end of life care strategy for LTC.

**Supplementary Information:**

The online version contains supplementary material available at 10.1186/s12877-021-02271-1.

## Background

Supportive end of life care for frail older adults within long term care homes is an emergent practice in Canada and around the world [1–3]. Frail, older adults have complex medical and functional needs, and families, nurses, and care aides desire meaningful involvement and collaboration with physicians in end-of-life comfort care decision-making [4–8]. A search of the literature on palliative care models in LTC suggests that ideal palliative care includes family involvement, access to palliative care specialists, end of life care education and training, support for nurses and healthcare aides, and early identification of imminent end of life symptoms by front-line staff that then is proactively communicated to physicians [5, 9–11]. Research on the perspectives of LTC residents and families confirms that residents and families desire self-determined participation in end-of-life comfort care decision-making [8]. Families and residents also desire access to information about end of life care, and opportunities to share their feelings about a fellow resident’s death with other carers [8]. In Canada, healthcare aides, under nurse supervision, are trained individuals who provide as much as 80 % of direct physical and emotional care to long term care residents [12]. Nurses and healthcare aides aim to provide personalized attention and comfort care for the families of residents and for residents themselves near end of life [8].

Our research on physician involvement suggests that family and palliative care specialist physicians find end of life symptom assessment and control difficult, and open communication with families and relationships with nurses and healthcare aides to be important, but also associated with challenges [13]. Physician beliefs that families lack knowledge, have unrealistic expectations and experience grief emotions that cloud clear thinking could constrain open communication between them and families [13]. Physician perceptions of nursing staff as over-extended and insufficiently trained could impact trust relationships between physicians, nurses, and healthcare aides [13]. Though the studies discussed identify distinct stakeholder perspectives, none of them use complementary methodologies that would allow stakeholders to integrate diverse perspectives and reach consensus on the primary interventions needed to improve end of life care in long term care homes.

This project aimed to include and involve a diverse set of stakeholders with an interest and /or experience in end-of-life care in long term care to reach consensus and prioritize the interventions needed to develop a supportive end of life care strategy for LTC.

## Methods

We took a consensus-building, priority-setting approach in 2 phases. We first used 2 rounds of a modified Delphi questionnaire [14] to reach group consensus. A World-Café Style [15] workshop followed to prioritize the top clinical practice and policy interventions needed to develop a supportive end of life care strategy for LTC. A modified Delphi survey is an iterative group facilitation technique that transforms opinion into group consensus [14]. World Café Style facilitated conversations allow participants to prioritize statements, determine goals and actions, and reach consensus on interventions [16]. The Michie Behaviour System Framework, comprised of 3 categories: Motivations, Capabilities, and Opportunities [17] was used to help workshop participants identify the top clinical practice and policy change interventions needed, and to develop the final 5-point improvement strategy.

### Part 1: Modified Delphi surveys rounds one and two

We used several sources to develop a modified Delphi questionnaire: (1) the published results of physicians perspectives of barriers and facilitators to optimal supportive end of life care in LTC [13]; (2) the results of an unpublished systematic literature review of emergent models of palliative care in LTC, and (3) findings from published research on patient, family, and nursing perspectives of initiatives needed to strengthen a supportive palliative care in LTC [8]. Of the 703 citations reviewed for the systematic review, 25 articles were included and five interventions identified to improve end-of-life care in LTC (5 included modified Delphi statements): (1) engaging residents and families in end-of-life discussions, (2) timely identification and communication by LTC staff of residents whose condition has deteriorated, (3) providing LTC staff with education and training about end-of-life care, (4) increasing assess to spiritual care, and (5) providing access to palliative care consultants. PH selected statements common across the qualitative interviews conducted with physicians working in LTC (though unpublished then, a manuscript based on the physician interviews is now published) [13]. JHL identified statements from published research conducted by research team members TS and SK on patient, family, and nursing perspectives of palliative care in LTC [8]. JHL and SB selected statements that represented the best evidence of optimal palliative care practices for LTC found in the unpublished systematic review. All statements collected from all sources were presented, discussed and emergent themes were suggested, adapted, and developed iteratively through the collaboration of 6 members of the research team (JHL, PH, SB, LV, AS, PQ). The final list of 75 survey statements were organized by the team into 4 themes: (i) managing pain and other symptoms to optimize quality of life, (ii) managing end of life, (iii) topics related to families, and (iv) the context of providing supportive end of life care within LTC. The questionnaire was piloted with 4 members of the research team (JHL, SB, PH, AS) and incorporated feedback to create the final modified Delphi questionnaire Round One (Additional file 1).

A purposive sample of 65 multi-disciplinary stakeholders from across Canada were recruited for the modified Delphi. In addition to a sample of physicians interviewed for the published qualitative interview study [13], participants were recruited through partner organizations (The Brenda Strafford Foundation [18], The Brenda Strafford Centre on Aging [19], and Alberta Health Services Seniors Health Strategic Clinical Network [20]), and by word of mouth. In addition to physicians, we purposively sampled long-term care registered and practical nurses, healthcare aides, administrators, residents and their family members, content experts in geriatrics and gerontology, and knowledge translation researchers. Participant responses were confidential, not anonymous. Participant email addresses were collected and entered into the REDCap e-platform on a Participant List [21]. A “Participant Identifier” field linked the email addresses to the survey responses. Access to the association between the individual who took the survey, and the survey responses is restricted in the database and can only be accessed by authorized privileged users (system engineers, database admins). These privileged users act as “Honest Brokers” who provide information to investigators in such a manner that it would not be reasonably possible for the investigator or others to identify the corresponding patients-subjects directly or indirectly. REDCap holds the key to the code.

The questionnaire was distributed and data collected by email using the e-platform REDCap [21]. Round One was distributed, and data collected on-line through REDCap [21] between April 1 and April 22, 2019. The demographic information collected included role related to LTC, age, and primary place of work/residence. Round One modified Delphi participants received the preliminary findings of interviews conducted with physicians working in LTC, and findings from the unpublished systematic review. Participants rated each item on a 9-point Likert scale. Group results were reviewed, and median results calculated. Our established threshold for inclusion in Round Two of the modified Delphi was when the median score for a statement was greater than or equal to 7, and exclusion if less than 3. Participants rated all but 4 statements greater than or equal to 7. These 4 statements comprised Round Two of the modified Delphi questionnaires (Additional file 2) which was distributed to the same Round One multi-stakeholder purposive sample. Round Two participants received a personalized questionnaire with their prior ratings, the median results of the group, and narrative comments provided in Round One. Participants re-rated each item on a 9-point Likert Scale. Results were reviewed and median results calculated. Round Two was distributed and data collected between May 17 and May 31, 2019. At the end of Round Two of the modified Delphi survey, 71 statements remained. The 4 statements that remained inconclusive were excluded.

### Part 2: World Café Style Consensus Workshop

Once the 2-round Delphi process was complete, the list of 71 statements was taken to a 1-day World Café Style facilitated discussions consensus workshop held on June 10, 2019 in Calgary, Canada. A purposive sample of multi-disciplinary workshop participants were invited to participate. Participants were recruited from the cohort of physicians interviewed for the qualitative study, from all those invited to participate in the modified Delphi, and all members of the research team. Participants received the workshop agenda by email beforehand. The workshop was organized in two stages. First, research team members presented background information to the study (JHL), the systematic review methods and findings (SB), physician interview qualitative study methods and findings (PH), and published research on nursing, family, and resident perspectives of end of life in long term care (LV, SK). The World Café discussions followed.

At the start of the World Café Style discussions, the 35 participants organized themselves into four table groups of 8 or 9 persons per table. Material available to participants at each table to inform the discussions and prioritization of interventions included the COM-B (Capabilities, Opportunities, Motivations) System and Behaviour Change Wheel Framework [17], Worksheets for each topic based on the COM-B System, and the final modified Delphi report (Additional file 3). Individuals participated in the round-table discussions on one of the four topics, Managing End of Life, Managing Pain and Other Symptoms, Topics Related to Families, The Context of Providing End of Life Care, for 15 min before moving to a different table and topic. The workshop setting encouraged open, yet focused conversation. Table facilitators, attentive to the possibility of giving greater voice to more dominant individuals and groups [22] enabled the equitable participation of all participants in the round table discussions. Table facilitators recorded the main points of argumentation articulated in each round of discussion and shared the outcomes of prior rounds for further discussion with each new group as participants moved between tables and topics for further discussion and clarification. A note-taker assigned to each table noted the phrases and themes that arose within each table group as participants collaborated to generate and prioritize the top interventions needed in each category to improve supportive end of life care in LTC. Note-takers then presented the findings generated on each topic to the larger group for further discussion. A member of the research team (PH) then used the Michie COM-B System framework [17] to organize these discussion notes for the final report. A supportive end of life care strategy for LTC was developed based on the consensus workshop.

On completion of the study, all completed surveys, facilitator fieldnotes, and flipchart data were digitally scanned and transferred to a password protected, secure drive behind a firewall. Survey and data collection ceased organically through completion of two rounds of the Delphi Survey. Consensus workshop data collection ceased upon workshop completion. A non-judgmental stance [22] towards all perspectives was taken throughout the study. Modified Delphi questionnaire themes and statements emerged iteratively from the physician interviews, unpublished systematic literature review, and previously published work on nurses, residents and family perspectives on how to improve a supportive approach to end-of-life care in LTC Equitable facilitation techniques were deployed in the workshop discussions.

## Results

### Phase 1: Modified Delphi Survey

The purposive sample of 65 individuals included 28 content experts, 18 physicians, and a combination of 19 nurse administrators, nurses, healthcare aides, allied health professionals, LTC  residents, and family members (Table 1).

**Table 1:** Modified Delphi Participant Demographics

Modified Delphi Round	Round One	Round Two
Role (n)	Family Physician [4] Researcher/KT Expert [4] LTC Manager/Administrator [4] Family Member of LTC Resident [2] Nurse (RN, LPN) [2] Specialist Physician [1] Health Care Aide [1] LTC Resident [1]	Family Physician [3] Researcher/KT Expert [2] LTC Manager/Administrator [2] Family member of LTC Resident [2] Nurse (RN, LPN) [1] Specialist Physician [1]
**Age in years (n)**	31–39 [2] 40–49 [3] 50–59 [9] 60–69 [3] 70–79 [2]	31–39 [1] 50–59 [7] 60–69 [2] 70–79 [1]
Province (n)	Alberta [17] Ontario [1] Manitoba [1]	Alberta [9] Ontario [1] Manitoba [1]
Primary place of Work/Residence	Large Urban [16] Medium Urban [1] Small Urban [2]	Large Urban [10] Medium Urban [1]

Urban area: a population of at least 1000 and a density of 400 or more people per square kilometer. Large population: population of 100,00 or over. Medium population: a population of between 30,000 and 99,999. Small population of between 1000 and 29, 999; Rural: all part outside an urban area

Over 2 survey rounds, Modified Delphi participants prioritized 71 of 75 statements (Additional file 4) for inclusion in Phase 2 World Café Style consensus-building discussions (Table 2). There was some variability in the degree to which participants prioritized the 71 statements included. The 8 statements that received the highest ratings of 8.5 or 9 emphasize importance of family involvement in end-of-life care, of team communication, and of having and using a documented palliative care pathway to support both family involvement and team communication. The 4 statements on which participants could not reach consensus refer to staff involvement in end-of-life care. Participants could not reach agreement on whether staff have sufficient time to use pain assessment tools or if staff feel comfortable with end-of-life care. Neither could participants reach consensus on staff knowledge, experience, or education capacities needed to manage symptoms and provide supportive end of life care.

**Table 2:** Modified Delphi Rounds One and Two Samples, Process and Results

Modified Delphi Round	Round One	Round Two
Purposive Sample	Physicians; LTC Staff & Administrators: LTC residents & family members; Palliative Care, Geriatric/Gerontology Experts/Researchers	Physicians; LTC Staff & Administrators: LTC residents & family members; Palliative Care, Geriatric/Gerontology Experts/Researchers
Process	75 statements developed from physician interviews & systematic review/published literature Rated on a 9-point Likert scale Appropriateness ≥ 7	4 Remaining Round One Statements 9-point Likert Scale Appropriateness ≥ 7
Results	19 participants 3 provinces 71 statements scored ≥ 7 4 statements scored < 7 and > 3	11 participants 4 statements excluded 71 statements remained

### Phase 2: Multi-stakeholder World Café Style Consensus Meeting

 The World Café Style consensus meeting was attended by 35 stakeholders from across 3 provinces (Alberta, Manitoba, Ontario) representing family and palliative care physicians, nurses, healthcare aides, allied care providers, families, administrators and content experts in geriatrics/gerontology and knowledge translation. Given the equanimity orientation of World Café Style facilitation and discussion, we did not collect demographic information from participants. Participants used lists of statements on each of the 4 topics that remained after two modified Delphi survey rounds and the Michie Behaviour Change Wheel Framework material provided (Additional file 3) to determine the most important interventions needed in each category. Workshop flipcharts and note-taker notes reveal that through argumentation, conversation, and inclusion of all perspectives, participants combined, separated, tailored and/or adapted the statements to clarify and specify the exact nature of interventions most needed to improve supportive end of life care in LTC. For example, while participants agreed with the perspective that “medication choices should focus more on pain and symptom management and less on prevention”, participants specified that medication changes “should be accomplished in collaboration with the patient and/or family”. Using the Michie Behaviour Change Wheel as an interpretive framework, the workshop flip-chart data, notes taken, and results, (PH) then organized the results into 3 comprehensive lists of improvement interventions: (i) clinical practice knowledge and skills, (ii) communication motivations, (iii) policy and regulatory changes (Table 3).

**Table 3:** Top clinical practice interventions, communication factors, and policy/regulatory considerations required to improve end of life care in long term care facilities

**Clinical Practice Knowledge and Skills**
1. Develop, provide, and monitor compliance with an imminently dying pathway for use specifically at end of life 2. Create a communication checklist that physicians can adapt and use in conversations with LTC residents and their family for consistent messaging 3. Provide dementia education to staff that shows how dementia compromises health and limits life 4. Provide physicians/LTC staff with peer mentoring and access to the coaching and support of palliative care consultants 5. Provide palliative skills assessment and training specific to each health discipline 6. Provide palliative care knowledge, skills, and care standards though flexible pathways adaptable to diverse computer systems 7. Institute using the CHESS (Changes in Health, End-Stage Disease, Signs and Symptoms) quarterly rather than yearly to increase familiarity and better anticipate end of life 8. Include pain as a vital sign in routine assessments 9. Involve families in symptom management decisions
**Communication Factors**
**Conversation** 1. Use de-prescribing as a conversation opener to build relationships with families and enable future conversations about taking a palliative approach to end-of-life care 2. Perceive conversation with family as continuous and utilize current resources, such as the Serious Illness Conversation Guide [23] to unpack expectations 3. Have timely and open conversations between family and care providers **Language/Word Use** 4. Develop and use consistent end of life language in documents and face to face conversations when discussing care from admission to end of life 5. Clarify the meaning of “family” for each resident and the degree of family engagement desired 6. Remove unhelpful wording such as “authority” and the valuing of professional over lay expertise **Care Behaviours/Practices** 7. Use end of life order sets with caution as they can limit critical thinking and prevent validation of resident and family ideas about death and dying 8. Physicians to start palliative care approaches on admission and to routinely inform staff and family about changes in health status 9. Physicians regularly engage with families by writing a letter of expectations of families, attending family meetings, and dedicating time to meet with families throughout admission to end of life **Attitudes** 10. Incorporate team-building exercises into physician/LTC staff skills development training 11. Encourage sharing of thoughts, feelings, and ideas about life and death 12. Regard families as partners in care with experts 13. Create a social environment that makes it possible for healthcare aides to speak openly without fear 14. Perceive spiritual care as possibly, but not necessarily, connected to a system of beliefs or religion
**Policy/Regulatory Considerations**
**Physical Design Factors** 1. Legislate a government policy to mandate and regulate having access to private spaces within a LTC facility when death is imminent 2. Provide more recreational space and opportunities for socialization 3. Establish a separate space for spiritual contemplation 4. Provide a private space that families can use for talking and reflecting when approaching end of life **Social Design Factors** 5. Regulate increases in number of available staff at end of life, and assure continuity of staff for resident and family 6. Provide families with access to multi-disciplinary support 7. Create policies at the management, system, and government levels in order to mandate resident and family centered principles and processes 8. Enable, fund and enact resident and family centered care throughout the care trajectory within LTC

## Discussion

Clinical practice, communication and policy/regulatory changes are needed to improve supportive end of life care in long term care facilities. This study has implications for clinicians, administrators, and policymakers (regulations), researchers, and members of the public.

### Clinical Practice

Like prior research, this study identified the need for palliative care education, mentorship, and skills training for physicians, nurses and care aides in LTC [24]. Research has additionally shown that multi-disciplinary care for advanced illness in LTC has potential to improve clinical outcomes through improved team collaboration [25]. Consistent with prior research on family involvement in end-of-life care [26, 27], participants emphasized family involvement in care decision-making. Participants also highlighted family involvement in symptom assessment and in pain and symptom management. It should be noted, however, that there was high agreement among both modified Delphi survey cohorts and World Café participants about the importance of family involvement and that the study was exclusively comprised of Canadian “experts”. We can reasonably assume, therefore, that agreement reflects Canadian encultured perceptions of how the family group and the individual person relate. Canadian society in general values individual autonomy and the active involvement of older people in patient-centered care decision-making [26]. While the challenge in Canada is to more fully involve families in resident empowerment and engagement, families in other societies and cultures may tend to instead make decisions on behalf of their aging elders [28]. Future research could explore lived experiences of different cultures within the auspices of patient-focused care. Basic education and awareness of families, LTC staff, and physicians around frailty and end of life care issues also needs to be improved. A recent systematic review of palliative care interventions that address the needs of people living with dementia and in long term care acknowledges the inherent complexity of palliative care for this population [29]. However, the only dementia-related intervention mentioned by the World Cafe participants was the provision of education about how dementia compromises health and limits life. Given dementia impacts on both population and individual level experiences, it could be interesting to examine what long-term care staff currently understand about how dementia affects the health and life of older individuals and of the interventions staff perceive as helpful as dementia progresses.

Participants in this study used the modified Delphi statements as conversation starters that through face-to-face conversation, led to specific care improvement suggestions listed in Table 3. Modified Delphi statements taken at face value provide a partial view of needs; the more fulsome assessment produced, through co-present conversation, specific and practical knowledge of actual and specific needs, such as communication checklists, and palliative care training designed specifically for particular care providers.

This study additionally identified the need to develop, implement, and evaluate a unique pain assessment tool that would enable LTC nursing staff to assess, differentiate, and effectively communicate residents’ symptoms to physicians.

### Communication Factors

 Identified communication factors covered four areas: conversation, language use, care provider practices or behaviours, and attitudes. This finding supports prior research on how palliative care specialists hear and respond within face-to-face interactions to the perceptions, fears, anxieties and worries of dying patients and their families [30–33]. Specifically, this study confirmed prior research on the importance of family engagement in difficult conversations about end of life and advance care plans [34, 35]. The detailed nature of the World Café analysis and findings, however, contributes knowledge that would otherwise go unnoticed. For example, rather than regard difficult topics as inherently problematic and therefore to be avoided, participants defined conversations about difficult topics as the very means by which families and physicians develop trustworthy relationships. For example, while the use of the word “authority” in the modified Delphi statement naturalizes inequitable lay-expert relationships, the World Café discussions challenge the use of “authority” to promote a change in attitude. Unlike prior studies that emphasize and prescribe inclusive conversations, inclusivity was not only idealized, it was practiced within co-present World Café conversations [8]. Specifically, the voice of all those who work in the long-term care setting were present in the discussions, including family caregivers and healthcare aides who do not always have as many opportunities to inform care delivery strategies and polices. The World Café discussions were not audio-recorded. We do not know, therefore, the actual nature of healthcare aides and family’s involvement within the World Café discussions. This requires further investigation.

This study further highlighted the power of language to influence attitudes toward groups and individuals [8, 36–38]. Current understandings of the terms “vulnerable”, or “family” can either advance or limit quality care [38]. Our findings show how a term such as “family”, while noticed as limited by scope in multi-stakeholder conversations, can be used without question by physicians in the interviews on which the Delphi statements were derived. This has immediate, practical application for us as researchers and for our readers to reflect on how a word can shape and limit our perceptions of who caregivers are and what that means for them and the people they care for.

Participants validated earlier research suggesting a need for professionalized spiritual health resources for LTC residents and their families, but with a difference [39, 40]. World Café participants specified the need to define spiritual as possibly, but not necessarily connected to a system of beliefs or religion. Future research could investigate the details of how spiritual care services actually serve long term care residents and their families and the degree to which spiritual care practices and services meet the actual needs of residents, families, and staff.

### Policy and Regulatory Change

Long-term care facilities in Canada provide living accommodation for people who require on-site delivery of 24 h, 7 days a week supervised care. Long-term care facilities are governed by provincial and territorial legislation, and regulations vary within provinces in terms of type of care, level of care, and how it is offered [41]. The lack of national care and staffing standards and inconsistent policies and regulations between provinces could prevent widespread implementation of our results [42]. In fact, a recent policy briefing report produced in response to the higher number of COVID-19 deaths in Canadian long term care facilities compared to other countries identified the need for national or provincial reforms to standardize education, training, and staffing in LTC [43].

Like prior research, study participants identified the need to restructure the physical and social environments within LTC homes to better support optimal end of life care [44, 45]. Specifically, they identified a need for dedicated, comfortable and comforting physical environments for dying, death and grieving with dignity. Leadership was identified as responsible for instituting these structural changes within LTC. The role for policies and funds to regulate and provide extra staff was also mentioned as key to providing comfort care needs at end of life. This additionally suggests development of standards and fostering of attitudes and funds to support the provision of areas for private contemplation, socialization, and recreation.

### Researchers and Members of the Public

 Methodologically, the modified Delphi survey linked the published peer-reviewed research on the topic of palliative care in long term care, semi-structured interviews and physician perspectives, and all stakeholder groups. The 4 main areas for improvement within end-of-life care in long term care that emerged from physician interviews guided the involvement of other stakeholders in the modified Delphi surveys. The involvement of multiple stakeholders in the modified Delphi surveys generalized and validated physician perspectives. The World Café discussions then enabled members from within all stakeholder groups to discuss, argue, collaborate and translate abstract knowledge into practical applications. Neither interviews, modified Delphi questionnaires, nor World Café alone would have had the capacity to not only identify differences but to also integrate those differences into one view that is inclusive of differing perspectives.

This study has the potential to transform public perceptions of long-term care homes. It could invite the public to advocate for and support the provision and improvement of supportive end of life care within long term care homes from the moment an individual enters into a long-term care facility until death.

### Key Recommendations and Supportive End of Life Care Strategy for LTC

**Fig. 1:**
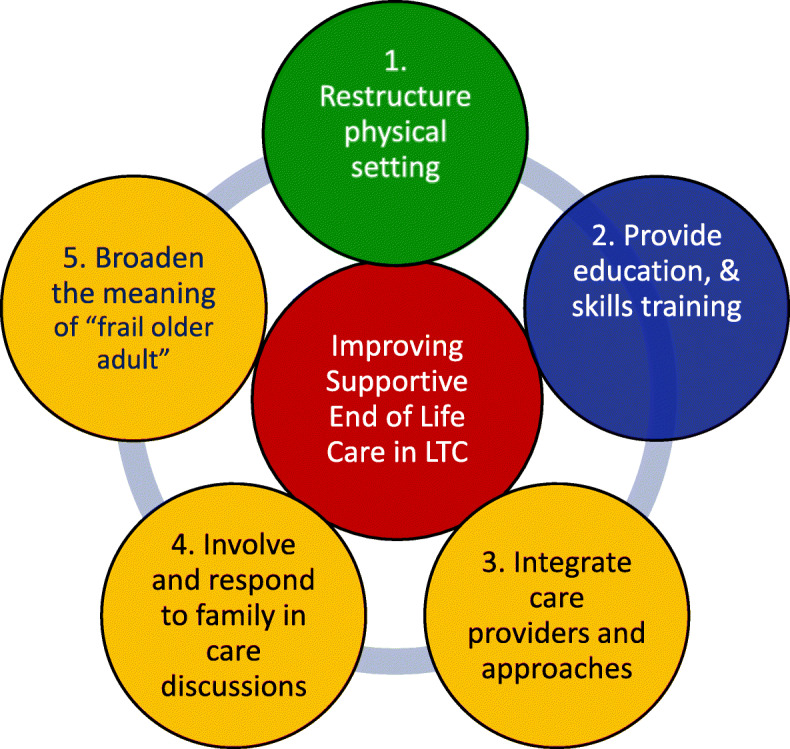
A supportive end of life care strategy for long term care based on the Michie Behaviour Change Wheel [17]

This study identified three key recommendations. The first is to establish knowledge and training expectations and resources for LTC staff and physicians, which focuses on a palliative approach, mentorship, communication and collaboration in LTC. The second is to build connections between all those involved in providing end of life care, including family members. The third is to create policies and provide funds needed to meet comfort care needs at end of life. Considering the identified priorities, we developed a 5-point strategy to providing supportive end of life care within LTC (Fig. 1).

The green circle indicates opportunities for change within the long-term care physical and social environment. The blue circle represents care capabilities for improvement through education and skills training. The yellow circles indicate changes in beliefs, attitudes and opinions to enhance communication and involvement of the entire multi-disciplinary end of life team.

Our results could be limited by the comparatively small number of residents and family member participants. The resident and family members who did participate, for example, were prone to answer “I don’t know” to the Delphi statements that made reference to medical practices. Family members were not involved in the study design. Our results are also limited by the disproportionate number of participants from Alberta and from those in large urban settings.

These limitations could affect the generalizability and transferability of our findings to other settings.

## Conclusions

Three lists of interventions required to optimize supportive end of life care in Canadian long-term care facilities include: clinical practice change, communication and culture change, and organizational policy change. Clinician educators can use our results to support the provision of palliative education and skills training, as well as mentorships. Administrators should explore opportunities to reconfigure LTC culture through communication changes, as well as the physical environment through facility design modifications. We have also shown the benefits of multi-method research that integrates qualitative interviews, quantitative surveys, and multi-stakeholder participatory methods in the development of evidence-based strategies to improve care within long-term care. Residents and family caregivers should also be engaged in research and change efforts targeted at optimizing end of life care provision within long-term care.

## Supplementary Information

**Additional file 1.**

**Additional file 2.**

**Additional file 3.**

**Additional file 4.**

## Data Availability

The data that support the findings of this study are available on request from the corresponding author. The data are not publicly available due to the fact it contains information that could compromise research participant privacy/consent.

## Abbreviations

LTC: Long term care
