# Supplementary material for: Multi-disciplinary supportive end of life care in long-term care: an integrative approach to improving end of life
Source: BMC Geriatr. 2021 May 22;21:326. doi: 10.1186/s12877-021-02271-1 (PMC8140573; doi:10.1186/s12877-021-02271-1)
Supplement: Supplementary file 1 — Additional file 1. [file 12877_2021_2271_MOESM1_ESM.pdf]

Delphi Questionnaire

Thank you for agreeing to participate in this Delphi process. Your participation is completely voluntary and you are free to withdraw from participating at any time.

As part of this Delphi process, you will be asked to rate how much you agree with a number of statements related to supportive end of life care within long term care (LTC). These statements were developed from the analysis of data obtained from interviews with 23 physicians practicing within LTC facilities in Alberta and/or from a systematic review of the literature related to end of life or palliative care models and practices within LTC. We will be using the results of this Delphi process to determine what aspects are most important to include in end of life pathways and/or processes within LTC. For the purpose of this Delphi survey, LTC staff is defined as non-physician health care providers (e.g. nurse; health care aides).

Although the statements in the survey came from the literature or the perspective of physicians, please respond to each statement from the unique perspective you bring. We are interested in obtaining a broad perspective on the survey statements. We will be considering the results of the survey in context to the larger body of work in this area, as we move forward with efforts to improve supportive end-of-life care within LTC.

The survey contains 4 demographic questions and 75 statements sorted into 4 categories. It takes about 10-15 minutes to complete, does not include the option to "Save and Return Later", and expires on April 14, 2019 at midnight.

Systematic Review and Qualitative Summary Links

If you would like a summary of the data used to develop the Delphi questionnaire (i.e. a summary of the literature review and a qualitative summary of the physician interviews), please click on PDF's below.

Systematic Review of the Literature Related to End-of-Life or Palliative Care Models or Practices in LTC

Please click on the link below to download and save to your computer.

[Attachment: "EOL Care Systematic Review Summary of Findings.pdf"]

Qualitative Summary of the Physician Interviews

Please click on the PDF below to download and save to your computer.

[Attachment: "Supportive Care in LTC Qualitative Summary 14 Feb 2019.pdf"]

Implied Consent Form

UNIVERSITY OF CALGARY

SURVEY INFORMATION/ IMPLIED CONSENT

TITLE: Improving Supportive End-of-Life Care in Long-Term Care

SPONSOR: Brenda Strafford Centre on Aging; Cumming School of Medicine

INVESTIGATORS: Dr. Jayna Holroyd-Leduc, Dr. Patricia Harasym, Dr. Lorraine Venturato, Dr. Aynharan Sinnarajah, Dr. Patrick Quail, (University of Calgary); Dr. Sharon Straus (University of Toronto); Dr. Sharon Kaasalainen (McMaster University); Dr. Tamara Sussman (McGill University)

Main contact: Dr. Jayna Holroyd-Leduc at 403-944-1771

This information sheet is only part of the process of informed consent. It should give you the basic idea of what the research is about and what your participation will involve. If you would like more detail about something mentioned here, or information not included here, please ask. Take the time to read this carefully and to understand any accompanying information.

BACKGROUND

Developing and implementing enhanced supportive end-of-life care services is currently a priority for long-term care (LTC) facilities across Canada, as frail older residents of LTC homes are highly likely to die in this setting (almost 40% of Canadians die in

Click on Next Page to go to Page 2

LTC). Supportive end-of-life care services aims to provide this patient population with relief from pain and other distressing symptoms, whilst maintaining quality of life. Multi-component supportive end-of-life care interventions generally include assessment and management of physical, psychological, and spiritual symptoms, along with advance care planning; these interventions also aim to support family members with coping and bereavement.

WHAT IS THE PURPOSE OF THE STUDY?

The purpose of this study is to determine what LTC residents, their families, LTC physicians, nurses, administrators and researchers prioritize in terms of supportive end-of-life care in LTC. You have been invited to participate in this survey to prioritize supportive end-of-life care practices. Ultimately, we aim to determine how to design and evaluate an optimized supportive end-of-life care program that engages all members of the LTC team.

WHAT WOULD I HAVE TO DO?

You are being asked to take part in a survey to prioritize supportive end-of-life care practices. This process will be conducted with ≥2 rounds via online surveys.
If you agree to participate in this study, you will be asked to participate in a 10-minute survey. The survey can be done online using RedCap.

If you would prefer, the research team can mail you a paper copy of the survey to complete and mail back. A member of the survey team will then enter your responses into the RedCap database.

Click on Next Page to go to Page 3

WHAT ARE THE RISKS?

Aside from giving of your time, we do not expect that there will be any risks or costs associated with taking part in this study.

WILL I BENEFIT IF I TAKE PART?

If you agree to participate in this study, there may or may not be a direct benefit to you. However, based on the results of this study, we hope that this study will help improve care for frail older adults living in LTC facilities.

DO I HAVE TO PARTICIPATE?

Being in this study is completely voluntary and you do not have to take part. Your decision whether to participate will not affect your current or future relationship with the researchers or anyone else at the University of Calgary. If you decide to take part in the study and then change your mind later, you are free to withdraw at any time. You can do this by exiting the survey. Submitting your completed questionnaire is an indication of your consent to participate in the study. You can withdraw your responses any time before you have submitted the questionnaire. Once you have submitted the questionnaire, your responses cannot be withdrawn because they are anonymous and therefore we will not be able to tell which one is yours.

WHAT ELSE DOES MY PARTICIPATION INVOLVE?

You will be invited to participate in the subsequent phase of this study involving an in-person consensus meeting in Calgary, Alberta to prioritize supportive end-of-life care practices for implementation in LTC facilities.

Please click Next Page to go to Page 4

WILL I BE PAID FOR PARTICIPATING, OR DO I HAVE TO PAY FOR ANYTHING?

Your participation is voluntary. You will not be paid for participating and your participation should not incur any expenses.

WILL MY RECORDS BE KEPT PRIVATE?

The survey data will be password protected and can only be accessed by the researchers. By providing your consent, you are agreeing to us collecting personal information about you for the purposes of this research study. Your information will only be used for the purposes outlined in this consent form, unless you consent otherwise. Study findings will be uploaded on to the CFN (project funder) Network-wide digital data management system (DDMS) once the project is complete and may be published in journal publications and conference presentations, but you will not be individually identifiable in the database or publications.

Your information will be stored securely, and your identity/information will be kept strictly confidential, except as required by law. The data will be kept in a password protected and secured disk owned by the researchers for a minimum of 5 years, then electronically erased and the disk will be reformatted.

If you elect to complete and mail a paper version of the survey, this paper survey will be stored in a locked cabinet within a locked office for a minimum of 5 years.

AGREEMENT TO PARTICIPATE

Click on Next Page to go to Page 5

Your decision to complete and return this survey will be interpreted as an indication of your agreement to participate. In no way does this waive your legal rights nor release the investigators or involved institutions from their legal and professional responsibilities. You are free to withdraw from the study at any time.

If you have further questions concerning matters related to this research, please contact:

Dr. Jayna Holroyd-Leduc (403) 944-1771

If you have any questions concerning your rights as a possible participant in this research, please contact the Chair of the Conjoint Health Research Ethics Board, Research Services, University of Calgary, 403-220-7990.

The University of Calgary Conjoint Health Research Ethics Board has approved this research study.

Please click on, Next Page to download a PDF of the Implied Consent and to Begin the Questionnaire

Click Submit (below) to begin the questionnaire

[Attachment: "Survey implied consent information sheet March 8, 2019.pdf"]

Demographic Information

Please answer each question.

1) What is your role in long-term care (LTC)?

- ◯ Family Physician
- ◯ Specialist Physician
- ◯ Nurse (Includes RN, LPN)
- ◯ Health Care Aide
- ◯ Healthcare provider (other than physician, RN, LPN, Health Care Aide)
- ◯ LTC Manager/Administrator
- ◯ Resident Living in LTC
- ◯ Family Member of Resident Living in LTC
- ◯ Researcher/Knowledge Translation Expert

2) What is your age?

- ◯ Equal to or under 30
- ◯ 31-39
- ◯ 40-49
- ◯ 50-59
- ◯ 60-69
- ◯ 70-79
- ◯ 80-89
- ◯ Equal to or over 90

3) In what province do you live?

- ◯ Alberta
- ◯ British Columbia
- ◯ Manitoba
- ◯ New Brunswick
- ◯ Newfoundland and Labrador
- ◯ Northwest Territories
- ◯ Nova Scotia
- ◯ Nunavit
- ◯ Ontario
- ◯ Prince Edward Island
- ◯ Quebec
- ◯ Saskatchewan
- ◯ Yukon

4) Based on population size, is your primary place of residence/work located in an urban or rural setting?

- ◯ Urban large size population (100,00 and over)
- ◯ Urban medium population (between 30,000 and 99,999)
- ◯ Urban small population (between 1000 and 29,999)
- ◯ Rural - territory located outside any size urban area

Click Submit (below) to begin the Delphi Round One Survey

Delphi Round One

Please indicate how strongly you agree or disagree with each statement using the 10-point scale provided

Managing Total Pain and Other Symptoms to Optimize Quality of Life. Part I (11 statements)

5) It is challenging to distinguish symptoms (i.e. confusion) related to pain from behaviours (i.e. confusion) related to dementia.

- ◯ Very Strongly Disagree
- ◯ Strongly Disagree
- ◯ Disagree
- ◯ Slightly Disagree
- ◯ Neither Agree nor Disagree
- ◯ Slightly Agree
- ◯ Agree
- ◯ Strongly Agree
- ◯ Very Strongly Agree
- ◯ I Don't Know

6) Pain assessment tools (E.G. PAINAD [Pain Assessment in Advanced Dementia]; PACSLAC [Pain Assessment Checklist]) are useful for measuring pain level in LTC residents with dementia.

- ◯ Very Strongly Disagree
- ◯ Strongly Disagree
- ◯ Disagree
- ◯ Slightly Disagree
- ◯ Neither Agree nor Disagree
- ◯ Slightly Agree
- ◯ Agree
- ◯ Strongly Agree
- ◯ Very Strongly Agree
- ◯ I Don't Know

7) Staff lack the time needed to use pain assessment tools.

- ◯ Very Strongly Disagree
- ◯ Strongly Disagree
- ◯ Disagree
- ◯ Slightly Disagree
- ◯ Neither Agree nor Disagree
- ◯ Slightly Agree
- ◯ Agree
- ◯ Strongly Agree
- ◯ Very Strongly Agree
- ◯ I Don't Know

8) Physicians find it challenging to appropriately manage pain if it is not documented in the patient's chart.

- ◯ Very Strongly Disagree
- ◯ Strongly Disagree
- ◯ Disagree
- ◯ Slightly Disagree
- ◯ Neither Agree nor Disagree
- ◯ Slightly Agree
- ◯ Agree
- ◯ Strongly Agree
- ◯ Very Strongly Agree
- ◯ I Don't Know

9) The adverse effects of opiates/narcotics, can make treating pain challenging in frail LTC residents.

- ◯ Very Strongly Disagree
- ◯ Strongly Disagree
- ◯ Disagree
- ◯ Slightly Disagree
- ◯ Neither Agree nor Disagree
- ◯ Slightly Agree
- ◯ Agree
- ◯ Strongly Agree
- ◯ Very Strongly Agree
- ◯ I Don't Know

10) LTC staff are often inexperienced giving narcotic medication, particularly when faced with "prn" (or as needed) medication orders for patients nearing end of life.

- ◯ Very Strongly Disagree
- ◯ Strongly Disagree
- ◯ Disagree
- ◯ Slightly Disagree
- ◯ Neither Agree nor Disagree
- ◯ Slightly Agree
- ◯ Agree
- ◯ Strongly Agree
- ◯ Very Strongly Agree
- ◯ I Don't Know

11) Access to technologies within LTC facilities (e.g.continuous IV pumps; oxygen masks; catheters to manage malignant effusions) can help facilitate supportive end of life care.

- ◯ Very Strongly Disagree
- ◯ Strongly Disagree
- ◯ Disagree
- ◯ Slightly Disagree
- ◯ Neither Agree nor Disagree
- ◯ Slightly Agree
- ◯ Agree
- ◯ Strongly Agree
- ◯ Very Strongly Agree
- ◯ I Don't Know

12) When LTC residents are nearing the end of their lives but not imminently dying, they should be given more opportunities to participate in physical activity.

- ◯ Very Strongly Disagree
- ◯ Strongly Disagree
- ◯ Disagree
- ◯ Slightly Disagree
- ◯ Neither Agree nor Disagree
- ◯ Slightly Agree
- ◯ Agree
- ◯ Strongly Agree
- ◯ Very Strongly Agree
- ◯ I Don't Know

13) Regular mobilization and maintaining independence in basic activities of daily living should be a priority in LTC.

- ◯ Very Strongly Disagree
- ◯ Strongly Disagree
- ◯ Disagree
- ◯ Slightly Disagree
- ◯ Neither Agree nor Disagree
- ◯ Slightly Agree
- ◯ Agree
- ◯ Strongly Agree
- ◯ Very Strongly Agree
- ◯ I Don't Know

14) Occupational therapy involvement is helpful for identifying and addressing functional changes throughout LTC admission to end of life.

- ◯ Very Strongly Disagree
- ◯ Strongly Disagree
- ◯ Disagree
- ◯ Slightly Disagree
- ◯ Neither Agree nor Disagree
- ◯ Slightly Agree
- ◯ Agree
- ◯ Strongly Agree
- ◯ Very Strongly Agree
- ◯ I Don't Know

15) Recreational activities such as gardening, caring for plants and animals, telling stories, and hearing children sing are helpful for managing pain and/or anxiety.

- ◯ Very Strongly Disagree
- ◯ Strongly Disagree
- ◯ Disagree
- ◯ Slightly Disagree
- ◯ Neither Agree nor Disagree
- ◯ Slightly Agree
- ◯ Agree
- ◯ Strongly Agree
- ◯ Very Strongly Agree
- ◯ I Don't Know

Please provide additional comments here (optional)

16) ______________________________

Managing Total Pain and Other Symptoms to Optimize Quality of Life Part II (13 statements)

17) Having the ability to access physicians in the emergency department for advice about acute medical issues, by phone on an as-needed basis, can help avoid transfer to hospital.

- ◯ Very Strongly Disagree
- ◯ Strongly Disagree
- ◯ Disagree
- ◯ Slightly Disagree
- ◯ Neither Agree nor Disagree
- ◯ Slightly Agree
- ◯ Agree
- ◯ Strongly Agree
- ◯ Very Strongly Agree
- ◯ I Don't Know

18) When caring for LTC residents, it is challenging to balance the management of several diseases (each of which often requires several medications) with the expectation in LTC to reduce the total number of medications.

- ◯ Very Strongly Disagree
- ◯ Strongly Disagree
- ◯ Disagree
- ◯ Slightly Disagree
- ◯ Neither Agree nor Disagree
- ◯ Slightly Agree
- ◯ Agree
- ◯ Strongly Agree
- ◯ Very Strongly Agree
- ◯ I Don't Know

19) Having access to specialist physician advice via phone can improve care.

- ◯ Very Strongly Disagree
- ◯ Strongly Disagree
- ◯ Disagree
- ◯ Slightly Disagree
- ◯ Neither Agree nor Disagree
- ◯ Slightly Agree
- ◯ Agree
- ◯ Strongly Agree
- ◯ Very Strongly Agree
- ◯ I Don't Know

20) Medication choices within LTC should focus more on pain and symptom management, and less on longer-term preventative care (e.g. lipid lowering; tight blood glucose control; blood pressure management).

- ◯ Very Strongly Disagree
- ◯ Strongly Disagree
- ◯ Disagree
- ◯ Slightly Disagree
- ◯ Neither Agree nor Disagree
- ◯ Slightly Agree
- ◯ Agree
- ◯ Strongly Agree
- ◯ Very Strongly Agree
- ◯ I Don't Know

21) Considering the short and long-term impact of medication on functional ability helps LTC physicians choose the appropriate types and numbers of medications.

- ◯ Very Strongly Disagree
- ◯ Strongly Disagree
- ◯ Disagree
- ◯ Slightly Disagree
- ◯ Neither Agree nor Disagree
- ◯ Slightly Agree
- ◯ Agree
- ◯ Strongly Agree
- ◯ Very Strongly Agree
- ◯ I Don't Know

22) Family can be engaged in helping to deliver non-medication (i.e. behavioural) interventions for residents in LTC

◯ Very Strongly Disagree
◯ Strongly Disagree
◯ Disagree
◯ Slightly Disagree
◯ Neither Agree nor Disagree
◯ Slightly Agree
◯ Agree
◯ Strongly Agree
◯ Very Strongly Agree
◯ I Don't Know

23) Having end of life orders sets can help guide supportive care decision-making once the patient is identified as needing palliative care.

◯ Very Strongly Disagree
◯ Strongly Disagree
◯ Disagree
◯ Slightly Disagree
◯ Neither Agree nor Disagree
◯ Slightly Agree
◯ Agree
◯ Strongly Agree
◯ Very Strongly Agree
◯ I Don't Know

24) Rather than (or in addition to) treating their symptoms with medication, providing space for residents to move and express themselves can help manage stress and anxiety.

◯ Very Strongly Disagree
◯ Strongly Disagree
◯ Disagree
◯ Slightly Disagree
◯ Neither Agree nor Disagree
◯ Slightly Agree
◯ Agree
◯ Strongly Agree
◯ Very Strongly Agree
◯ I Don't Know

25) The CAM (Confusion Assessment Method) is useful for detecting delirium in LTC residents with dementia.

◯ Very Strongly Disagree
◯ Strongly Disagree
◯ Disagree
◯ Slightly Disagree
◯ Neither Agree nor Disagree
◯ Slightly Agree
◯ Agree
◯ Strongly Agree
◯ Very Strongly Agree
◯ I Don't Know

26) Among persons with dementia, it is important to identify the underlying factors associated with a patient's responsive behaviors (e.g. aggression, calling out).

◯ Very Strongly Disagree
◯ Strongly Disagree
◯ Disagree
◯ Slightly Disagree
◯ Neither Agree nor Disagree
◯ Slightly Agree
◯ Agree
◯ Strongly Agree
◯ Very Strongly Agree
◯ I Don't Know

27) Among persons with dementia, it is important to include the family when trying to understand a patient's responsive behaviours.

- ◯ Very Strongly Disagree
- ◯ Strongly Disagree
- ◯ Disagree
- ◯ Slightly Disagree
- ◯ Neither Agree nor Disagree
- ◯ Slightly Agree
- ◯ Agree
- ◯ Strongly Agree
- ◯ Very Strongly Agree
- ◯ I Don't Know

28) Inconsistencies in how different LTC facilities chart symptoms can make it challenging for physicians and other healthcare providers that work within multiple different LTC facilities to care for residents.

- ◯ Very Strongly Disagree
- ◯ Strongly Disagree
- ◯ Disagree
- ◯ Slightly Disagree
- ◯ Neither Agree nor Disagree
- ◯ Slightly Agree
- ◯ Agree
- ◯ Strongly Agree
- ◯ Very Strongly Agree
- ◯ I Don't Know

29) Inconsistencies in how different LTC facilities chart symptoms can make it challenging for physicians and other healthcare providers that work within multiple different LTC facilities to communicate with other team members.

- ◯ Very Strongly Disagree
- ◯ Strongly Disagree
- ◯ Disagree
- ◯ Slightly Disagree
- ◯ Neither Agree nor Disagree
- ◯ Slightly Agree
- ◯ Agree
- ◯ Strongly Agree
- ◯ Very Strongly Agree
- ◯ I Don't Know

Please provide additional comments here (optional)

30) ______________________________

Managing End of Life (11 statements)

31) It can be challenging to determine where a frail resident is on the life-expectancy trajectory (or curve), making it hard to know when to transition from interventional medical care to palliative care.

- ◯ Very Strongly Disagree
- ◯ Strongly Disagree
- ◯ Disagree
- ◯ Slightly Disagree
- ◯ Neither Agree nor Disagree
- ◯ Slightly Agree
- ◯ Agree
- ◯ Strongly Agree
- ◯ Very Strongly Agree
- ◯ I Don't Know

32) Having a standardized frailty assessment tool that is used on a routine basis can help judge a patient's health status from LTC admission up until end of life.

- ◯ Very Strongly Disagree
- ◯ Strongly Disagree
- ◯ Disagree
- ◯ Slightly Disagree
- ◯ Neither Agree nor Disagree
- ◯ Slightly Agree
- ◯ Agree
- ◯ Strongly Agree
- ◯ Very Strongly Agree
- ◯ I Don't Know

33) The CHESS (Changes in Health, End-Stage Disease, Signs and Symptoms) score can be generated from data already collected within LTC and thus could be helpful in determining when to initiate end of life care if made available for each LTC resident.

- ◯ Very Strongly Disagree
- ◯ Strongly Disagree
- ◯ Disagree
- ◯ Slightly Disagree
- ◯ Neither Agree nor Disagree
- ◯ Slightly Agree
- ◯ Agree
- ◯ Strongly Agree
- ◯ Very Strongly Agree
- ◯ I Don't Know

34) The care needs of residents increase as they reach the end of their life, yet overall staffing levels remain the same.

- ◯ Very Strongly Disagree
- ◯ Strongly Disagree
- ◯ Disagree
- ◯ Slightly Disagree
- ◯ Neither Agree nor Disagree
- ◯ Slightly Agree
- ◯ Agree
- ◯ Strongly Agree
- ◯ Very Strongly Agree
- ◯ I Don't Know

35) Timely identification by LTC staff of residents whose condition has deteriorated and/or who appear at high risk of imminent death, and communicating this to the attending LTC physician can improve outcomes at end of life.

- ◯ Very Strongly Disagree
- ◯ Strongly Disagree
- ◯ Disagree
- ◯ Slightly Disagree
- ◯ Neither Agree nor Disagree
- ◯ Slightly Agree
- ◯ Agree
- ◯ Strongly Agree
- ◯ Very Strongly Agree
- ◯ I Don't Know

36) It can be difficult to access multidisciplinary support in my community (e.g. psychologists, SW, chaplaincy, chronic pain service).

◯ Very Strongly Disagree
◯ Strongly Disagree
◯ Disagree
◯ Slightly Disagree
◯ Neither Agree nor Disagree
◯ Slightly Agree
◯ Agree
◯ Strongly Agree
◯ Very Strongly Agree
◯ I Don't Know

37) Engaging residents and families in discussions about goals of care, treatment preferences and palliative care needs can improve end of life care outcomes.

◯ Very Strongly Disagree
◯ Strongly Disagree
◯ Disagree
◯ Slightly Disagree
◯ Neither Agree nor Disagree
◯ Slightly Agree
◯ Agree
◯ Strongly Agree
◯ Very Strongly Agree
◯ I Don't Know

38) A collaborative approach to care improves care by improving communication between team members.

◯ Very Strongly Disagree
◯ Strongly Disagree
◯ Disagree
◯ Slightly Disagree
◯ Neither Agree nor Disagree
◯ Slightly Agree
◯ Agree
◯ Strongly Agree
◯ Very Strongly Agree
◯ I Don't Know

39) A documented palliative care pathway helps to identify changes in patient status, manage family expectations, set staff expectations, and organize the provision of care.

◯ Very Strongly Disagree
◯ Strongly Disagree
◯ Disagree
◯ Slightly Disagree
◯ Neither Agree nor Disagree
◯ Slightly Agree
◯ Agree
◯ Strongly Agree
◯ Very Strongly Agree
◯ I Don't Know

40) It is important to determine and include family expectations in care decision making, and not rely exclusively on standardized care management protocols.

◯ Very Strongly Disagree
◯ Strongly Disagree
◯ Disagree
◯ Slightly Disagree
◯ Neither Agree nor Disagree
◯ Slightly Agree
◯ Agree
◯ Strongly Agree
◯ Very Strongly Agree
◯ I Don't Know

41) It is important to have private space within the LTC facility for the patient and family at or near end of life.

- ◯ Very Strongly Disagree
- ◯ Strongly Disagree
- ◯ Disagree
- ◯ Slightly Disagree
- ◯ Neither Agree nor Disagree
- ◯ Slightly Agree
- ◯ Agree
- ◯ Strongly Agree
- ◯ Very Strongly Agree
- ◯ I Don't Know

Please provide additional comments here (optional)

42) ________________________________

The Context of Providing Supportive End of Life Care within LTC Part I (12 Statements)

43) There is a lack of mental health care supports (e.g. Psychiatrist, Psychologist, SW) within LTC for residents with mental health conditions other than dementia.

- ◯ Very Strongly Disagree
- ◯ Strongly Disagree
- ◯ Disagree
- ◯ Slightly Disagree
- ◯ Neither Agree nor Disagree
- ◯ Slightly Agree
- ◯ Agree
- ◯ Strongly Agree
- ◯ Very Strongly Agree
- ◯ I Don't Know

44) Spiritual care providers working in LTC should have an understanding of frailty.

- ◯ Very Strongly Disagree
- ◯ Strongly Disagree
- ◯ Disagree
- ◯ Slightly Disagree
- ◯ Neither Agree nor Disagree
- ◯ Slightly Agree
- ◯ Agree
- ◯ Strongly Agree
- ◯ Very Strongly Agree
- ◯ I Don't Know

45) Increased access to spiritual care services can improve end of life care outcomes.

- ◯ Very Strongly Disagree
- ◯ Strongly Disagree
- ◯ Disagree
- ◯ Slightly Disagree
- ◯ Neither Agree nor Disagree
- ◯ Slightly Agree
- ◯ Agree
- ◯ Strongly Agree
- ◯ Very Strongly Agree
- ◯ I Don't Know

46) LTC residents frequently end up being transferred to acute care facilities as the result of inadequate staff to patient ratios needed to manage the increasing care needs of those nearing the end of life.

- ◯ Very Strongly Disagree
- ◯ Strongly Disagree
- ◯ Disagree
- ◯ Slightly Disagree
- ◯ Neither Agree nor Disagree
- ◯ Slightly Agree
- ◯ Agree
- ◯ Strongly Agree
- ◯ Very Strongly Agree
- ◯ I Don't Know

47) LTC staff are uncomfortable with providing supportive end of life care.

- ◯ Very Strongly Disagree
- ◯ Strongly Disagree
- ◯ Disagree
- ◯ Slightly Disagree
- ◯ Neither Agree nor Disagree
- ◯ Slightly Agree
- ◯ Agree
- ◯ Strongly Agree
- ◯ Very Strongly Agree
- ◯ I Don't Know

48) LTC staff are unknowledgeable and/or inexperienced in supportive end of life care.

◯ Very Strongly Disagree
◯ Strongly Disagree
◯ Disagree
◯ Slightly Disagree
◯ Neither Agree nor Disagree
◯ Slightly Agree
◯ Agree
◯ Strongly Agree
◯ Very Strongly Agree
◯ I Don't Know

49) Providing LTC staff with education and training about supportive end of life care improves end of life care practices.

◯ Very Strongly Disagree
◯ Strongly Disagree
◯ Disagree
◯ Slightly Disagree
◯ Neither Agree nor Disagree
◯ Slightly Agree
◯ Agree
◯ Strongly Agree
◯ Very Strongly Agree
◯ I Don't Know

50) Physicians can support LTC staff by encouraging them to attend palliative care education sessions on-line or in person, and advocating to management for the time and money LTC staff need in order to participate.

◯ Very Strongly Disagree
◯ Strongly Disagree
◯ Disagree
◯ Slightly Disagree
◯ Neither Agree nor Disagree
◯ Slightly Agree
◯ Agree
◯ Strongly Agree
◯ Very Strongly Agree
◯ I Don't Know

51) The best education for staff initially resistant to providing supportive end of life care in LTC is the practical knowledge they gain from managing symptoms in difficult situations.

◯ Very Strongly Disagree
◯ Strongly Disagree
◯ Disagree
◯ Slightly Disagree
◯ Neither Agree nor Disagree
◯ Slightly Agree
◯ Agree
◯ Strongly Agree
◯ Very Strongly Agree
◯ I Don't Know

52) Access to palliative care consultants improves the provision of end of life care in LTC.

◯ Very Strongly Disagree
◯ Strongly Disagree
◯ Disagree
◯ Slightly Disagree
◯ Neither Agree nor Disagree
◯ Slightly Agree
◯ Agree
◯ Strongly Agree
◯ Very Strongly Agree
◯ I Don't Know

53) By working with palliative care consultants, LTC physicians learn to utilize palliative (supportive) care principles and practices to treat symptoms and care for residents from admission to end-of-life.

- ◯ Very Strongly Disagree
- ◯ Strongly Disagree
- ◯ Disagree
- ◯ Slightly Disagree
- ◯ Neither Agree nor Disagree
- ◯ Slightly Agree
- ◯ Agree
- ◯ Strongly Agree
- ◯ Very Strongly Agree
- ◯ I Don't Know

54) It can be difficult to understand and manage the diverse ethnic values and attitudes of LTC staff toward death.

- ◯ Very Strongly Disagree
- ◯ Strongly Disagree
- ◯ Disagree
- ◯ Slightly Disagree
- ◯ Neither Agree nor Disagree
- ◯ Slightly Agree
- ◯ Agree
- ◯ Strongly Agree
- ◯ Very Strongly Agree
- ◯ I Don't Know

Please provide additional comments here (optional)

55) ______________________________

The Context of Providing Supportive End of Life Care within LTC Part II (11 Statements)

56) When they exist within a LTC facility, end of life protocols/pathways are followed inconsistently.

- ◯ Very Strongly Disagree
- ◯ Strongly Disagree
- ◯ Disagree
- ◯ Slightly Disagree
- ◯ Neither Agree nor Disagree
- ◯ Slightly Agree
- ◯ Agree
- ◯ Strongly Agree
- ◯ Very Strongly Agree
- ◯ I Don't Know

57) There are gaps in physician training and education in terms of the application of supportive end of life care practices within the LTC setting.

- ◯ Very Strongly Disagree
- ◯ Strongly Disagree
- ◯ Disagree
- ◯ Slightly Disagree
- ◯ Neither Agree nor Disagree
- ◯ Slightly Agree
- ◯ Agree
- ◯ Strongly Agree
- ◯ Very Strongly Agree
- ◯ I Don't Know

58) There are gaps in physician training and education in terms of the emotional aspects of delivering supportive end of life care in LTC.

- ◯ Very Strongly Disagree
- ◯ Strongly Disagree
- ◯ Disagree
- ◯ Slightly Disagree
- ◯ Neither Agree nor Disagree
- ◯ Slightly Agree
- ◯ Agree
- ◯ Strongly Agree
- ◯ Very Strongly Agree
- ◯ I Don't Know

59) LTC physicians often do not consult palliative care early enough in a patient's end of life care path.

- ◯ Very Strongly Disagree
- ◯ Strongly Disagree
- ◯ Disagree
- ◯ Slightly Disagree
- ◯ Neither Agree nor Disagree
- ◯ Slightly Agree
- ◯ Agree
- ◯ Strongly Agree
- ◯ Very Strongly Agree
- ◯ I Don't Know

60) LTC physicians' ability to consult with colleagues external to the LTC facility is a facilitator to providing care (e.g. physician colleagues, EMS, Nurse Practitioners, Psychiatrist Services, Geriatric Mental Health Services, Occupational Therapist, Physiotherapist, and Dental services).

- ◯ Very Strongly Disagree
- ◯ Strongly Disagree
- ◯ Disagree
- ◯ Slightly Disagree
- ◯ Neither Agree nor Disagree
- ◯ Slightly Agree
- ◯ Agree
- ◯ Strongly Agree
- ◯ Very Strongly Agree
- ◯ I Don't Know

61) Having access to staff and colleagues trained in the application of supportive end of life care helps the LTC physician support the patient and family.

- ◯ Very Strongly Disagree
- ◯ Strongly Disagree
- ◯ Disagree
- ◯ Slightly Disagree
- ◯ Neither Agree nor Disagree
- ◯ Slightly Agree
- ◯ Agree
- ◯ Strongly Agree
- ◯ Very Strongly Agree
- ◯ I Don't Know

62) Nurse practitioners can help relieve the supportive end of life care burden in LTC.

- ◯ Very Strongly Disagree
- ◯ Strongly Disagree
- ◯ Disagree
- ◯ Slightly Disagree
- ◯ Neither Agree nor Disagree
- ◯ Slightly Agree
- ◯ Agree
- ◯ Strongly Agree
- ◯ Very Strongly Agree
- ◯ I Don't Know

63) Changes in funding models for primary care physicians to work in LTC can improve physician involvement in LTC.

- ◯ Very Strongly Disagree
- ◯ Strongly Disagree
- ◯ Disagree
- ◯ Slightly Disagree
- ◯ Neither Agree nor Disagree
- ◯ Slightly Agree
- ◯ Agree
- ◯ Strongly Agree
- ◯ Very Strongly Agree
- ◯ I Don't Know

64) Public knowledge and negative perceptions about the quality of care provided within LTC influences a person's decision to attain a higher level of care at end of life (e.g. transfer to hospital).

- ◯ Very Strongly Disagree
- ◯ Strongly Disagree
- ◯ Disagree
- ◯ Slightly Disagree
- ◯ Neither Agree nor Disagree
- ◯ Slightly Agree
- ◯ Agree
- ◯ Strongly Agree
- ◯ Very Strongly Agree
- ◯ I Don't Know

65) Highlighting positive stories about supportive end of life care provided within LTC could help change public perceptions about the quality of care provided within LTC.

- ◯ Very Strongly Disagree
- ◯ Strongly Disagree
- ◯ Disagree
- ◯ Slightly Disagree
- ◯ Neither Agree nor Disagree
- ◯ Slightly Agree
- ◯ Agree
- ◯ Strongly Agree
- ◯ Very Strongly Agree
- ◯ I Don't Know

66) Senior management within LTC facilities can dictate how supportive end of life care is prioritized within their facilities through policy and procedures, as well as by influencing culture.

- ◯ Very Strongly Disagree
- ◯ Strongly Disagree
- ◯ Disagree
- ◯ Slightly Disagree
- ◯ Neither Agree nor Disagree
- ◯ Slightly Agree
- ◯ Agree
- ◯ Strongly Agree
- ◯ Very Strongly Agree
- ◯ I Don't Know

67) Please provide additional comments here (optional)

____

Topics Related to Families (11 Statements)

68) It is important to involve families in conversations about care.

- ◯ Very Strongly Disagree
- ◯ Strongly Disagree
- ◯ Disagree
- ◯ Slightly Disagree
- ◯ Neither Agree nor Disagree
- ◯ Slightly Agree
- ◯ Agree
- ◯ Strongly Agree
- ◯ Very Strongly Agree
- ◯ I Don't Know

69) Family misperceptions of outcomes can make it challenging to meet their expectations of supportive end of life care treatments.

- ◯ Very Strongly Disagree
- ◯ Strongly Disagree
- ◯ Disagree
- ◯ Slightly Disagree
- ◯ Neither Agree nor Disagree
- ◯ Slightly Agree
- ◯ Agree
- ◯ Strongly Agree
- ◯ Very Strongly Agree
- ◯ I Don't Know

70) Family misunderstandings of supportive care in LTC causes them anxiety, worry, and confusion.

- ◯ Very Strongly Disagree
- ◯ Strongly Disagree
- ◯ Disagree
- ◯ Slightly Disagree
- ◯ Neither Agree nor Disagree
- ◯ Slightly Agree
- ◯ Agree
- ◯ Strongly Agree
- ◯ Very Strongly Agree
- ◯ I Don't Know

71) Physicians can help families better understand supportive care in LTC by encouraging them to consult credible on-line and written resources and support groups for more information.

- ◯ Very Strongly Disagree
- ◯ Strongly Disagree
- ◯ Disagree
- ◯ Slightly Disagree
- ◯ Neither Agree nor Disagree
- ◯ Slightly Agree
- ◯ Agree
- ◯ Strongly Agree
- ◯ Very Strongly Agree
- ◯ I Don't Know

72) Involving palliative care consultants can help improve family members understanding of supportive care, including helping to manage family expectations.

- ◯ Very Strongly Disagree
- ◯ Strongly Disagree
- ◯ Disagree
- ◯ Slightly Disagree
- ◯ Neither Agree nor Disagree
- ◯ Slightly Agree
- ◯ Agree
- ◯ Strongly Agree
- ◯ Very Strongly Agree
- ◯ I Don't Know

73) Family values are factors that determine the level of care provided.

- ◯ Very Strongly Disagree
- ◯ Strongly Disagree
- ◯ Disagree
- ◯ Slightly Disagree
- ◯ Neither Agree nor Disagree
- ◯ Slightly Agree
- ◯ Agree
- ◯ Strongly Agree
- ◯ Very Strongly Agree
- ◯ I Don't Know

74) Goals of Care assessment tools help families understand what supportive end of life care means in LTC.

- ◯ Very Strongly Disagree
- ◯ Strongly Disagree
- ◯ Disagree
- ◯ Slightly Disagree
- ◯ Neither Agree nor Disagree
- ◯ Slightly Agree
- ◯ Agree
- ◯ Strongly Agree
- ◯ Very Strongly Agree
- ◯ I Don't Know

75) Goals of Care assessment tools help physicians manage care expectations and create care plans with the family.

- ◯ Very Strongly Disagree
- ◯ Strongly Disagree
- ◯ Disagree
- ◯ Slightly Disagree
- ◯ Neither Agree nor Disagree
- ◯ Slightly Agree
- ◯ Agree
- ◯ Strongly Agree
- ◯ Very Strongly Agree
- ◯ I Don't Know

76) Families can be helpful when determining goals of care given their intimate knowledge of the patient's goals and values.

- ◯ Very Strongly Disagree
- ◯ Strongly Disagree
- ◯ Disagree
- ◯ Slightly Disagree
- ◯ Neither Agree nor Disagree
- ◯ Slightly Agree
- ◯ Agree
- ◯ Strongly Agree
- ◯ Very Strongly Agree
- ◯ I Don't Know

77) It is important to support patients and families by making it possible for them to access multidisciplinary care and support services.

- ◯ Very Strongly Disagree
- ◯ Strongly Disagree
- ◯ Disagree
- ◯ Slightly Disagree
- ◯ Neither Agree nor Disagree
- ◯ Slightly Agree
- ◯ Agree
- ◯ Strongly Agree
- ◯ Very Strongly Agree
- ◯ I Don't Know

78) Having someone in the facility with some degree of authority helps families trust the decision to transition the patient from chronic supportive medical care to supportive palliative end of life care.

- ◯ Very Strongly Disagree
- ◯ Strongly Disagree
- ◯ Disagree
- ◯ Slightly Disagree
- ◯ Neither Agree nor Disagree
- ◯ Slightly Agree
- ◯ Agree
- ◯ Strongly Agree
- ◯ Very Strongly Agree
- ◯ I Don't Know

79) Please provide additional comments here (optional) ______________________________
