# Supplementary material for: Multi-disciplinary supportive end of life care in long-term care: an integrative approach to improving end of life
Source: BMC Geriatr. 2021 May 22;21:326. doi: 10.1186/s12877-021-02271-1 (PMC8140573; doi:10.1186/s12877-021-02271-1)
Supplement: Supplementary file 2 — Additional file 2. [file 12877_2021_2271_MOESM2_ESM.pdf]

Delphi Questionnaire Second Round

Thank you for agreeing to participate in this, the second round of the Delphi process. Your participation is completely voluntary and you are free to withdraw from participating at any time.

The second round contains 4 questions and expires on May 31, 2019 at midnight

If you would like to review the median results of the group for the Delphi One Survey, please click on the link below.

[Attachment: "Delphi One Survey Median Results with Narrative Comments.pdf"]

Demographic Information

1) What is your role in long-term care (LTC)?
   - ◯ Family Physician
   - ◯ Specialist Physician
   - ◯ Nurse (Includes RN, LPN)
   - ◯ Health Care Aide
   - ◯ Healthcare provider (other than physician, RN, LPN, Health Care Aide)
   - ◯ LTC Manager/Administrator
   - ◯ Resident Living in LTC
   - ◯ Family Member of Resident Living in LTC
   - ◯ Researcher/Knowledge Translation Expert

2) What is your age?
   - ◯ Equal to or under 30
   - ◯ 31-39
   - ◯ 40-49
   - ◯ 50-59
   - ◯ 60-69
   - ◯ 70-79
   - ◯ 80-89
   - ◯ Equal to or over 90

3) In what province do you live?
   - ◯ Alberta
   - ◯ British Columbia
   - ◯ Manitoba
   - ◯ New Brunswick
   - ◯ Newfoundland and Labrador
   - ◯ Northwest Territories
   - ◯ Nova Scotia
   - ◯ Nunavit
   - ◯ Ontario
   - ◯ Prince Edward Island
   - ◯ Quebec
   - ◯ Saskatchewan
   - ◯ Yukon

4) Based on population size, is your primary place of residence/work located in an urban or rural setting?
   - ◯ Urban large size population (100,00 and over)
   - ◯ Urban medium population (between 30,000 and 99,999)
   - ◯ Urban small population (between 1000 and 29,999)
   - ◯ Rural - territory located outside any size urban area

Delphi Survey Round Two

Please indicate how strongly you agree or disagree with each statement below using the 10 point scale provided.

5) Staff lack the time needed to use pain assessment tools

- ◯ Very Strongly Disagree
- ◯ Strongly Disagree
- ◯ Disagree
- ◯ Slightly Disagree
- ◯ Neither Agree nor Disagree
- ◯ Slightly Agree
- ◯ Agree
- ◯ Strongly Agree
- ◯ Very Strongly Agree
- ◯ I Don't Know

6) LTC staff are uncomfortable with providing supportive end of life care.

- ◯ Very Strongly Disagree
- ◯ Strongly Disagree
- ◯ Disagree
- ◯ Slightly Disagree
- ◯ Neither Agree nor Disagree
- ◯ Slightly Agree
- ◯ Agree
- ◯ Strongly Agree
- ◯ Very Strongly Agree
- ◯ I Don't Know

7) LTC staff are unknowledgeable and/or inexperienced in supportive end of life care.

- ◯ Very Strongly Disagree
- ◯ Strongly Disagree
- ◯ Disagree
- ◯ Slightly Disagree
- ◯ Neither Agree nor Disagree
- ◯ Slightly Agree
- ◯ Agree
- ◯ Strongly Agree
- ◯ Very Strongly Agree
- ◯ I Don't Know

8) The best education for staff initially resistant to providing supportive end of life care in LTC is the practical knowledge they gain from managing symptoms in difficult situations

- ◯ Very Strongly Disagree
- ◯ Strongly Disagree
- ◯ Disagree
- ◯ Slightly Disagree
- ◯ Neither Agree nor Disagree
- ◯ Slightly Agree
- ◯ Agree
- ◯ Strongly Agree
- ◯ Very Strongly Agree
- ◯ I Don't Know
