# Supplementary material for: Multi-disciplinary supportive end of life care in long-term care: an integrative approach to improving end of life
Source: BMC Geriatr. 2021 May 22;21:326. doi: 10.1186/s12877-021-02271-1 (PMC8140573; doi:10.1186/s12877-021-02271-1)

Appendix C

The Behaviour Change Wheel Framework (Atkins et al., 2017; Michie, van Stralen, & West, 2011)

COM-B	Education	Persuasion	Training	Restrictions	Environment	Modelling	Enablement
**Physical capability**							
**Psychological capability**							
**Physical opportunity**							
**Social opportunity**							
**Automatic motivation**							
**Reflective motivation**							

Interventions	Purpose	Strategies/Practices (Examples)
**Education**	To increase knowledge or improve understanding	Providing information to promote healthy eating
**Persuasion**	To induce positive or negative feelings and through this, stimulate action	Using picture, stories, or testimonials to increase physical exercise
**Incentivism**	To create an expectation of reward	Offering prizes to induce attempts to stop smoking
**Coercion**	To create an expectation of cost or punishment	Raising the cost to decrease alcohol use
**Training**	To increase practical skills	Providing advanced driver training to increase safe driving
**Restriction**	Use rules to reduce the possibility of engaging in a target behavior	Prohibiting the availability of intoxicating substances to youth to reduce opportunities for intoxication
**Restriction**	Use rules to increase the possibility of engaging in a target behavior by reducing the opposite behavior	Increasing the availability of positive activities such as volunteerism to fill time, build friendships, etc and reduce substance use
**Environmental Restructuring**	To change the physical or social context	Building prompts into computer software programs to remind care providers to ask about smoking habits
**Modelling**	To give people an example of the ideal to aspire to	Incorporating safe-sex practices into TV drama scenes to increase condom use
**Enablement**	To increase the means or reduce the barriers in order to increase a capability or opportunity	Providing behavior modification support for smoking cessation; provide prosthesis to increase physical activity; provide standing desks to increase energy use, provide cycling-telephone charging stations to increase energy use

Policies (Types)	Definition	Strategies (Examples)
**Communication/Marketing**	Using print, electronic, telephone, or broadcast media to increase awareness	Developing and implementing a mass media campaign
**Guidelines**	Creating documents that mandate changes to service provision	Developing and disseminating treatment protocols
**Fiscal**	Using the taxation system to decrease or increase the financial cost of the target behavior	Increasing duty to prevent taking goods across the border; Increasing anti-smuggling surveillance
**Regulation**	Establishing and regulate rules or principles of right behaviour	Establishing voluntary agreements on advertising that messages the behaviour
**Legislation**	Making or changing laws	Prohibiting sale or use of a product or service
**Environmental/Social Planning**	Designing or controlling the physical or social environment	Using town or facility planning
**Service Provision**	Delivering a service	Establishing a support service in the workplace

Atkins, L., Francis, J., Islam, R., O'Connor, D., Patey, A., Ivers, N., . . . Michie, S. (2017). A guide to using the Theoretical Domains Framework of behaviour change to investigate implementation problems. *Implementation Science, 12*(1), 77. doi:10.1186/s13012-017-0605-9

Michie, S., van Stralen, M. M., & West, R. (2011). The behaviour change wheel: a new method for characterising and designing behaviour change interventions. *Implement Sci, 6*, 42. doi:10.1186/1748-5908-6-42

Barriers and Facilitators - Implementation and Activity Guide

COM-B and the Theoretical Domains Framework (TDF)

COM-B	TDF
Physical Capability	Physical Skills
Psychological Capability	Knowledge<br>Cognitive& interpersonal skills<br>Memory, attention& decision processes<br>Behavioural regulation
Physical Opportunity	Environmental context & resources
Social Opportunity	Social influences
Reflective Motivation	Professional/social role & identify<br>Beliefs about capabilities<br>Optimism<br>Beliefs about consequences<br>Intentions<br>Goals
Automatic Motivation	Reinforcement<br>Emotion

Mapping Implementation Strategies to help address Perceived Barriers

BARRIER	IMPLEMENTATION ACTIVITIES/STRATEGIES
Perceived lack of skills	- Training on how to use tools/processes
Lack of knowledge	- Education about tools/processes and when & why to use them
Lack of accountability	- Education about when & why to use tools/processes<br>- Role Modelling by Champions/Opinion Leaders<br>- Use documentation/reporting to increase the opportunity to engage in the use of tools/processes
Staff attitudes and beliefs about capabilities/consequences	- Education about when & why to use tools/processes<br>- Role Modelling by Champions/Opinion Leaders<br>- Increase means/decrease barriers to using the tools/processes
Lack of clarity regarding roles and responsibilities	- Education about when & why to use tool/processes and who should use them<br>- Role Modelling by Champions/Opinion Leaders<br>- Clear communication (verbal and written) about roles and responsibilities
Presences of other priorities and initiatives at the facility/ on the unit	- Restructure the environment to make it easy to use the tools/processes within the current work flow/work processes<br>- Role Modelling by Champions/Opinion Leaders<br>- Increase means/decrease barriers to using tools/processes<br>- Use documentation/reporting to increase the opportunity to engage in the use of tools/processes
Existing Climate/Culture of facility/unit	- Restructure the environment to make it easy to use the tools/processes within the current work flow/work processes<br>- Role Modelling by Champions/Opinion Leaders<br>- Increase means/decrease barriers to using tools/processes<br>- Use documentation/reporting to increase the opportunity to engage in the use of tools/processes
Resident/Family beliefs	- Resident/Family Education (written/verbal)<br>- Using communication to induce positive feelings about the change
Lack of communication between health care providers regarding patient's care plan	- Increase means/decrease barriers to communication<br>- Restructure the environment to make it easy to use tools/processes within the work flow/work processes
Time constraints/heavy workload	- Restructure the environment to make it easy to use tools/processes within the current work flow/work processes
Resistance to implement intervention	- Education about when & why to use tools/processes<br>- Role Modelling by Champions/Opinion Leaders<br>- Use documentation/reporting to increase the opportunity to engage in the use of tools/processes<br>- Create incentives to use the tools/processes (e.g. rewards/recognition)

Appendix 11

Worksheet: Managing Total Pain and Other Symptoms to Optimize Quality of Life

Delphi Statements	Barrier to Optimal Care (B) or Facilitator to Optimal Care (F)	Intervention (Michie TDF) or Policy	Strategy (example)
1. It is challenging to distinguish symptoms (i.e. confusion) related to pain from behaviours (i.e. confusion) related to dementia.			
2. Pain assessment tools (E.G. PAINAD [Pain Assessment in Advanced Dementia]; PACSLAC [Pain Assessment Checklist]) are useful for measuring pain level in LTC residents with dementia.			
3. Physicians find it challenging to appropriately manage pain if it is not documented in the patient's chart.			

Delphi Statements	Barrier to Optimal Care (B) or Facilitator to Optimal Care (F)	Intervention (Michie TDF) or Policy	Strategy (example)
4. The adverse effects of opiates/narcotics, can make treating pain challenging in frail LTC residents.			
5. LTC staff are often inexperienced giving narcotic medication, particularly when faced with "prn" (or as needed) medication orders for patients nearing end of life.			
6. Access to technologies within LTC facilities (e.g.continuous IV pumps; oxygen masks; catheters to manage malignant effusions) can help facilitate supportive end of life care.			

Delphi Statements	Barrier to Optimal Care (B) or Facilitator to Optimal Care (F)	Intervention (Michie TDF) or Policy	Strategy (example)
**7. When LTC residents are nearing the end of their lives but not imminently dying, they should be given more opportunities to participate in physical activity.**			
**8. 10. Regular mobilization and maintaining independence in basic activities of daily living should be a priority in LTC**			
**9. Occupational therapy involvement is helpful for identifying and addressing functional changes throughout LTC admission to end of life.**			
**10. Recreational activities such as gardening, caring for plants and animals, telling stories, and hearing children sing are helpful for managing pain and/or anxiety.**			

Delphi Statements	Barrier to Optimal Care (B) or Facilitator to Optimal Care (F)	Intervention (Michie TDF) or Policy	Strategy (example)
**11. Having the ability to access physicians in the emergency department for advice about acute medical issues, by phone on an as-needed basis, can help avoid transfer to hospital.**			
**12. When caring for LTC residents, it is challenging to balance the management of several diseases (each of which often requires several medications) with the expectation in LTC to reduce the total number of medications.**			
**13. Having access to specialist physician advice via phone can improve care.**			

Delphi Statements	Barrier to Optimal Care (B) or Facilitator to Optimal Care (F)	Intervention (Michie TDF) or Policy	Strategy (example)
**14. Medication choices within LTC should focus more on pain and symptom management, and less on longer- term preventative care (e.g. lipid lowering; tight blood glucose control; blood pressure management).**			
**15. Considering the short and long-term impact of medication on functional ability helps LTC physicians choose the appropriate types and numbers of medications.**			
**16. Family can be engaged in helping to deliver non-medication (i.e. behavioural) interventions for residents in LTC**			

Delphi Statements	Barrier to Optimal Care (B) or Facilitator to Optimal Care (F)	Intervention (Michie TDF) or Policy	Strategy (example)
**17. Having end of life orders sets can help guide supportive care decision-making once the patient is identified as needing palliative care.**			
**18. Rather than (or in addition to) treating their symptoms with medication, providing space for residents to move and express themselves can help manage stress and anxiety.**			
**19. The CAM (Confusion Assessment Method) is useful for detecting delirium in LTC residents with dementia.**			

Delphi Statements	Barrier to Optimal Care (B) or Facilitator to Optimal Care (F)	Intervention (Michie TDF) or Policy	Strategy (example)
20. Among persons with dementia, it is important to identify the underlying factors associated with a patient's responsive behaviors (e.g. aggression, calling out).			
21. Among persons with dementia, it is important to include the family when trying to understand a patient's responsive behaviours.			
22. Inconsistencies in how different LTC facilities chart symptoms can make it challenging for physicians and other healthcare providers that work within multiple different LTC facilities to care for residents.			
23. Inconsistencies in how different LTC facilities chart symptoms can make it challenging for physicians			

Delphi Statements	Barrier to Optimal Care (B) or Facilitator to Optimal Care (F)	Intervention (Michie TDF) or Policy	Strategy (example)
**and other healthcare providers that work within multiple different LTC facilities to communicate with other team members**			

Worksheet: The Context of Providing Supportive End of Life Care within LTC

Delphi Statement	Barrier to Optimal Care (B) or Facilitator to Optimal Care (F)	Intervention (Michie TDF) or Policy	Strategy (example)
1. There is a lack of mental health care supports (e.g. Psychiatrist, Psychologist, SW) within LTC for residents with mental health conditions other than dementia.			

Delphi Statement	Barrier to Optimal Care (B) or Facilitator to Optimal Care (F)	Intervention (Michie TDF) or Policy	Strategy (example)
2. Spiritual care providers working in LTC should have an understanding of frailty.			
3. Increased access to spiritual care services can improve end of life care outcomes.			
4. LTC residents frequently end up being transferred to acute care facilities as the result of inadequate staff to patient ratios needed to manage the increasing care needs of those nearing the end of life.			
5. Providing LTC staff with education and training about supportive end of life care improves end of life care practices.			
6. Physicians can support LTC staff by encouraging them to attend palliative care education sessions on-line or in person,			

Delphi Statement	Barrier to Optimal Care (B) or Facilitator to Optimal Care (F)	Intervention (Michie TDF) or Policy	Strategy (example)
and advocating to management for the time and money LTC staff need in order to participate.			
7. Access to palliative care consultants improves the provision of end of life care in LTC.			
8. By working with palliative care consultants, LTC physicians learn to utilize palliative (supportive) care principles and practices to treat symptoms and care for residents from admission to end-of-life.			
9. It can be difficult to understand and manage the diverse ethnic values and attitudes of LTC staff toward death.			
10. When they exist within a LTC facility, end of life			

Delphi Statement	Barrier to Optimal Care (B) or Facilitator to Optimal Care (F)	Intervention (Michie TDF) or Policy	Strategy (example)
protocols/pathways are followed inconsistently.			
11. There are gaps in physician training and education in terms of the application of supportive end of life care practices within the LTC setting.			
12. There are gaps in physician training and education in terms of the emotional aspects of delivering supportive end of life care in LTC.			
13. LTC physicians often do not consult palliative care early enough in a patient's end of life care path.			

Delphi Statement	Barrier to Optimal Care (B) or Facilitator to Optimal Care (F)	Intervention (Michie TDF) or Policy	Strategy (example)
14. LTC physicians' ability to consult with colleagues external to the LTC facility is a facilitator to providing care (e.g. physician colleagues, EMS, Nurse Practitioners, Psychiatrist Services, Geriatric Mental Health Services, Occupational Therapist, Physiotherapist, and Dental services).			
15. Having access to staff and colleagues trained in the application of supportive end of life care helps the LTC physician support the patient and family.			
16. Nurse practitioners can help relieve the supportive end of life care burden in LTC.			

Delphi Statement	Barrier to Optimal Care (B) or Facilitator to Optimal Care (F)	Intervention (Michie TDF) or Policy	Strategy (example)
17. Changes in funding models for primary care physicians to work in LTC can improve physician involvement in LTC.			
18. Public knowledge and negative perceptions about the quality of care provided within LTC influences a person's decision to attain a higher level of care at end of life (e.g. transfer to hospital).			
19. Highlighting positive stories about supportive end of life care provided within LTC could help change public perceptions about the quality of care provided within LTC.			

Delphi Statement	Barrier to Optimal Care (B) or Facilitator to Optimal Care (F)	Intervention (Michie TDF) or Policy	Strategy (example)
20. Senior management within LTC facilities can dictate how supportive end of life care is prioritized within their facilities through policy and procedures, as well as by influencing culture.			

Worksheet: Topics Related to Families

Delphi Statements	Barrier to Optimal Care (B) or Facilitator to Optimal Care (F)	Intervention (Michie TDF) or Policy	Strategy (example)
1. It is important to involve families in conversations about care.			

Delphi Statements	Barrier to Optimal Care (B) or Facilitator to Optimal Care (F)	Intervention (Michie TDF) or Policy	Strategy (example)
2. Family misperceptions of outcomes can make it challenging to meet their expectations of supportive end of life care treatments.			
3. Family misunderstandings of supportive care in LTC causes them anxiety, worry, and confusion.			
4. Physicians can help families better understand supportive care in LTC by encouraging them to consult credible on-line and written resources and support groups for more information.			
5. Involving palliative care consultants can help improve family members understanding of supportive care, including helping to manage family expectations.			

**Delphi Statements**	**Barrier to Optimal Care (B) or Facilitator to Optimal Care (F)**	**Intervention (Michie TDF) or Policy**	**Strategy (example)**
6. Family values are factors that determine the level of care provided.			
7. Goals of Care assessment tools help families understand what supportive end of life care means in LTC.			
8. Goals of Care assessment tools help physicians manage care expectations and create care plans with the family.			
9. Families can be helpful when determining goals of care given their intimate knowledge of the patient's goals and values.			
10. It is important to support patients and families by making it possible for them to access multidisciplinary care and support services.			

**Delphi Statements**	**Barrier to Optimal Care (B) or Facilitator to Optimal Care (F)**	**Intervention (Michie TDF) or Policy**	**Strategy (example)**
11. Having someone in the facility with some degree of authority helps families trust the decision to transition the patient from chronic supportive medical care to supportive palliative end of life care.			

Worksheet: Managing End of Life

**Delphi Statement**	**Barrier to Optimal Care (B) or Facilitator to Optimal Care (F)**	**Intervention (Michie TDF) or Policy**	**Strategy (example)**
1. It can be challenging to determine where a frail resident is on the life-expectancy trajectory (or			

**Delphi Statement**	**Barrier to Optimal Care (B) or Facilitator to Optimal Care (F)**	**Intervention (Michie TDF) or Policy**	**Strategy (example)**
curve), making it hard to know when to transition from interventional medical care to palliative care.			
**2. Having a standardized frailty assessment tool that is used on a routine basis can help judge a patient's health status from LTC admission up until end of life.**			
3. The CHESS (Changes in Health, End-Stage Disease, Signs and Symptoms) score can be generated from data already collected within LTC and thus could be helpful in determining when to initiate end of life care if made available for each LTC resident.			
4. The care needs of residents increase as they reach the end			

Delphi Statement	Barrier to Optimal Care (B) or Facilitator to Optimal Care (F)	Intervention (Michie TDF) or Policy	Strategy (example)
of their life, yet overall staffing levels remain the same.			
5. Timely identification by LTC staff of residents whose condition has deteriorated and/or who appear at high risk of imminent death, and communicating this to the attending LTC physician can improve outcomes at end of life.			
**6. It can be difficult to access multidisciplinary support in my community (e.g. psychologists, SW, chaplaincy, chronic pain service).**			
7. Engaging residents and families in discussions about goals of care, treatment preferences and palliative care needs can			

Delphi Statement	Barrier to Optimal Care (B) or Facilitator to Optimal Care (F)	Intervention (Michie TDF) or Policy	Strategy (example)
improve end of life care outcomes.			
8. A collaborative approach to care improves care by improving communication between team members.			
9. A documented palliative care pathway helps to identify changes in patient status, manage family expectations, set staff expectations, and organize the provision of care.			
10. It is important to determine and include family expectations in care decision making, and not rely exclusively on standardized care management protocols.			
11. It is important to have private space within the LTC facility for			

Delphi Statement	Barrier to Optimal Care (B) or Facilitator to Optimal Care (F)	Intervention (Michie TDF) or Policy	Strategy (example)
the patient and family at or near end of life.			

Delphi Questionnaire Description and Summary: Improving Supportive End-of-Life Care in Long-Term Care

The Delphi One Survey statements were developed from the analysis of data obtained from interviews with 23 physicians practicing within Long Term Care Facilities (LTC) in Alberta and/or from a systematic review of the literature related to end of life or palliative care models and practices within LTC. The results of the Delphi process will be used to determine what aspects are most important to include in end of life pathways and/or processes within LTC. For the purpose of the Delphi survey, LTC staff is defined as non-physician health care providers (e.g. nurse; health care aides).

The Delphi One Survey contained 4 demographic questions and 75 statements sorted into 4 categories. It was distributed to 65 individuals representing Content Experts, Physicians, Nurse Administrators, Health Care Aides, Allied Health Professionals.

We received 18 responses that included 17 on-responses and 1 pen and paper version. All group results were reviewed and median results with standard error calculated. Our established threshold for appropriateness was when the median score for a statement was greater than or equal to 7 and inappropriate if less than 3. Respondents assessed 71 of the 75 statements appropriate. None were deemed inappropriate. Four (4) were inconclusive.

Round Two of the survey included the 4 inconclusive Delphi Round One responses with a median score of more than 3 and less than 7. We sent the Delphi Round Two Survey to the same 17 Delphi One Survey on-line respondents. Delphi Two Survey participants also received a personalized questionnaire with their prior rating, the median results of the group and a compilation of the narrative comments. We did not have ethics approval to collect the names and contact information of the Delphi One pen and paper respondent. We were unable, therefore, include this individual in the Delphi Two Survey.

We received 11 responses to the Delphi Two Survey Questionnaire. Results were reviewed for appropriateness and agreement. None of the 4 statements scored less than 3 or more than 7. These 4 statements were then discussed among the research team. A statement was deemed inappropriate if at least one third of the discussion participants rated the item opposite to their peers.

Delphi One Questionnaire Median Results

All data (all records and fields)
Legend
(Counts, Frequency)

Very Strongly Disagree
Strongly Disagree
Disagree
Slightly Disagree
Neither Agree nor Disagree
Slightly Agree
Agree
Strongly Agree
Very Strongly Agree
I Don't Know

Demographics

1. What is your role in long-term care (LTC)?

Family Physician (5, 25.0%),

Specialist Physician (1, 5.0%),

Nurse (Includes RN, LPN) (2, 10.0%),

Health Care Aide (1, 5.0%),

Healthcare provider (other than physician, RN, LPN, Health Care Aide) (0, 0.0%),

LTC Manager/Administrator (4, 20.0%),

Resident Living in LTC (1, 5.0%),

Family Member of Resident Living in LTC (2, 10.0%),

Researcher/Knowledge Translation Expert (4, 20.0%)

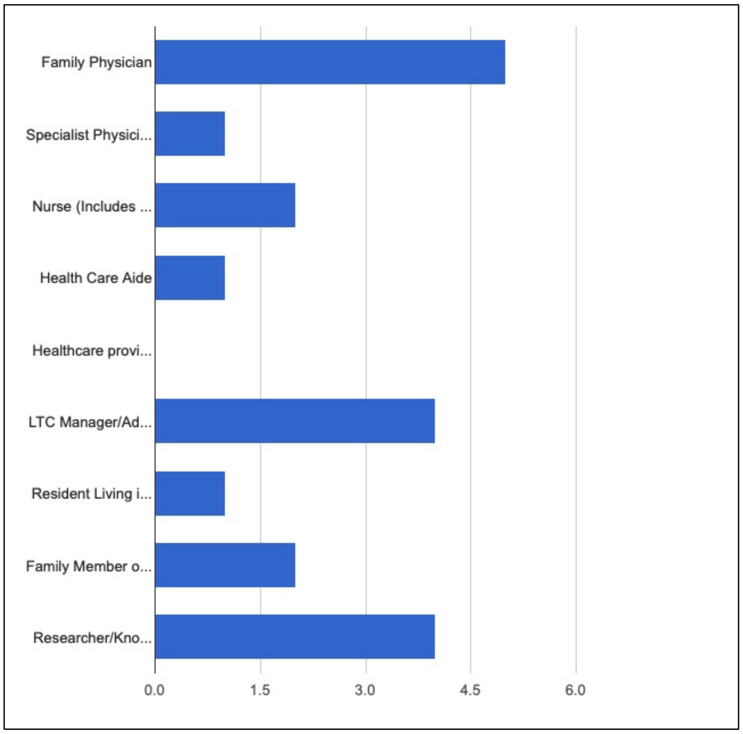

2. What is your age?

Equal to or under 30 (1, 5.0%),

31-39 (2, 10.0%),

40-49 (3, 15.0%),

50-59 (9, 45.0%),

60-69 (3, 15.0%),

70-79 (2, 10.0%),

80-89 (0, 0.0%),

Equal to or over 90

(0, 0.0%)

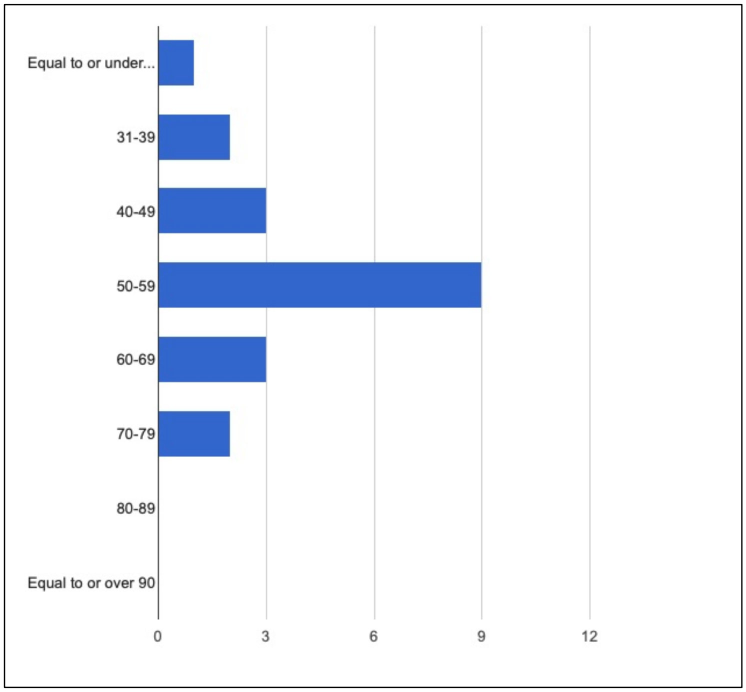

3. In what province do you live?

Alberta (18, 90.0%),

British Columbia (0, 0.0%),

Manitoba (1, 5.0%),

New Brunswick (0, 0.0%),

Newfoundland and Labrador (0, 0.0%),

Northwest Territories (0, 0.0%),

Nova Scotia (0, 0.0%),

Nunavit (0, 0.0%),

Ontario (1, 5.0%),

Prince Edward

Island (0, 0.0%),

Quebec (0, 0.0%),

Saskatchewan (0, 0.0%),

Yukon (0, 0.0%)

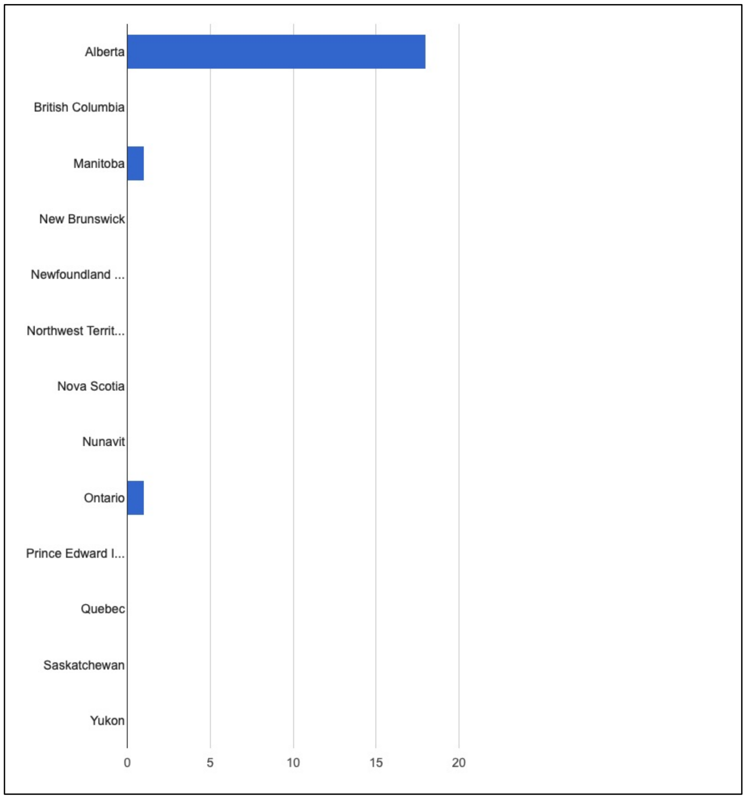

4. Based on population size, is your primary place of residence/work located in an urban or rural setting?

Urban large size population

(100,00 and over) (17, 85.0%),

Urban medium population

(between 30,000 and 99,999) (1, 5.0%),

Urban small population

(between 1000 and 29,999) (2, 10.0%),

Rural - territory located outside

any size urban area

(0, 0.0%)

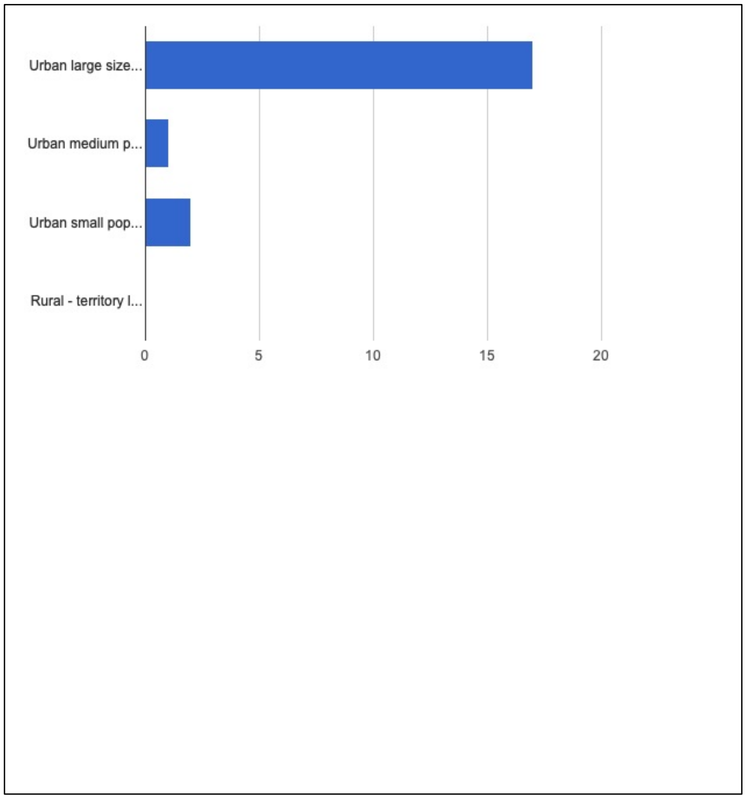

Managing Total Pain and Other Symptoms to Optimize Quality of Life (11 statements)

5. It is challenging to distinguish symptoms (i.e. confusion) related to pain from behaviours (i.e. confusion) related to dementia.

Very Strongly Disagree (1, 5.3%),

Strongly Disagree (0, 0.0%),

Disagree (2, 10.5%),

Slightly Disagree (0, 0.0%),

Neither Agree nor Disagree (3, 15.8%),

Slightly Agree (2, 10.5%),

Agree (7, 36.8%),

Strongly Agree (2, 10.5%),

Very Strongly Agree (1, 5.3%),

I Don't Know (1, 5.3%)

Median Score 7

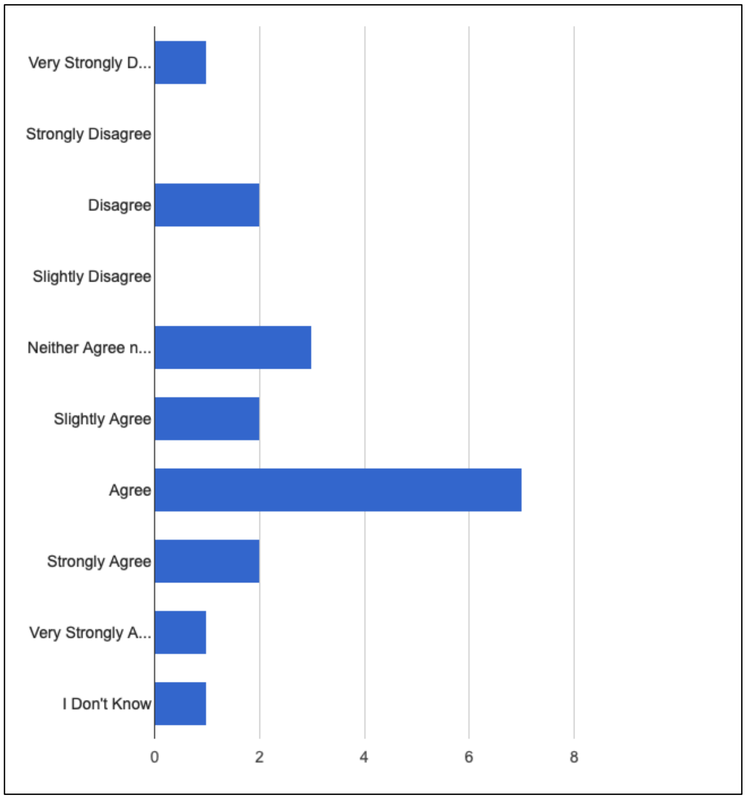

6. Pain assessment tools (E.G. PAINAD [Pain Assessment in Advanced Dementia]; PACSLAC [Pain Assessment Checklist]) are useful for measuring pain level in LTC residents with dementia.

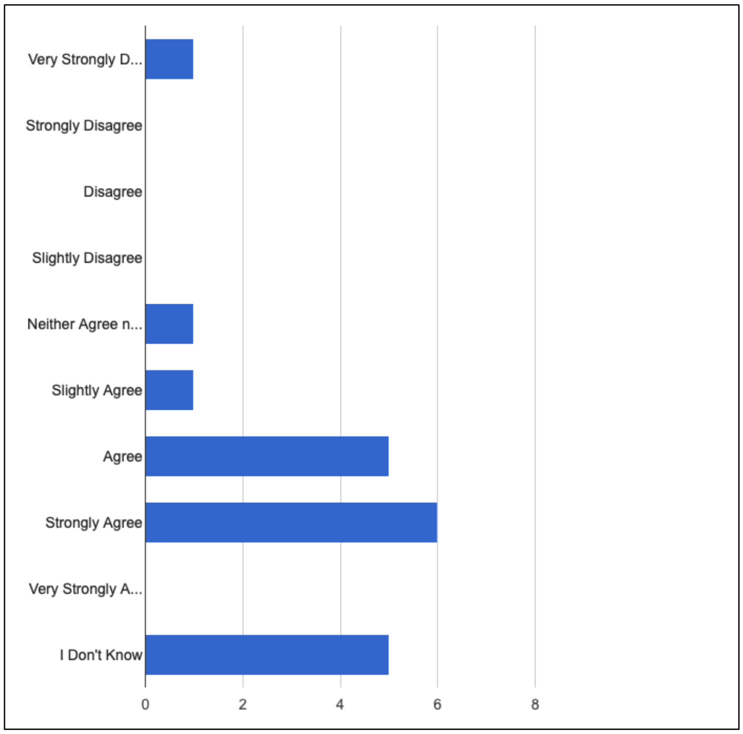

Very Strongly Disagree (1, 5.3%),

Strongly Disagree (0, 0.0%),

Disagree (0, 0.0%),

Slightly Disagree (0, 0.0%),

Neither Agree nor Disagree (1, 5.3%),

Slightly Agree (1, 5.3%),

Agree (5, 26.3%),

Strongly Agree (6, 31.6%),

Very Strongly Agree (0, 0.0%),

I Don't Know (5, 26.3%)

Median Score 7

7. Staff lack the time needed to use pain assessment tools.

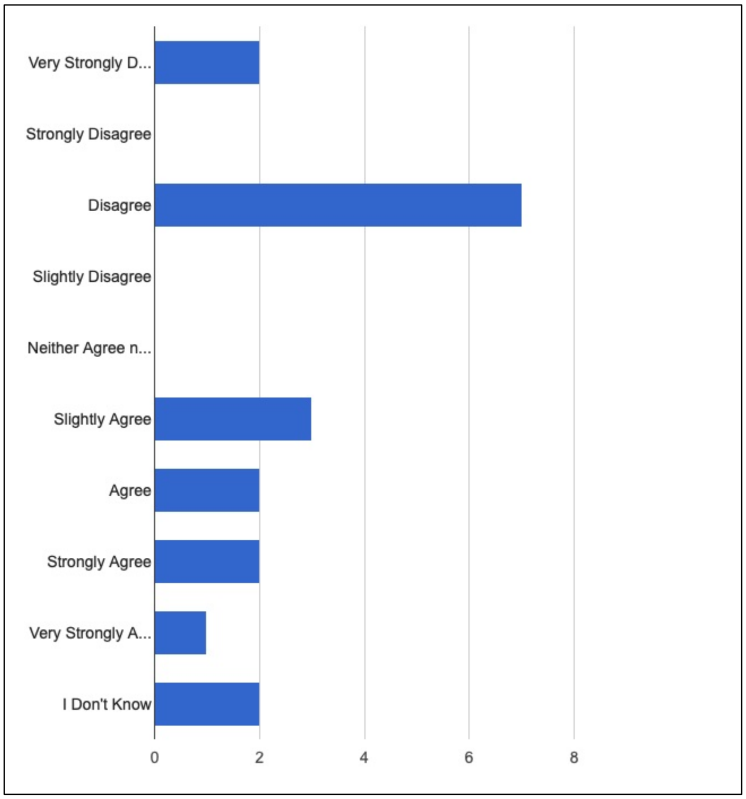

Very Strongly Disagree (2, 10.5%),

Strongly Disagree (0, 0.0%),

Disagree (7, 36.8%),

Slightly Disagree (0, 0.0%),

Neither Agree nor Disagree (0, 0.0%),

Slightly Agree (3, 15.8%),

Agree (2, 10.5%),

Strongly Agree (2, 10.5%),

Very Strongly

Agree (1, 5.3%),

I Don't Know (2, 10.5%)

Median Score 4.5

8. Physicians find it challenging to appropriately manage pain if it is not documented in the patient's chart.

Very Strongly Disagree (1, 5.3%),

Strongly Disagree (0, 0.0%),

Disagree (0, 0.0%),

Slightly Disagree (0, 0.0%),

Neither Agree nor Disagree (0, 0.0%),

Slightly Agree (0, 0.0%),

Agree (9, 47.4%),

Strongly Agree (7, 36.8%),

Very Strongly Agree

(1, 5.3%),

I Don't Know (1, 5.3%)

Median Score 7

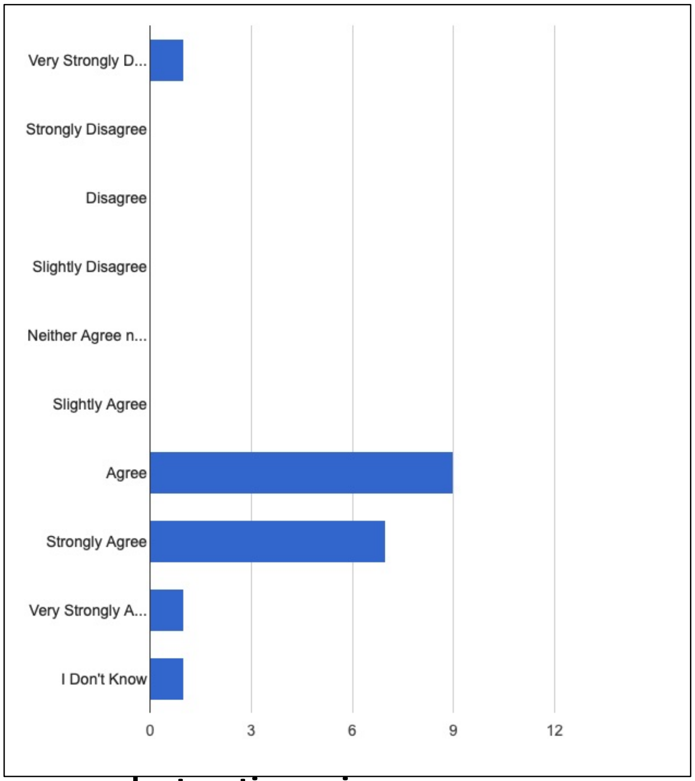

9. The adverse effects of opiates/narcotics, can make treating pain challenging in frail LTC residents.

Very Strongly Disagree (1, 5.3%),

Strongly Disagree (0, 0.0%),

Disagree (1, 5.3%),

Slightly Disagree (1, 5.3%),

Neither Agree nor Disagree (0, 0.0%),

Slightly Agree (2, 10.5%),

Agree (8, 42.1%),

Strongly Agree (5, 26.3%),

Very Strongly

Agree (1, 5.3%),

I Don't Know (0, 0.0%)

Median Score 7

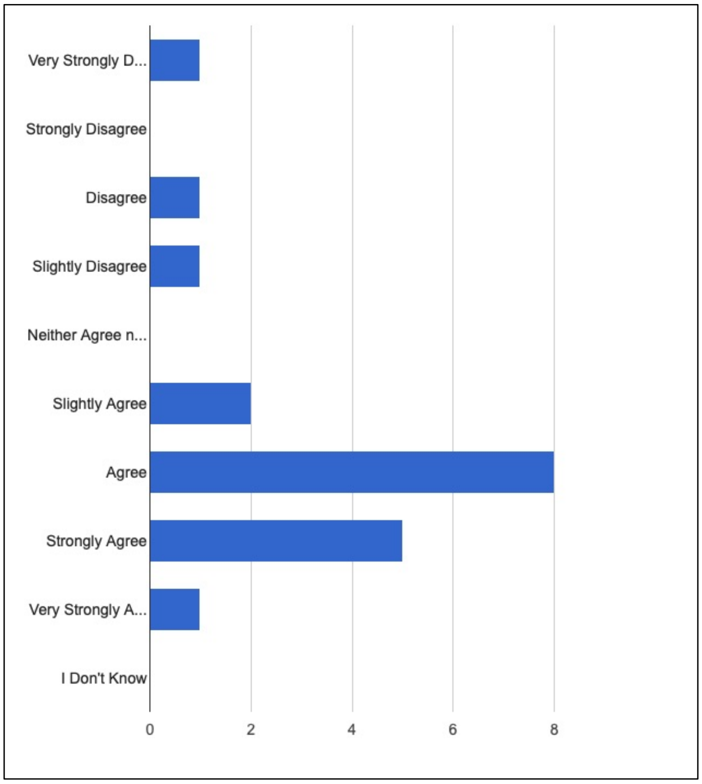

10. LTC staff are often inexperienced giving narcotic medication, particularly when faced with "prn" (or as needed) medication orders for patients nearing end of life.

Very Strongly Disagree (1, 5.3%),

Strongly Disagree (0, 0.0%),

Disagree (2, 10.5%),

Slightly Disagree (1, 5.3%),

Neither Agree nor Disagree (1, 5.3%),

Slightly Agree (1, 5.3%),

Agree (5, 26.3%),

Strongly Agree (5, 26.3%),

Very Strongly Agree

(1, 5.3%),

I Don't Know (2, 10.5%)

Median Score: 7

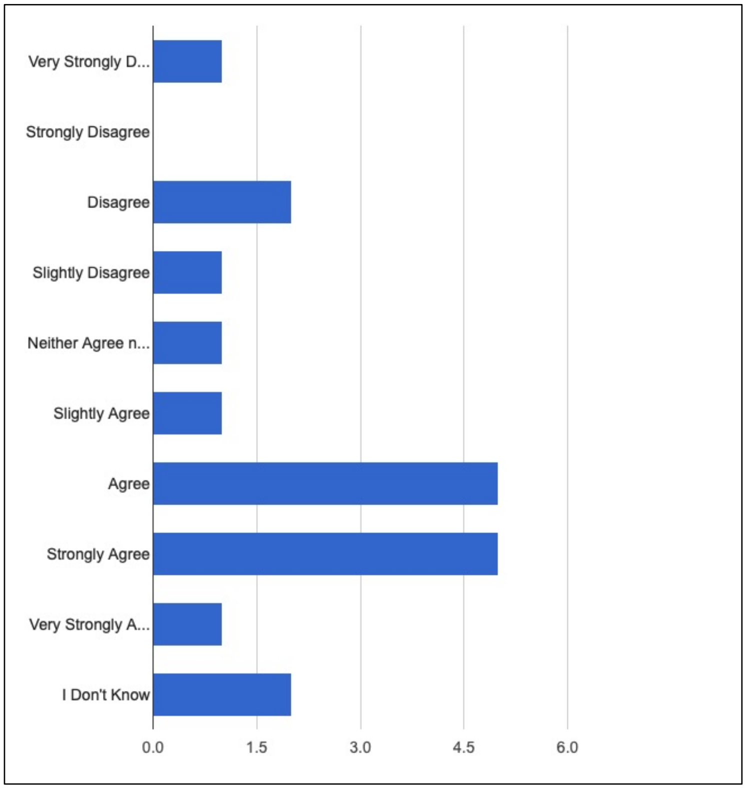

11. Access to technologies within LTC facilities (e.g.continuous IV pumps; oxygen masks; catheters to manage malignant effusions) can help facilitate supportive end of life care.

Very Strongly Disagree (1, 5.3%),

Strongly Disagree (1, 5.3%),

Disagree (0, 0.0%),

Slightly Disagree (0, 0.0%),

Neither Agree nor Disagree (0, 0.0%),

Slightly Agree (0, 0.0%),

Agree (7, 36.8%),

Strongly Agree (7, 36.8%),

Very Strongly Agree (2, 10.5%),

I Don't Know (1, 5.3%)

Median Score 8

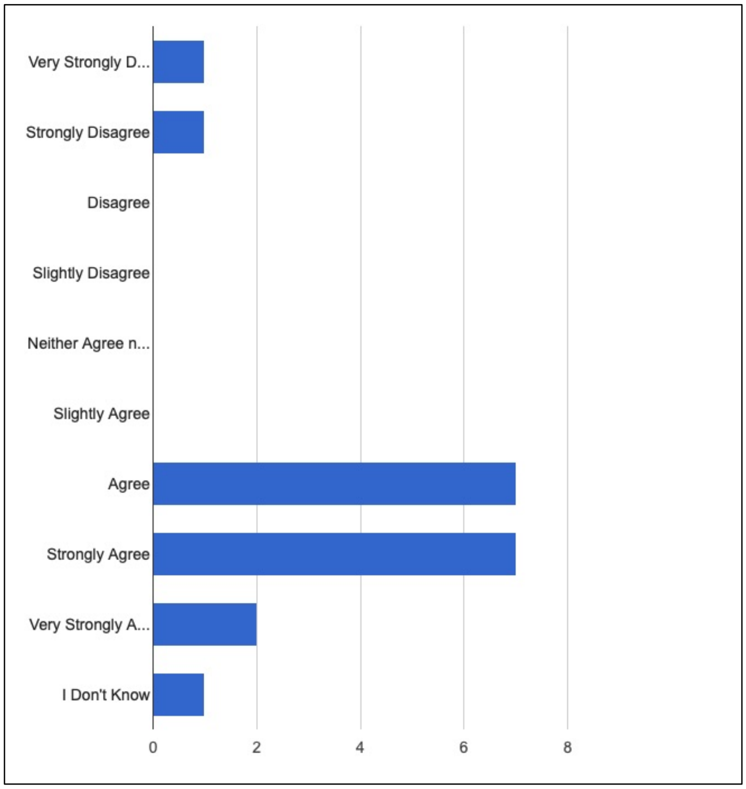

12. When LTC residents are nearing the end of their lives but not imminently dying, they should be given more opportunities to participate in physical activity.

Very Strongly Disagree (1, 5.3%),

Strongly Disagree (0, 0.0%),

Disagree (1, 5.3%),

Slightly Disagree (0, 0.0%),

Neither Agree nor Disagree (2, 10.5%),

Slightly Agree (2, 10.5%),

Agree (4, 21.1%),

Strongly Agree (7, 36.8%),

Very Strongly

Agree (1, 5.3%),

I Don't Know (1, 5.3%

Median Score: 7

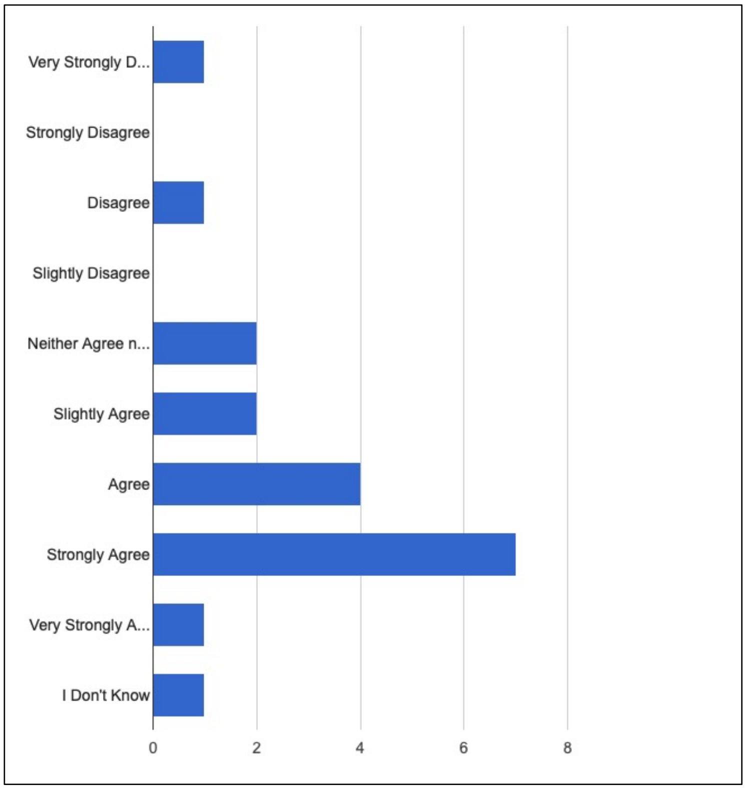

13. Regular mobilization and maintaining independence in basic activities of daily living should be a priority in LTC.

Very Strongly Disagree (1, 5.3%),

Strongly Disagree (0, 0.0%),

Disagree (0, 0.0%),

Slightly Disagree (0, 0.0%),

Neither Agree nor Disagree (1, 5.3%),

Slightly Agree (0, 0.0%),

Agree (4, 21.1%),

Strongly Agree (6, 31.6%),

Very Strongly Agree

(7, 36.8%),

I Don't Know (0, 0.0%)

Median Score 7

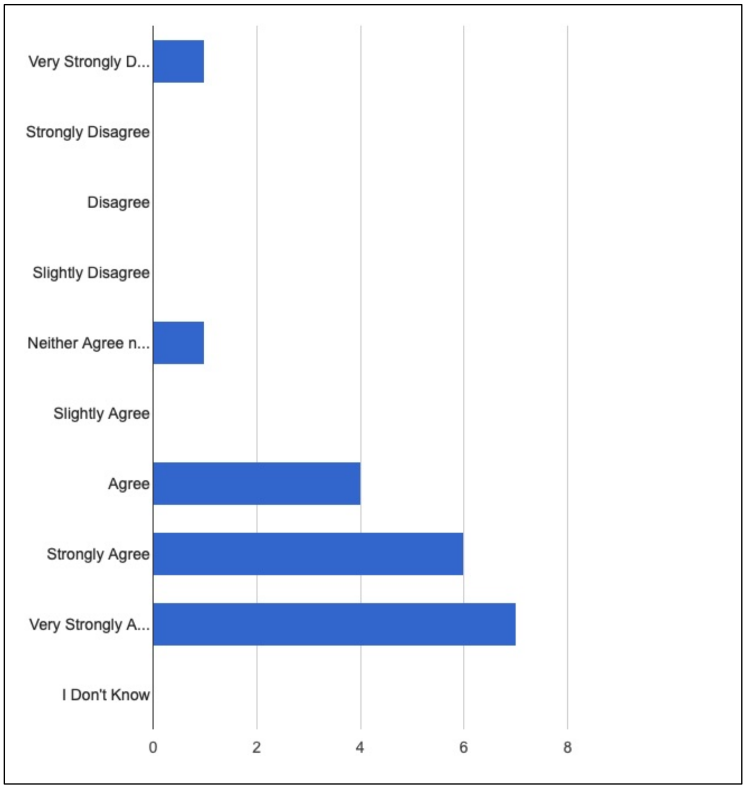

14. Occupational therapy involvement is helpful for identifying and addressing functional changes throughout LTC admission to end of life.

Very Strongly Disagree (1, 5.3%),

Strongly Disagree (0, 0.0%),

Disagree (1, 5.3%),

Slightly Disagree (0, 0.0%),

Neither Agree nor Disagree (1, 5.3%),

Slightly Agree (0, 0.0%),

Agree (5, 26.3%),

Strongly Agree (7, 36.8%),

Very Strongly Agree

(4, 21.1%),

I Don't Know (0, 0.0%)

Median Score 8

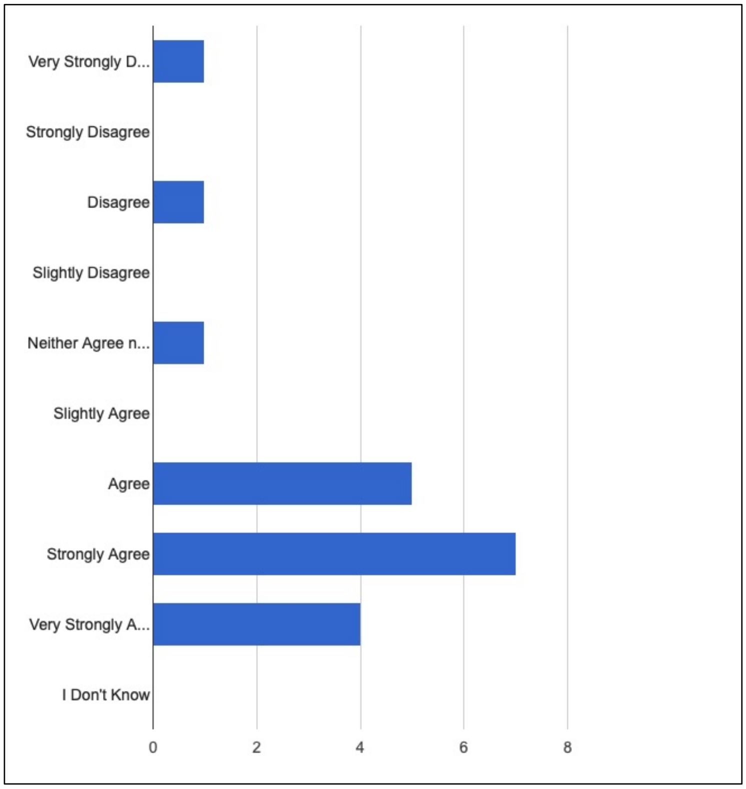

15. Recreational activities such as gardening, caring for plants and animals, telling stories, and hearing children sing are helpful for managing pain and/or anxiety.

Very Strongly Disagree (1, 5.3%),

Strongly Disagree (0, 0.0%),

Disagree (0, 0.0%),

Slightly Disagree (0, 0.0%),

Neither Agree nor Disagree (0, 0.0%),

Slightly Agree (0, 0.0%),

Agree (5, 26.3%), Strongly Agree (5, 26.3%),

Very Strongly Agree

(8, 42.1%),

I Don't Know (0, 0.0%)

Median Score 8

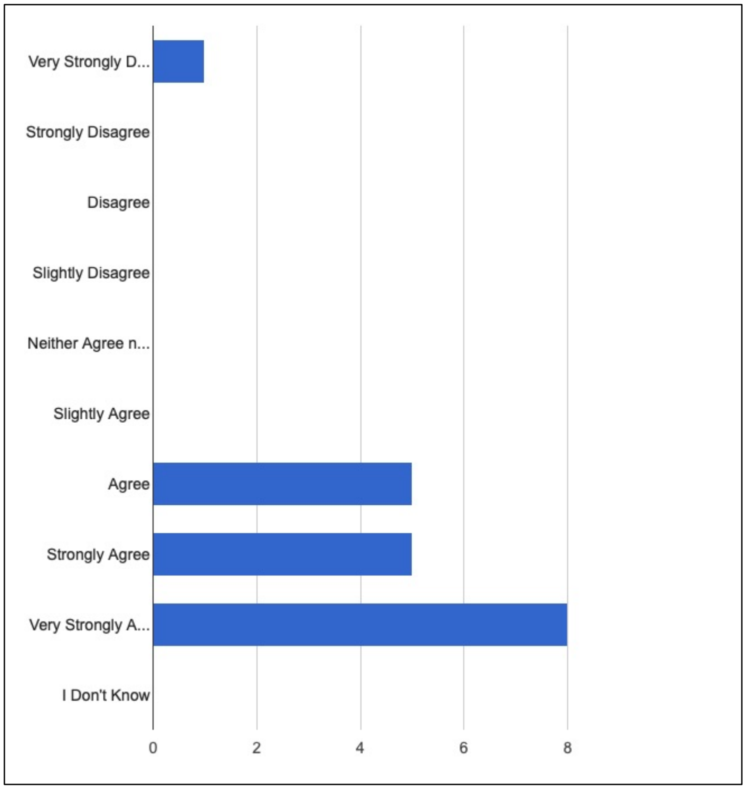

17. Having the ability to access physicians in the emergency department for advice about acute medical issues, by phone on an as-needed basis, can help avoid transfer to hospital.

Very Strongly Disagree (1, 5.6%),

Strongly Disagree (0, 0.0%),

Disagree (1, 5.6%),

Slightly Disagree (0, 0.0%),

Neither Agree nor Disagree (1, 5.6%),

Slightly Agree (0, 0.0%),

Agree (8, 44.4%),

Strongly Agree (4, 22.2%),

Very Strongly Agree

(3, 16.7%),

I Don't Know (0, 0.0%)

Median Score 7

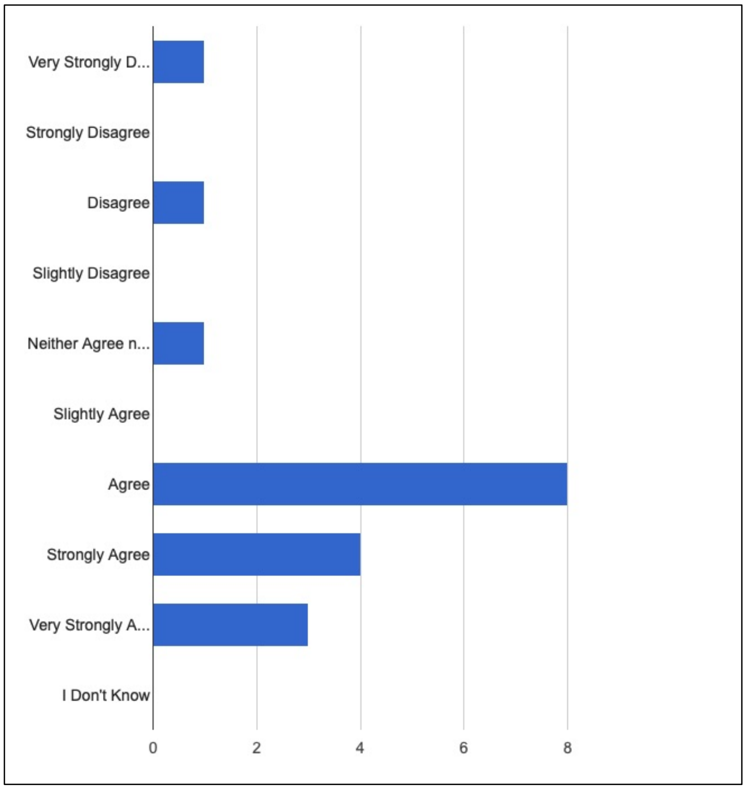

18. When caring for LTC residents, it is challenging to balance the management of several diseases (each of which often requires several medications) with the expectation in LTC to reduce the total number of medications.

Very Strongly Disagree (0, 0.0%),

Strongly Disagree (0, 0.0%),

Disagree (1, 5.6%), S

lightly Disagree (3, 16.7%),

Neither Agree nor Disagree (2, 11.1%),

Slightly Agree (1, 5.6%),

Agree (5, 27.8%),

Strongly Agree (3, 16.7%),

Very Strongly Agree

(1, 5.6%),

I Don't Know (2, 11.1%)

Median Score 7

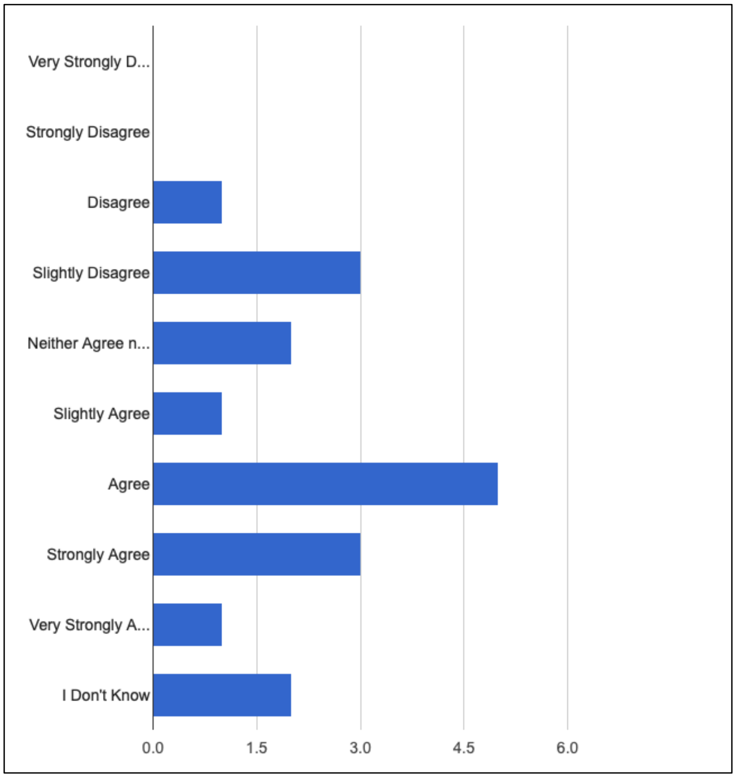

19. Having access to specialist physician advice via phone can improve care.

Very Strongly Disagree (0, 0.0%),

Strongly Disagree (0, 0.0%),

Disagree (0, 0.0%),

Slightly Disagree (0, 0.0%),

Neither Agree nor Disagree (1, 5.6%),

Slightly Agree (1, 5.6%),

Agree (8, 44.4%),

Strongly Agree (3, 16.7%),

Very Strongly Agree

(5, 27.8%),

I Don't Know (0, 0.0%)

Median Score 7

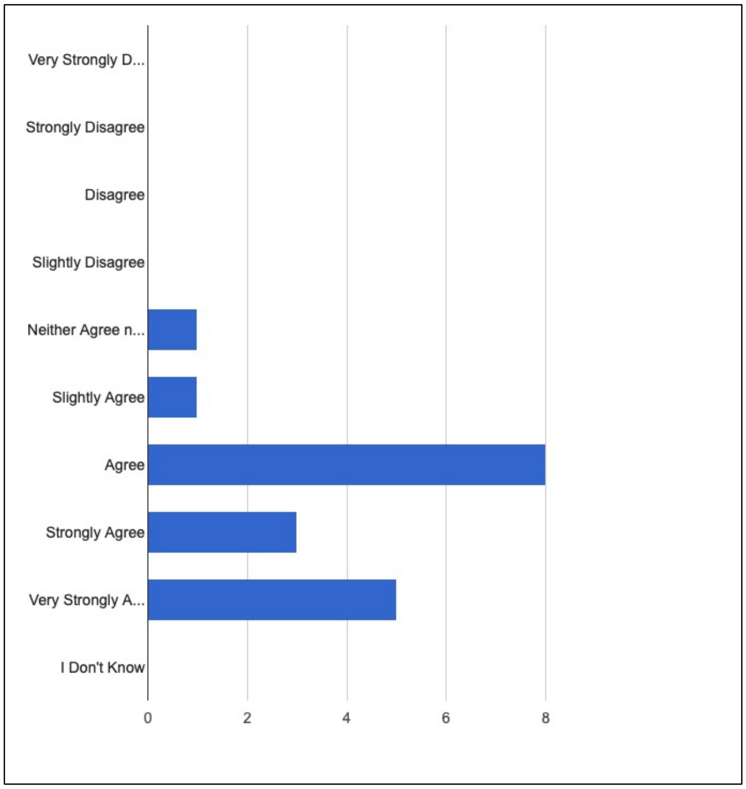

20. Medication choices within LTC should focus more on pain and symptom management, and less on longer- term preventative care (e.g. lipid lowering; tight blood glucose control; blood pressure management).

Very Strongly Disagree (0, 0.0%),

Strongly Disagree (0, 0.0%),

Disagree (1, 5.6%),

Slightly Disagree (0, 0.0%),

Neither Agree nor Disagree (0, 0.0%),

Slightly Agree (0, 0.0%),

Agree (6, 33.3%),

Strongly Agree (6, 33.3%),

Very Strongly Agree

(4, 22.2%),

I Don't Know (1, 5.6%)

Median Score 8

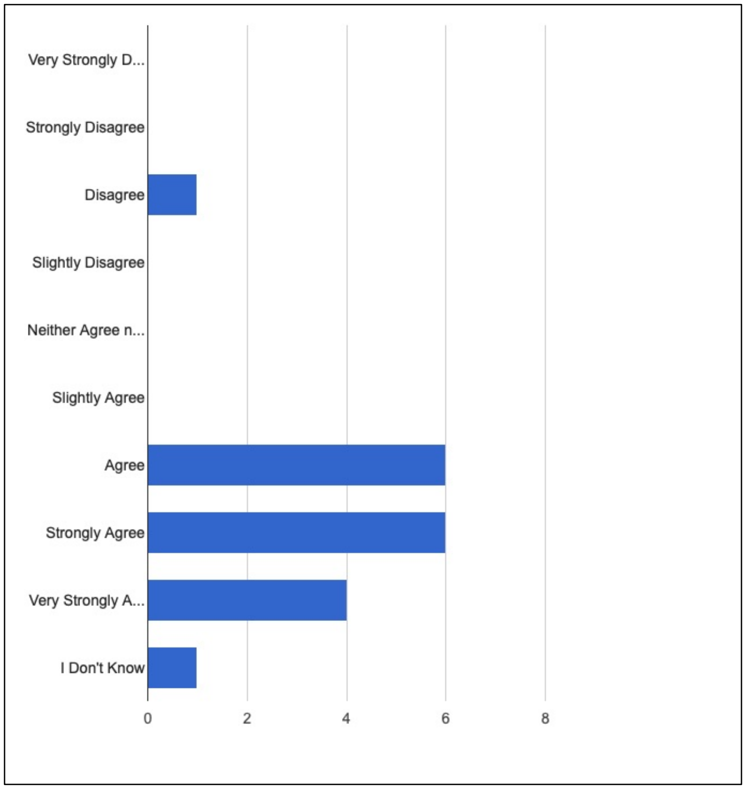

21. Considering the short and long-term impact of medication on functional ability helps LTC physicians choose the appropriate types and numbers of medications.

Very Strongly Disagree (0, 0.0%),

Strongly Disagree (0, 0.0%),

Disagree (0, 0.0%),

Slightly Disagree (0, 0.0%),

Neither Agree nor Disagree (1, 5.6%),

Slightly Agree (1, 5.6%),

Agree (8, 44.4%),

Strongly Agree (4, 22.2%),

Very Strongly Agree

(4, 22.2%),

I Don't Know (0, 0.0%)

Median Score 7

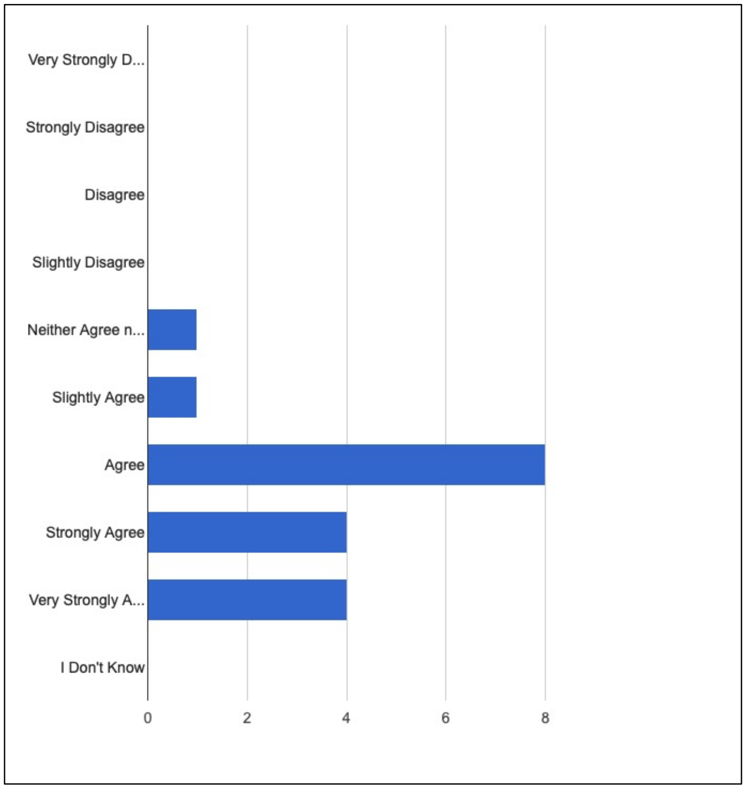

22. Family can be engaged in helping to deliver non- medication (i.e. behavioural) interventions for residents in LTC

Very Strongly Disagree (0, 0.0%),

Strongly Disagree (0, 0.0%),

Disagree (0, 0.0%),

Slightly Disagree (0, 0.0%),

Neither Agree nor Disagree (1, 5.6%),

Slightly Agree (1, 5.6%),

Agree (7, 38.9%),

Strongly Agree (1, 5.6%),

Very Strongly Agree

(8, 44.4%),

I Don't Know (0, 0.0%)

Median Score 7.5

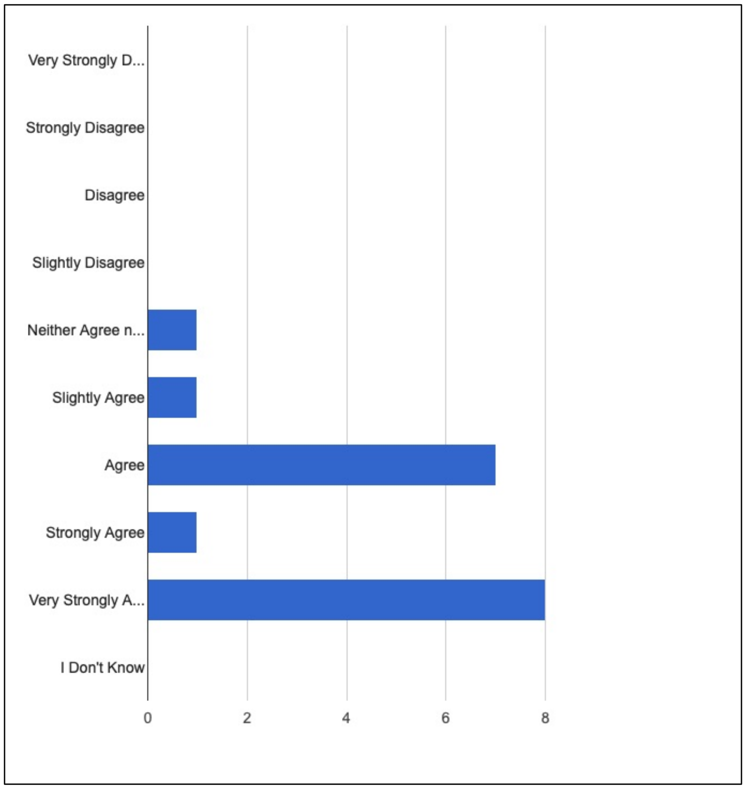

23. Having end of life orders sets can help guide supportive care decision-making once the patient is identified as needing palliative care.

Very Strongly Disagree (0, 0.0%),

Strongly Disagree (0, 0.0%),

Disagree (1, 5.6%),

Slightly Disagree (0, 0.0%),

Neither Agree nor Disagree (0, 0.0%),

Slightly Agree (1, 5.6%),

Agree (5, 27.8%),

Strongly Agree (3, 16.7%),

Very Strongly Agree

(6, 33.3%),

I Don't Know (2, 11.1%)

Median Score 8

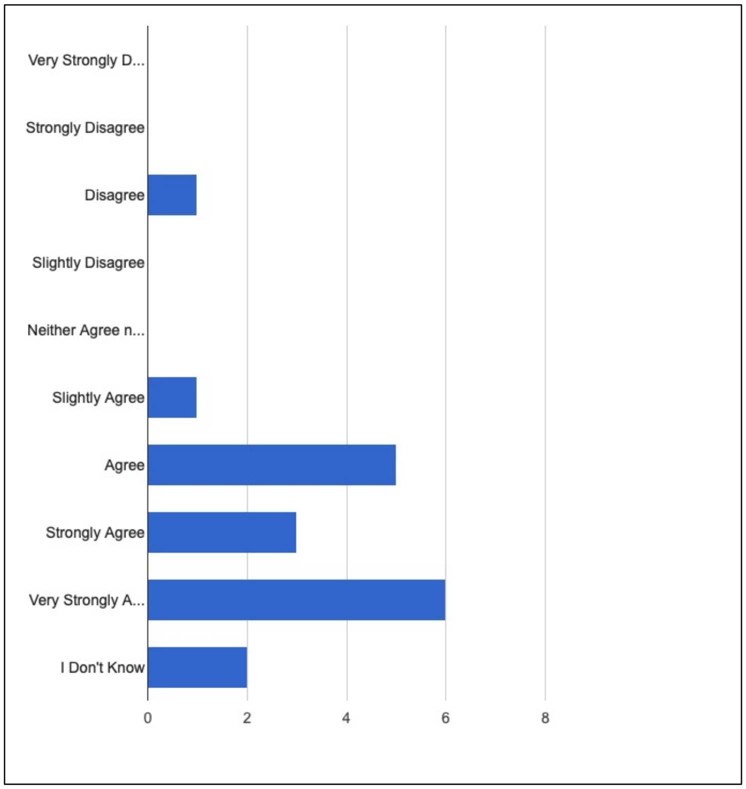

24. Rather than (or in addition to) treating their symptoms with medication, providing space for residents to move and express themselves can help manage stress and anxiety.

Very Strongly Disagree (0, 0.0%),

Strongly Disagree (0, 0.0%),

Disagree (0, 0.0%),

Slightly Disagree (0, 0.0%),

Neither Agree nor Disagree (1, 5.6%),

Slightly Agree (1, 5.6%),

Agree (7, 38.9%),

Strongly Agree (6, 33.3%),

Very Strongly Agree

(3, 16.7%),

I Don't Know (0, 0.0%)

Median Score 7.5

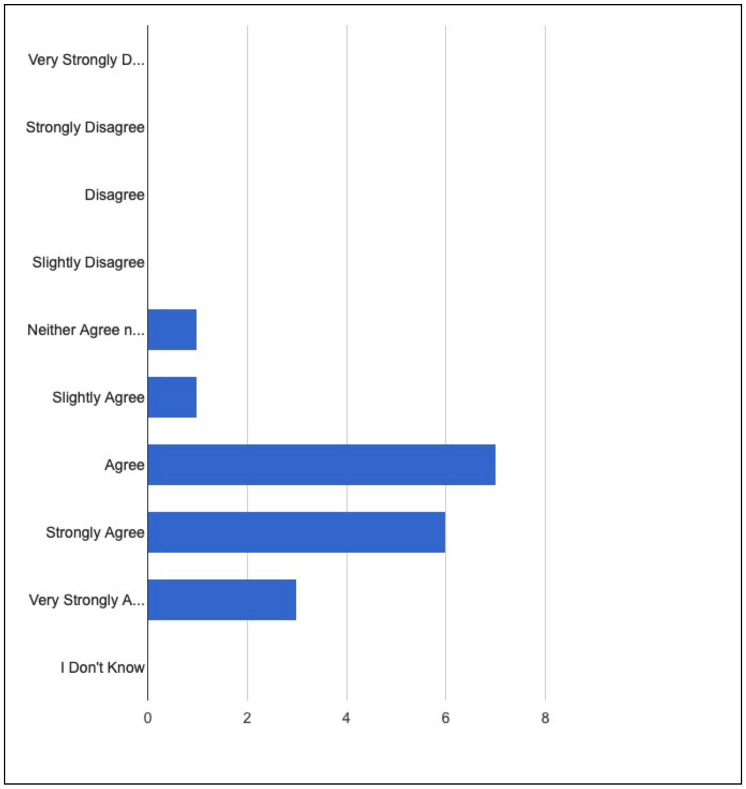

25. The CAM (Confusion Assessment Method) is useful for detecting delirium in LTC residents with dementia.

Very Strongly Disagree (0, 0.0%),

Strongly Disagree (0, 0.0%),

Disagree (0, 0.0%), S

lightly Disagree (1, 5.6%),

Neither Agree nor Disagree (0, 0.0%),

Slightly Agree (1, 5.6%),

Agree (5, 27.8%),

Strongly Agree (4, 22.2%),

Very Strongly Agree

(3, 16.7%),

I Don't Know (4, 22.2%)

Median Score 7.5

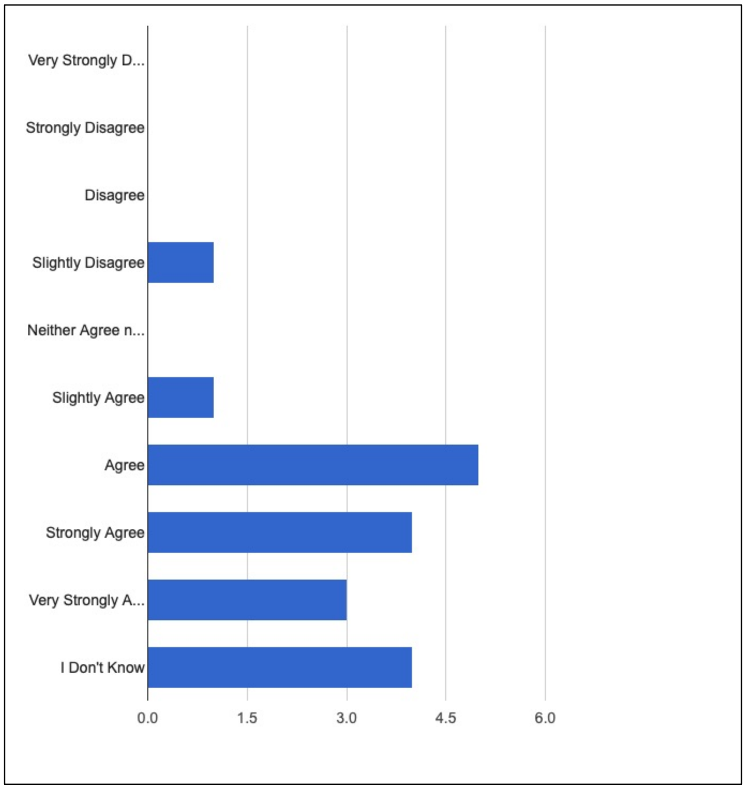

26. Among persons with dementia, it is important to identify the underlying factors associated with a patient'sresponsivebehaviors(e.g. aggression,calling out).

Very Strongly Disagree (0, 0.0%),

Strongly Disagree (0, 0.0%),

Disagree (0, 0.0%),

Slightly Disagree (0, 0.0%),

Neither Agree nor Disagree (0, 0.0%),

Slightly Agree (0, 0.0%),

Agree (3, 16.7%),

Strongly Agree (6, 33.3%),

Very Strongly Agree

(9, 50.0%),

I Don't Know (0, 0.0%)

Median Score 8.5

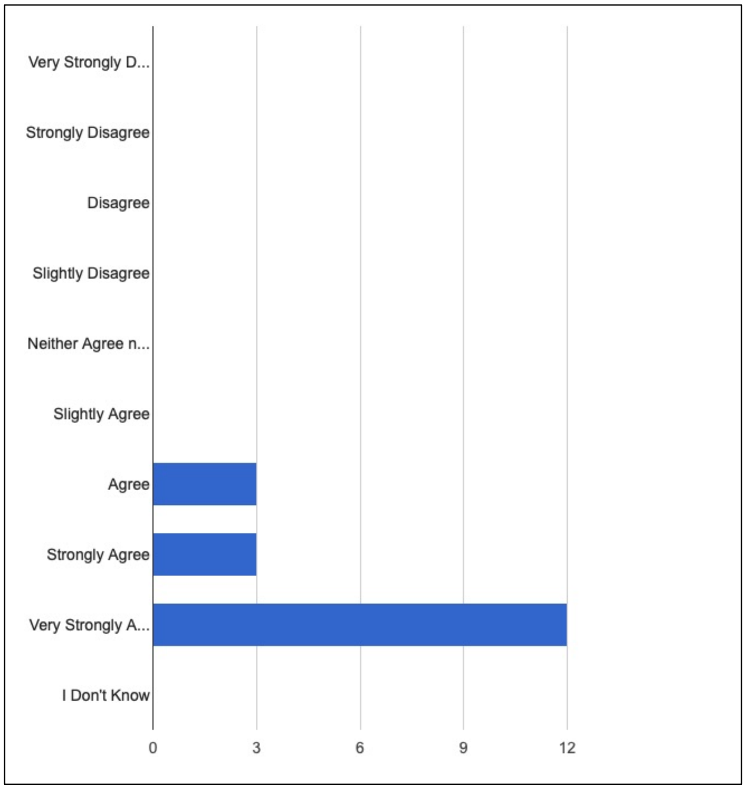

27. Among persons with dementia, it is important to include the family when trying to understand a patient's responsive behaviours.

Very Strongly Disagree (0, 0.0%),

Strongly Disagree (0, 0.0%),

Disagree (0, 0.0%),

Slightly Disagree (0, 0.0%),

Neither Agree nor Disagree (0, 0.0%),

Slightly Agree (0, 0.0%),

Agree (3, 16.7%),

Strongly Agree (3, 16.7%),

Very Strongly Agree

(12, 66.7%),

I Don't Know (0, 0.0%)

Median Score 9

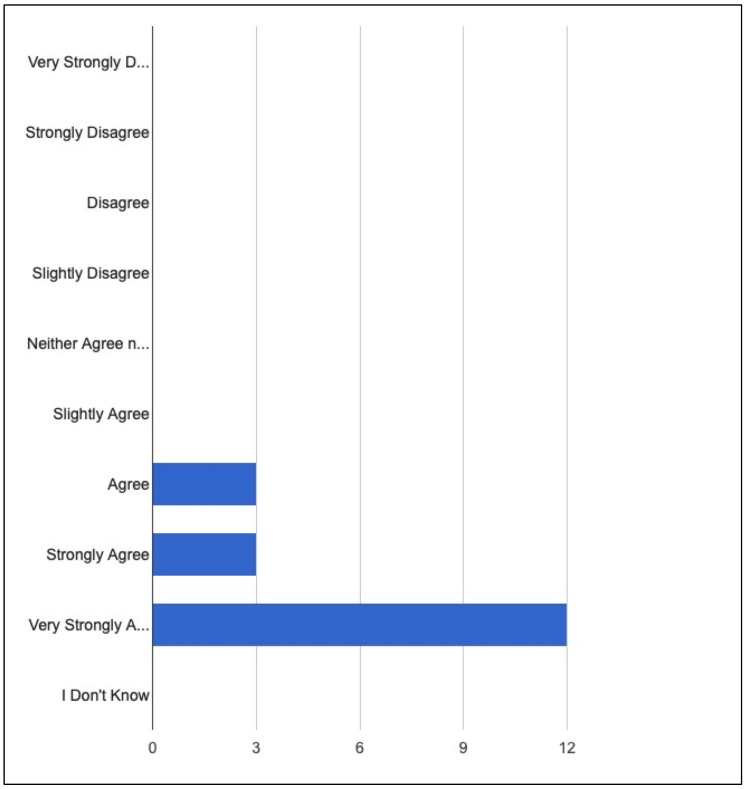

28. Inconsistencies in how different LTC facilities chart symptoms can make it challenging for physicians and other healthcare providers that work within multiple different LTC facilities to care forresidents.

Very Strongly Disagree (0, 0.0%),

Strongly Disagree (0, 0.0%),

Disagree (0, 0.0%),

Slightly Disagree (0, 0.0%),

Neither Agree nor Disagree (0, 0.0%),

Slightly Agree (1, 5.6%),

Agree (5, 27.8%),

Strongly Agree (6, 33.3%), V

ery Strongly Agree

(4, 22.2%),

I Don't Know (2, 11.1%)

Median Score 8

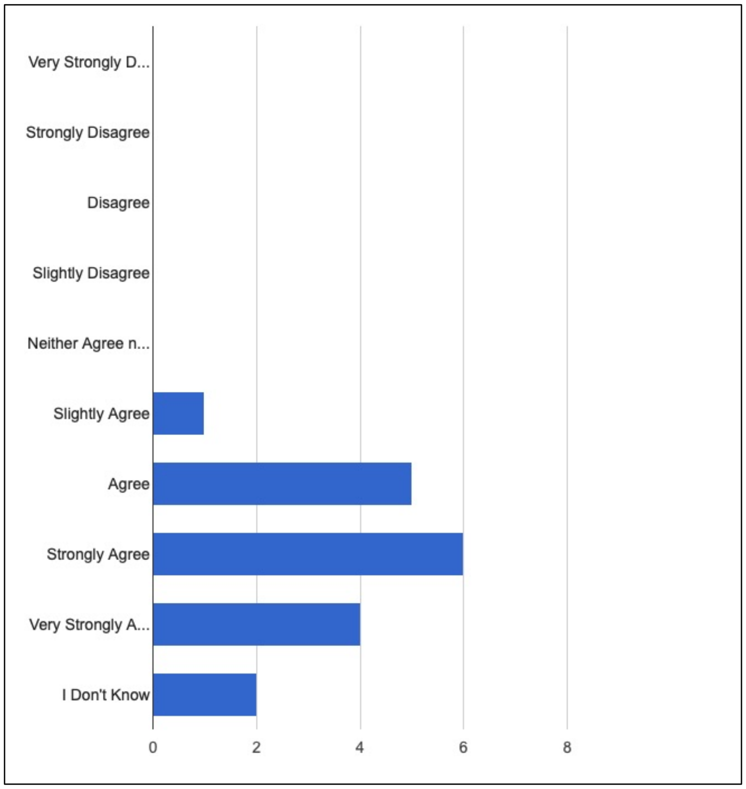

29. Inconsistencies in how different LTC facilities chart symptoms can make it challenging for physicians and other healthcare providers that work within multiple different LTC facilities to communicate with other team members.

Very Strongly Disagree (0, 0.0%),

Strongly Disagree (0, 0.0%),

Disagree (0, 0.0%),

Slightly Disagree (1, 5.6%),

Neither Agree nor Disagree (0, 0.0%),

Slightly Agree (2, 11.1%),

Agree (4, 22.2%),

Strongly Agree (5, 27.8%),

Very Strongly

Agree (4, 22.2%),

I Don't Know (2, 11.1%)

Median Score 8

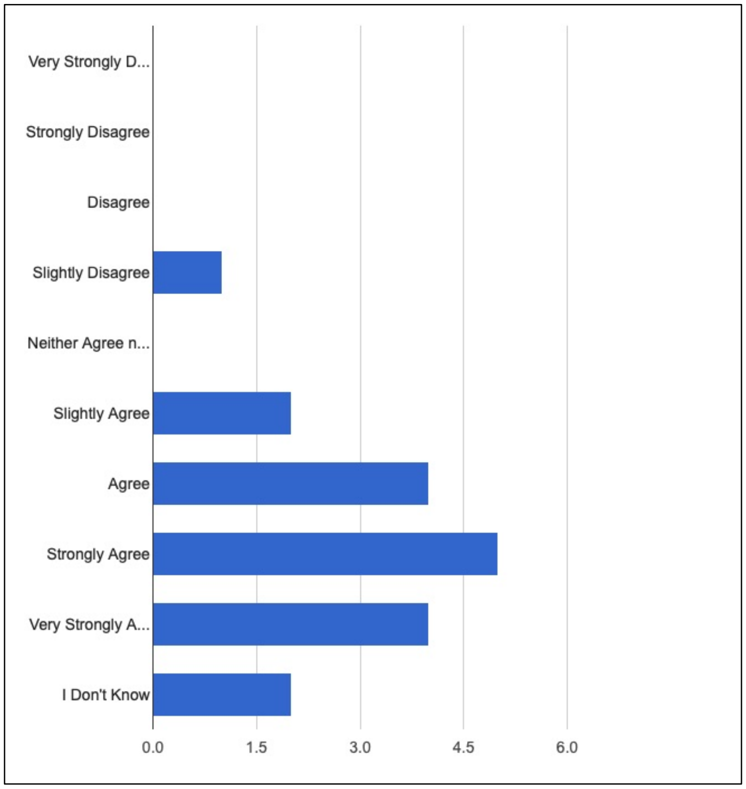

Managing End of Life (11 Statements)

31.It can be challenging to determine where a frail resident is on the life-expectancy trajectory (or curve), making it hard to know when to transition from interventional medical care to palliativecare.

Very Strongly Disagree (0, 0.0%),

Strongly Disagree (0, 0.0%),

Disagree (3, 16.7%),

Slightly Disagree (0, 0.0%),

Neither Agree nor Disagree (1, 5.6%),

Slightly Agree (3, 16.7%),

Agree (7, 38.9%),

Strongly Agree (2, 11.1%),

Very Strongly

Agree (1, 5.6%),

I Don't Know (1, 5.6%)

Median Score 7

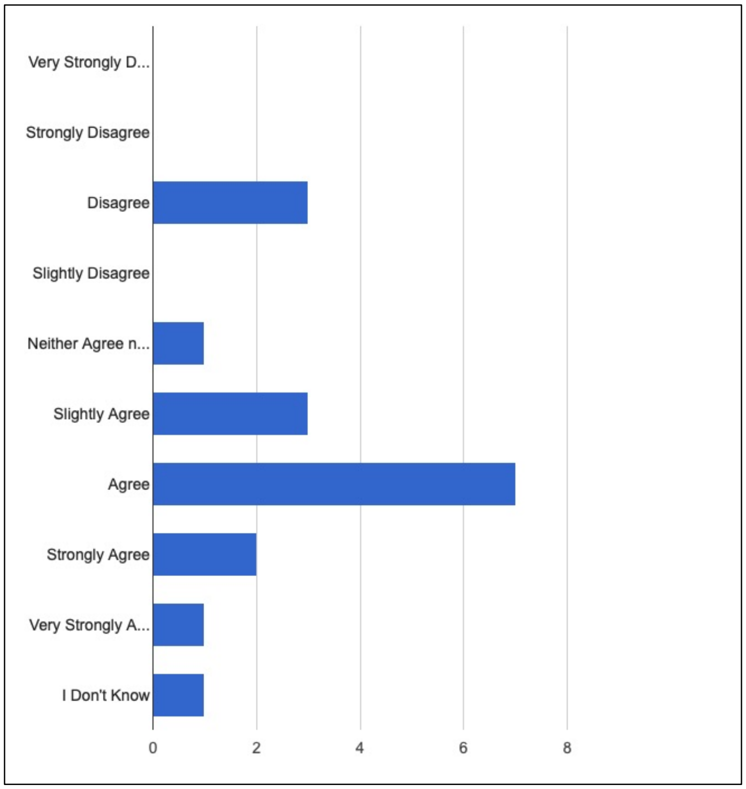

32. Having a standardized frailty assessment tool that is used on a routine basis can help judge a patient's health status from LTC admission up until end of life.

Very Strongly Disagree (0, 0.0%),

Strongly Disagree (0, 0.0%),

Disagree (0, 0.0%),

Slightly Disagree (0, 0.0%),

Neither Agree nor Disagree (3, 16.7%),

Slightly Agree (1, 5.6%),

Agree (7, 38.9%),

Strongly Agree (3, 16.7%),

Very Strongly Agree

(2, 11.1%),

I Don't Know (2, 11.1%)

Median Score 7

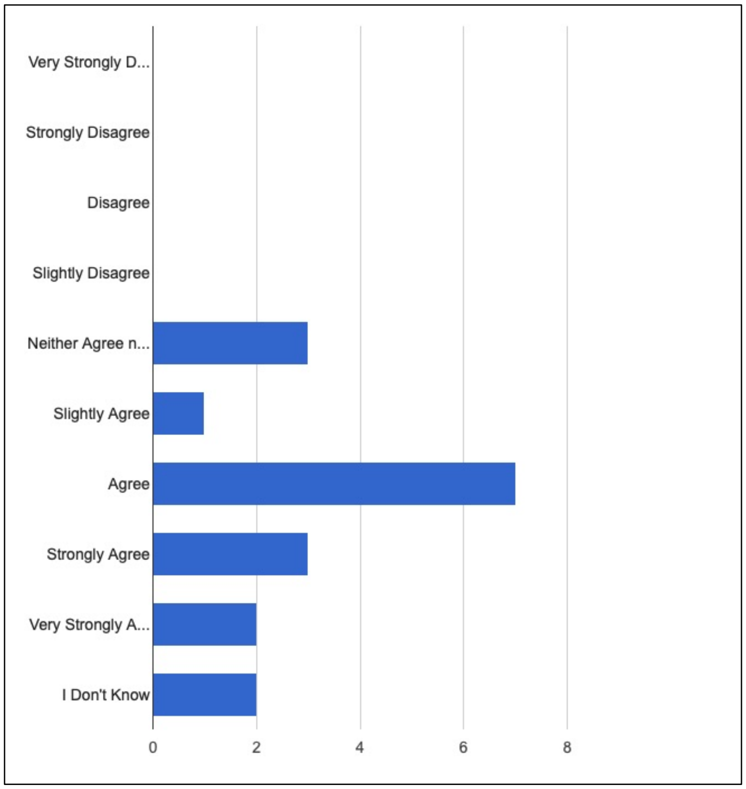

33. The CHESS (Changes in Health, End-Stage Disease, Signs and Symptoms) score can be generated from data already collected within LTC and thus could be helpful in determining when to initiate end of life care if made available for each LTCresident.

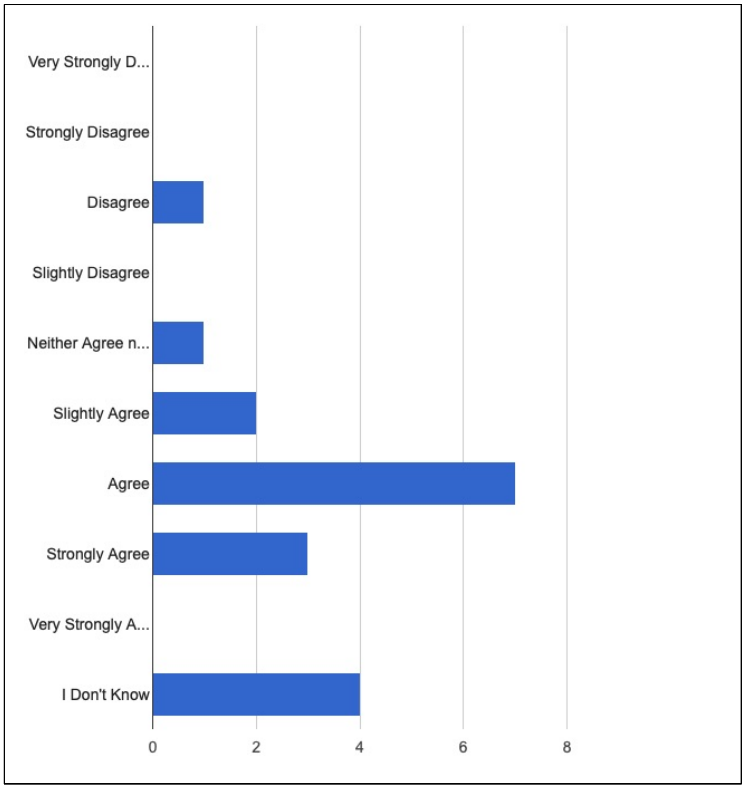

Very Strongly Disagree (0, 0.0%),

Strongly Disagree (0, 0.0%),

Disagree (1, 5.6%),

Slightly Disagree (0, 0.0%),

Neither Agree nor Disagree (1, 5.6%),

Slightly Agree (2, 11.1%),

Agree (7, 38.9%),

Strongly Agree (3, 16.7%),

Very Strongly

Agree (0, 0.0%),

I Don't Know (4, 22.2%)

Median Score 7

34. The care needs of residents increase as they reach the end of their life, yet overall staffing levels remain the same.

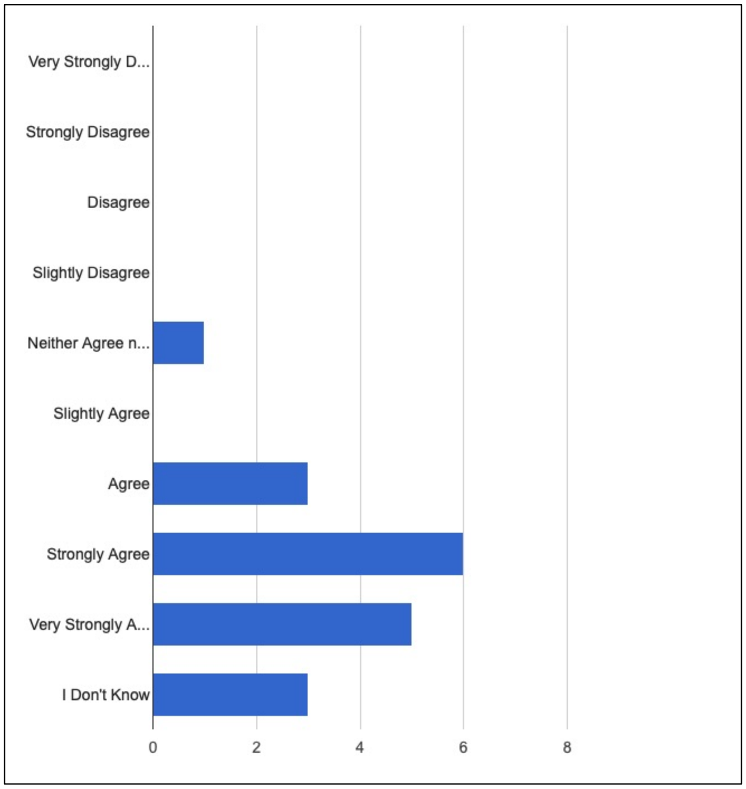

Very Strongly Disagree (0, 0.0%),

Strongly Disagree (0, 0.0%),

Disagree (0, 0.0%),

Slightly Disagree (0, 0.0%),

Neither Agree nor Disagree (1, 5.6%),

Slightly Agree (0, 0.0%),

Agree (3, 16.7%),

Strongly Agree (6, 33.3%),

Very Strongly Agree

(5, 27.8%),

I Don't Know (3, 16.7%)

Median Score 8

35. Timely identification by LTC staff of residents whose condition has deteriorated and/or who appear at high risk of imminent death, and communicating this to the attending LTC physician can improve outcomes at end of life.

Very Strongly Disagree (0, 0.0%),

Strongly Disagree (0, 0.0%),

Disagree (0, 0.0%),

Slightly Disagree (0, 0.0%),

Neither Agree nor Disagree (0, 0.0%),

Slightly Agree (0, 0.0%),

Agree (3, 16.7%),

Strongly Agree (8, 44.4%),

Very Strongly Agree

(7, 38.9%),

I Don't Know (0, 0.0%)

Median Score 8

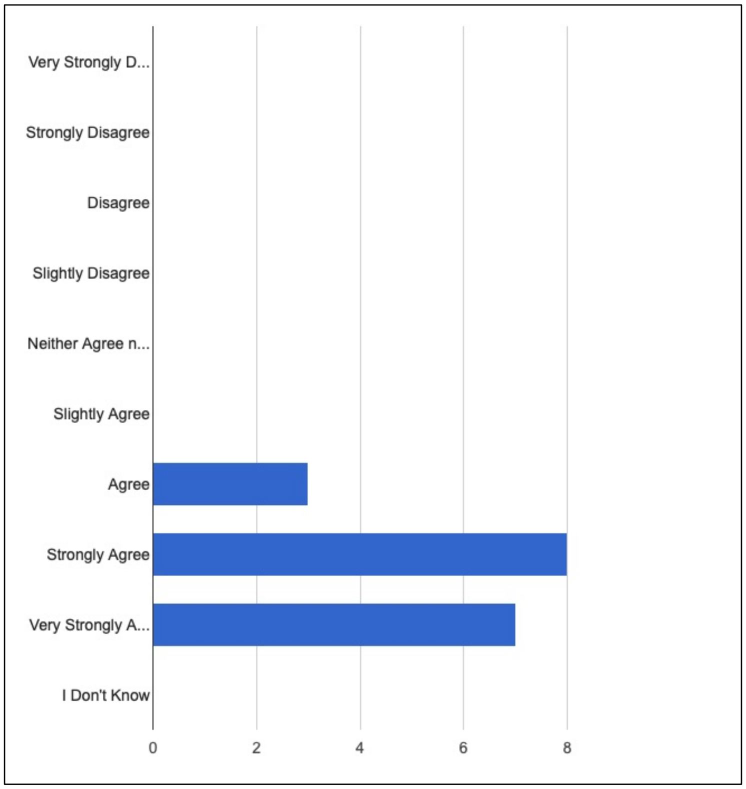

36. It can be difficult to access multidisciplinary support in my community(e.g.psychologists,SW,chaplaincy, chronic pain service).

Very Strongly Disagree (0, 0.0%),

Strongly Disagree (0, 0.0%),

Disagree (1, 5.6%),

Slightly Disagree (2, 11.1%),

Neither Agree nor Disagree (2, 11.1%),

Slightly Agree (2, 11.1%),

Agree (2, 11.1%),

Strongly Agree (3, 16.7%),

Very Strongly

Agree (5, 27.8%),

I Don't Know (1, 5.6%)

Median Score 7

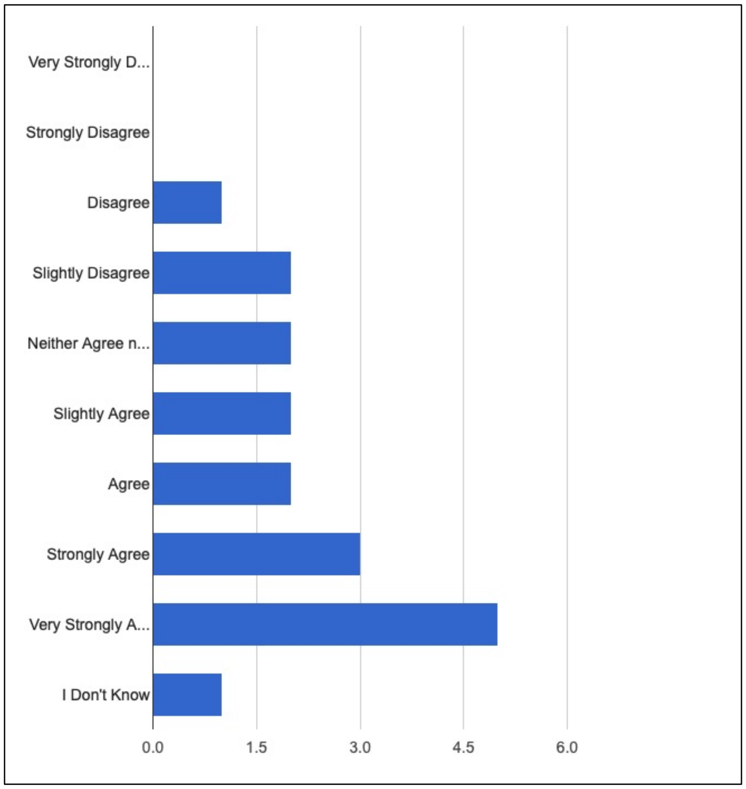

37. Engaging residents and families in discussions about goals of care, treatment preferences and palliative care needs can improve end of life care outcomes.

Very Strongly Disagree (0, 0.0%),

Strongly Disagree (0, 0.0%),

Disagree (0, 0.0%),

Slightly Disagree (0, 0.0%),

Neither Agree nor Disagree (0, 0.0%),

Slightly Agree (0, 0.0%),

Agree (2, 11.1%),

Strongly Agree (5, 27.8%), V

ery Strongly Agree

(11, 61.1%),

I Don't Know (0, 0.0%)

Median Score 9

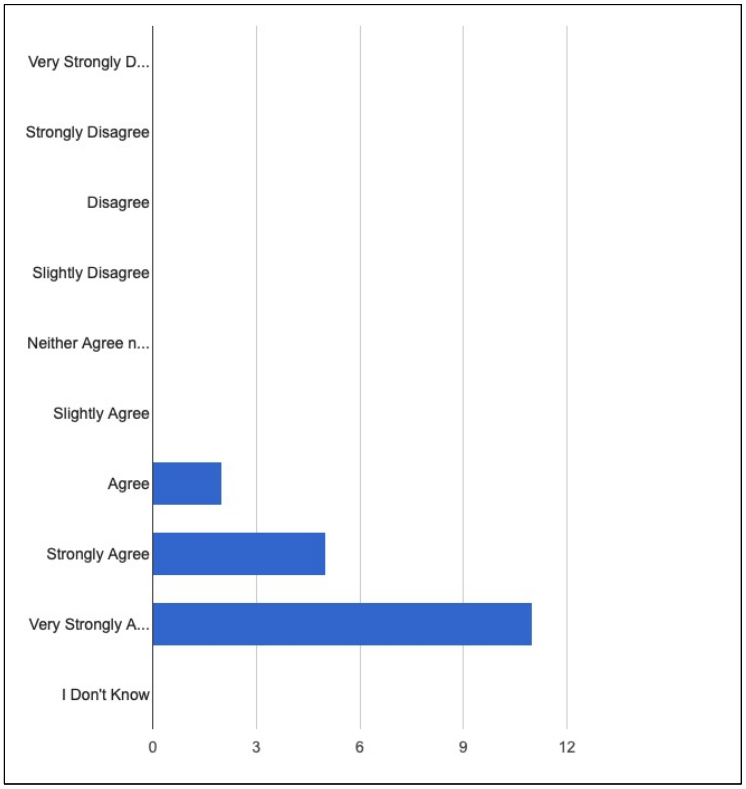

38. A collaborative approach to care improves care by improving communicationbetweenteammembers.

Very Strongly Disagree (0, 0.0%),

Strongly Disagree (0, 0.0%),

Disagree (0, 0.0%),

Slightly Disagree (0, 0.0%),

Neither Agree nor Disagree (0, 0.0%),

Slightly Agree (0, 0.0%),

Agree (4, 22.2%),

Strongly Agree (3, 16.7%),

Very Strongly Agree

(11, 61.1%),

I Don't Know (0, 0.0%)

Median Score 9

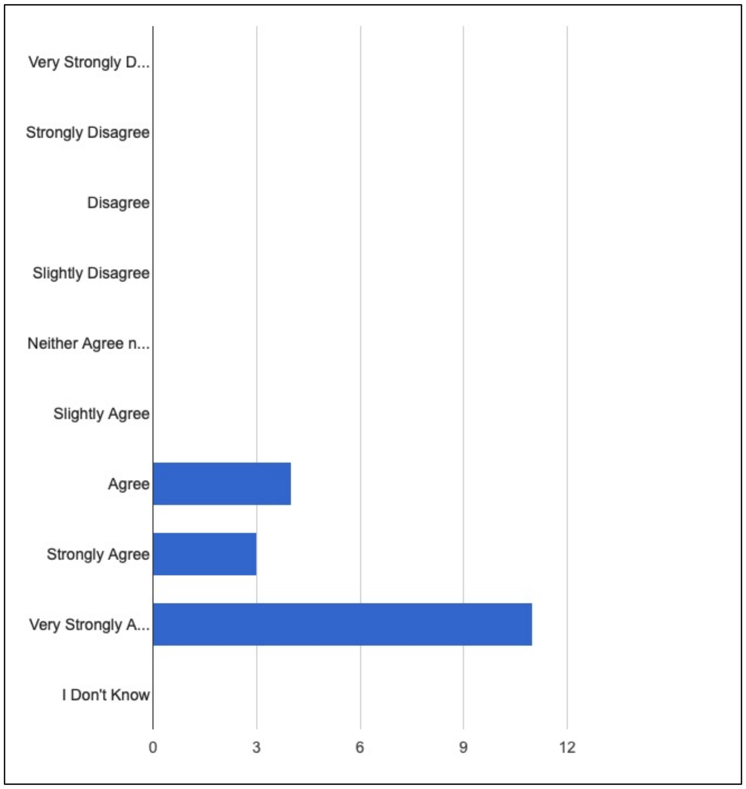

39. A documented palliative care pathway helps to identify changes in patient status, manage family expectations, set staff expectations, and organize the provision of care.

Very Strongly Disagree (0, 0.0%),

Strongly Disagree (0, 0.0%),

Disagree (0, 0.0%),

Slightly Disagree (0, 0.0%),

Neither Agree nor Disagree (0, 0.0%),

Slightly Agree (0, 0.0%),

Agree (5, 27.8%),

Strongly Agree (2, 11.1%),

Very Strongly Agree

(8, 44.4%),

I Don't Know (3, 16.7%)

Median Score 9

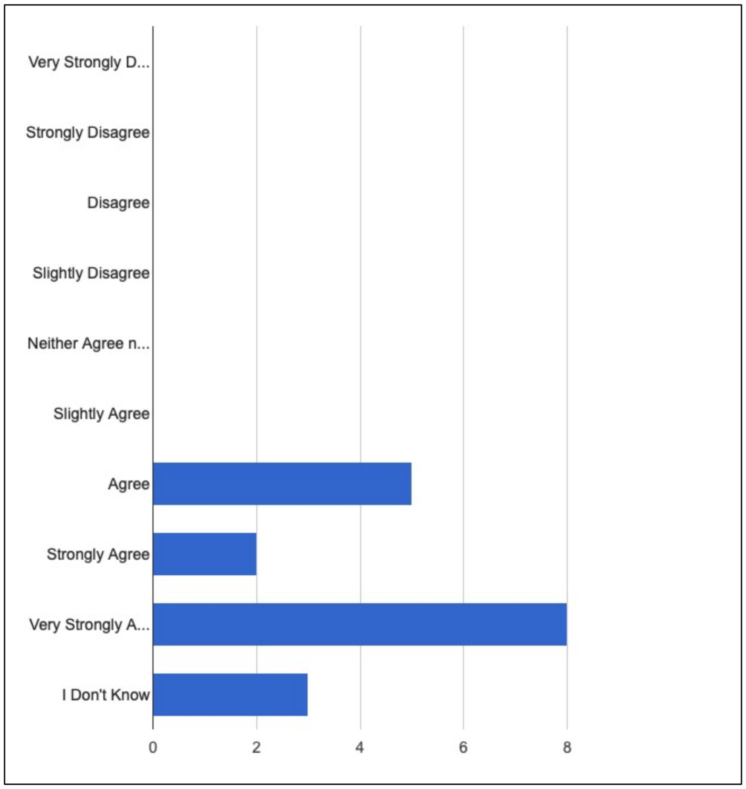

40. It is important to determine and include family expectations in care decision making, and not rely exclusively on standardized care management protocols.

Very Strongly Disagree (0, 0.0%),

Strongly Disagree (0, 0.0%),

Disagree (1, 5.6%),

Slightly Disagree (0, 0.0%),

Neither Agree nor Disagree (0, 0.0%),

Slightly Agree (0, 0.0%),

Agree (4, 22.2%),

Strongly Agree (4, 22.2%),

Very Strongly Agree

(9, 50.0%),

I Don't Know (0, 0.0%)

Median Score 8.5

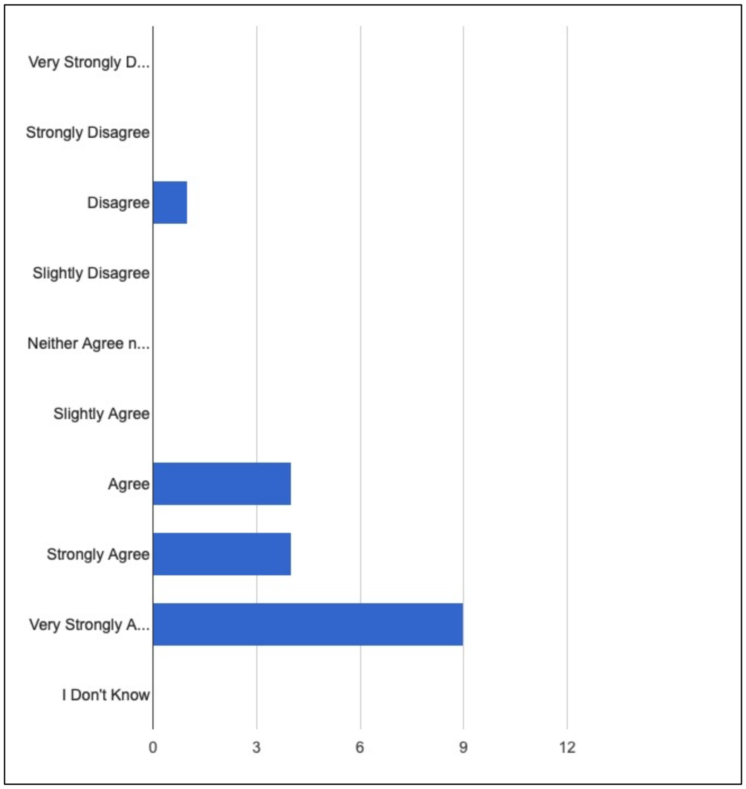

41. It is important to have private space within the LTC facility for the patient and family at or near end of life.

Very Strongly Disagree (0, 0.0%),

Strongly Disagree (0, 0.0%),

Disagree (0, 0.0%),

Slightly Disagree (0, 0.0%),

Neither Agree nor Disagree (0, 0.0%),

Slightly Agree (0, 0.0%),

Agree (5, 27.8%),

Strongly Agree (3, 16.7%),

Very Strongly Agree (10, 55.6%),

I Don't Know (0, 0.0%)

Median Score 9

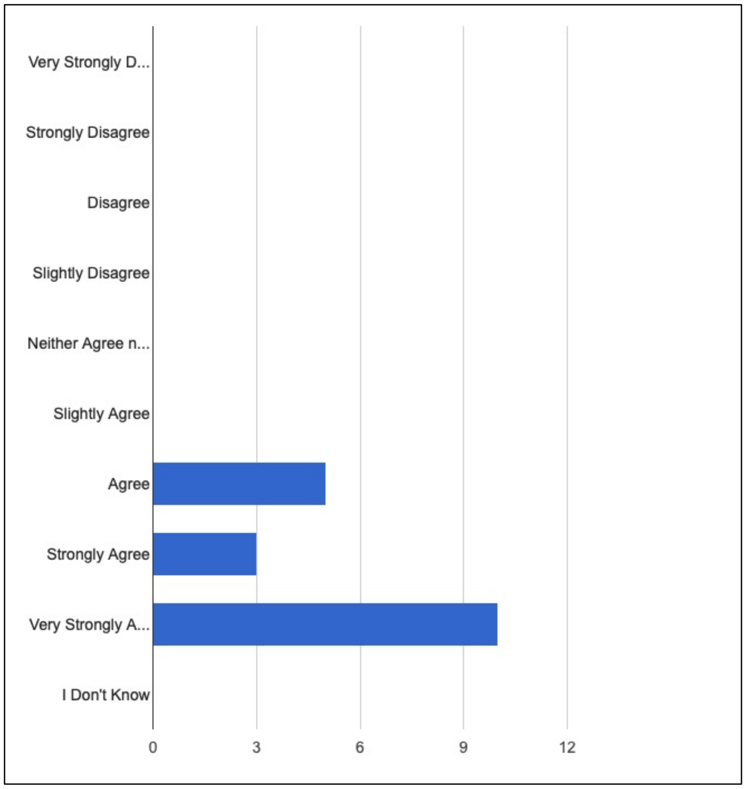

The Context of Providing Supportive End of Life Care within LTC Part I (12 Statements)

43. There is a lack of mental health care supports (e.g. Psychiatrist, Psychologist, SW) within LTC for residents with mental health conditions other than dementia.

Very Strongly Disagree (0, 0.0%),

Strongly Disagree (0, 0.0%),

Disagree (2, 11.1%),

Slightly Disagree (0, 0.0%),

Neither Agree nor Disagree (1, 5.6%),

Slightly Agree (1, 5.6%),

Agree (7, 38.9%),

Strongly Agree (2, 11.1%),

Very Strongly Agree

(4, 22.2%),

I Don't Know (1, 5.6%)

Median Score 7

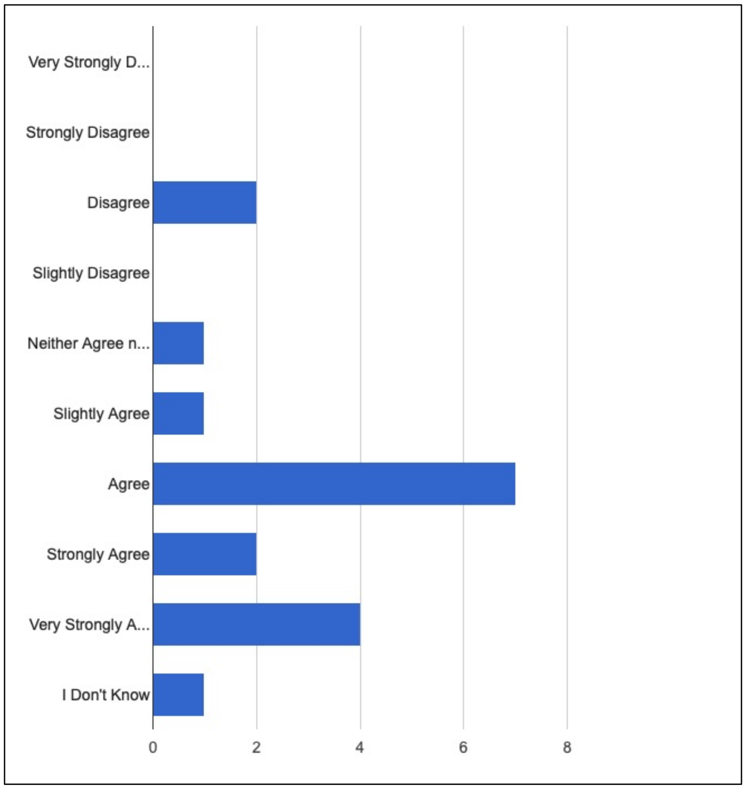

44. Spiritual care providers working in LTC should have an understanding of frailty.

Very Strongly Disagree (0, 0.0%),

Strongly Disagree (0, 0.0%),

Disagree (0, 0.0%),

Slightly Disagree (0, 0.0%),

Neither Agree nor Disagree (2, 11.1%),

Slightly Agree (0, 0.0%),

Agree (9, 50.0%),

Strongly Agree (3, 16.7%),

Very Strongly Agree

(4, 22.2%),

I Don't Know (0, 0.0%)

Median Score 7

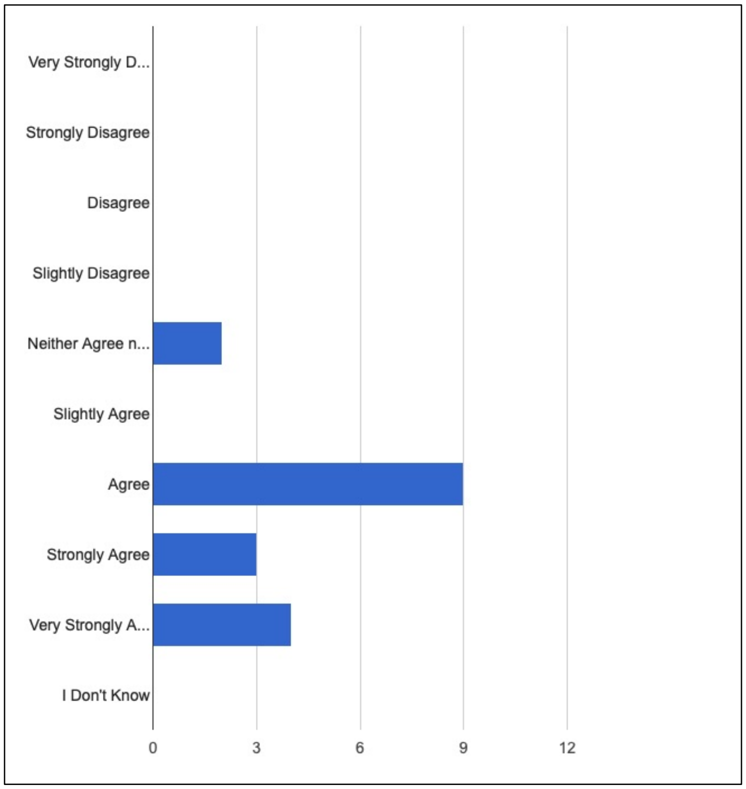

45. Increased access to spiritual care services can improve end of life care outcomes.

Very Strongly Disagree (0, 0.0%),

Strongly Disagree (0, 0.0%),

Disagree (0, 0.0%),

Slightly Disagree (0, 0.0%),

Neither Agree nor Disagree (1, 5.6%),

Slightly Agree (1, 5.6%),

Agree (6, 33.3%),

Strongly Agree (1, 5.6%),

Very Strongly Agree

(7, 38.9%),

I Don't Know (2, 11.1%)

Median Score 7.5

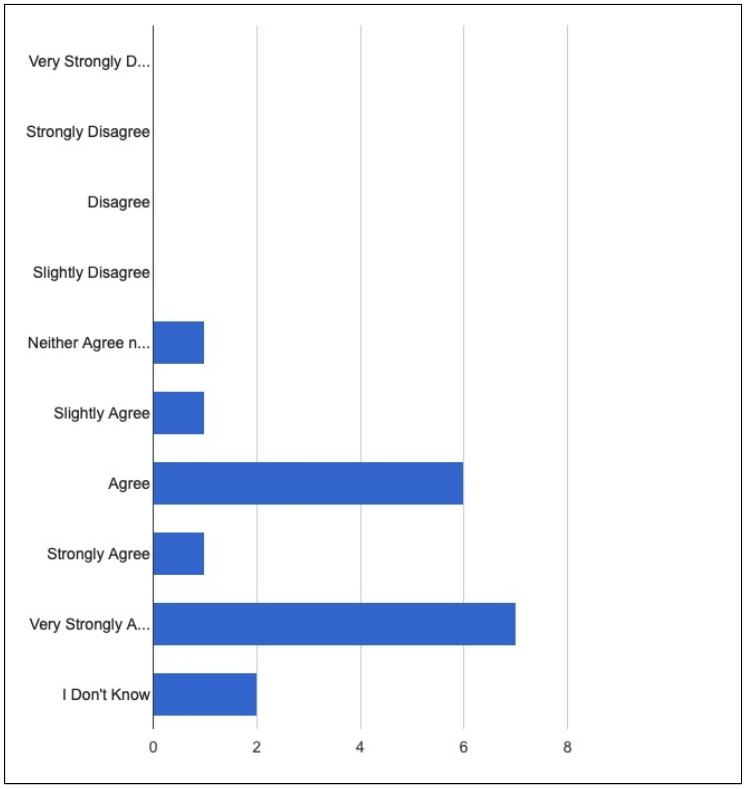

46. LTC residents frequently end up being transferred to acute care facilities as the result of inadequate staff to patient ratios needed to manage the increasing care needs of those nearing the end of life.

Very Strongly Disagree (0, 0.0%),

Strongly Disagree (2, 11.1%),

Disagree (1, 5.6%), Slightly Disagree (1, 5.6%),

Neither Agree nor Disagree (0, 0.0%),

Slightly Agree (0, 0.0%),

Agree (8, 44.4%),

Strongly Agree (2, 11.1%),

Very Strongly Agree

(1, 5.6%),

I Don't Know (3, 16.7%)

Median Score 7

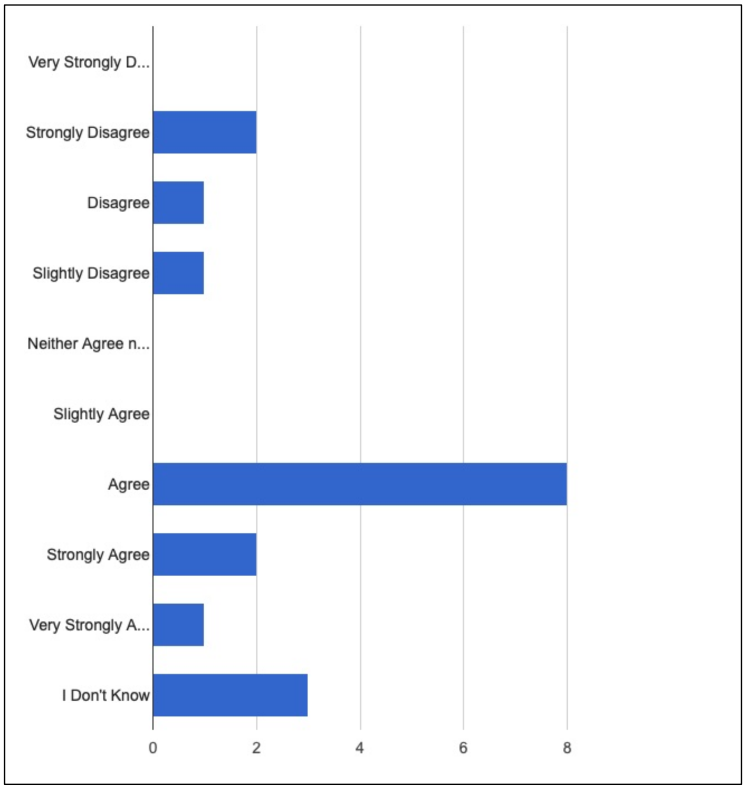

47. LTC staff are uncomfortable with providing supportive end of life care.

Very Strongly Disagree (1, 5.6%),

Strongly Disagree (1, 5.6%),

Disagree (4, 22.2%),

Slightly Disagree (1, 5.6%),

Neither Agree nor Disagree (2, 11.1%),

Slightly Agree (2, 11.1%),

Agree (5, 27.8%),

Strongly Agree (1, 5.6%),

Very Strongly Agree

(0, 0.0%),

I Don't Know (1, 5.6%)

Median Score 5

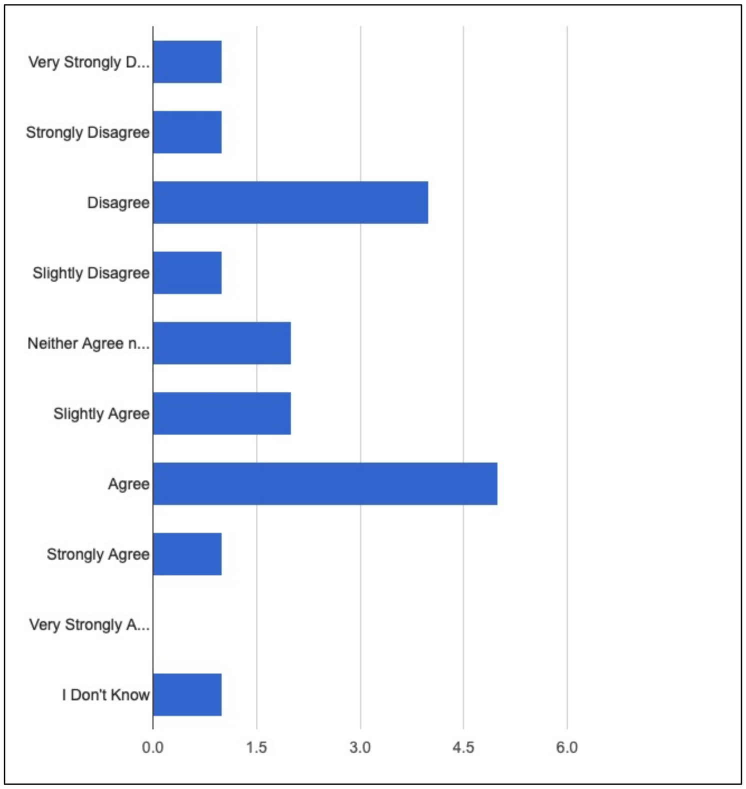

48. LTC staff are unknowledgeable and/or inexperienced in supportive end of life care.

Very Strongly Disagree (1, 5.6%),

Strongly Disagree (1, 5.6%),

Disagree (4, 22.2%),

Slightly Disagree (2, 11.1%),

Neither Agree nor Disagree (1, 5.6%),

Slightly Agree (2, 11.1%),

Agree (4, 22.2%),

Strongly Agree (1, 5.6%),

Very Strongly Agree

(1, 5.6%),

I Don't Know (1, 5.6%)

Median Score 5

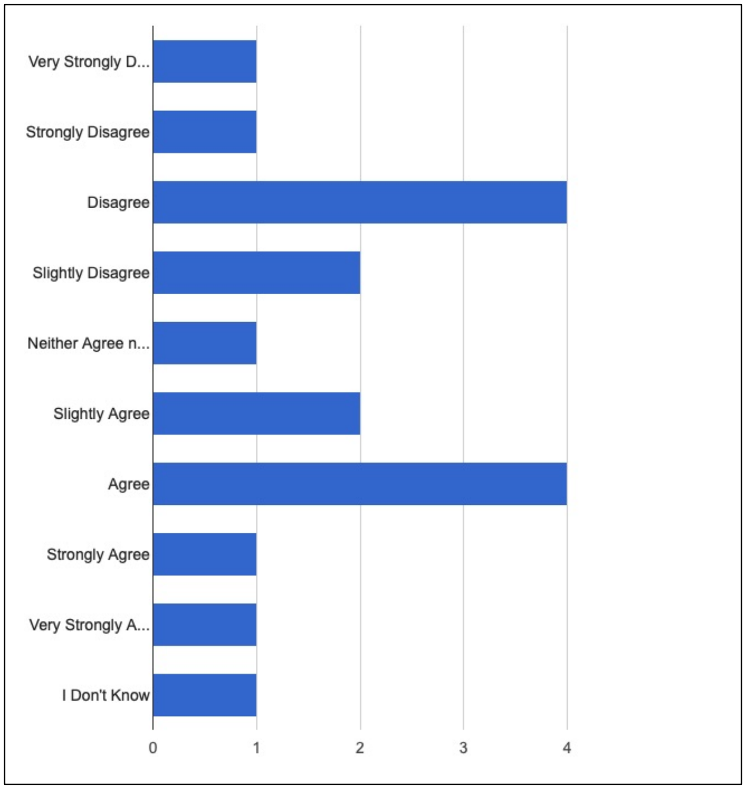

49. Providing LTC staff with education and training about supportive end of life care improves end of life care practices.

Very Strongly Disagree (0, 0.0%),

Strongly Disagree (0, 0.0%),

Disagree (0, 0.0%),

Slightly Disagree (0, 0.0%),

Neither Agree nor Disagree (0, 0.0%),

Slightly Agree (0, 0.0%),

Agree (4, 22.2%),

Strongly Agree (7, 38.9%),

Very Strongly Agree

(7, 38.9%),

I Don't Know (0, 0.0%)

Median Score 8

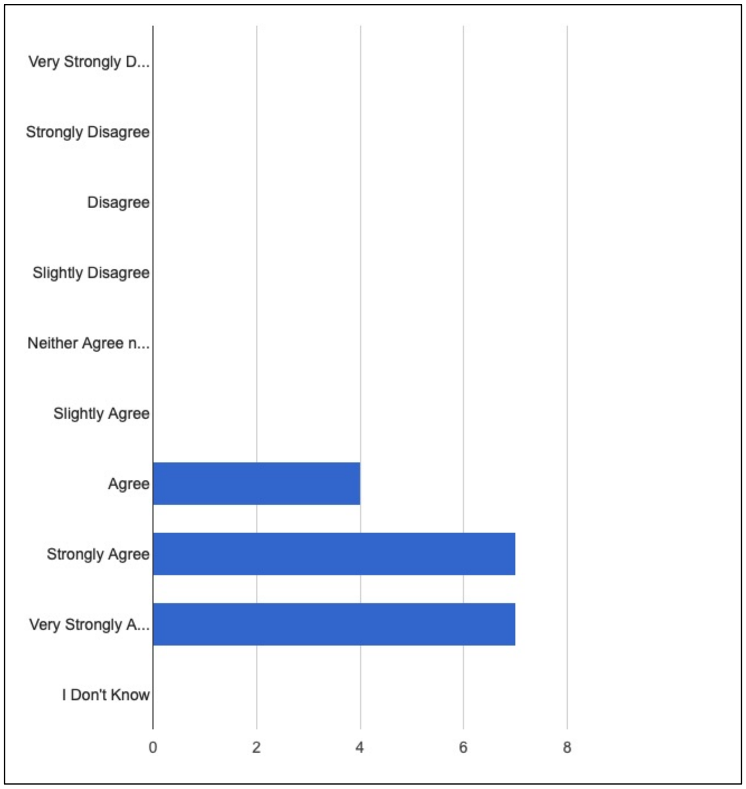

50. Physicians can support LTC staff by encouraging them to attend palliative care education sessions on-line or in person, and advocating to management for the time and money LTC staff need in order to participate.

Very Strongly Disagree (0, 0.0%),

Strongly Disagree (0, 0.0%),

Disagree (0, 0.0%),

Slightly Disagree (0, 0.0%),

Neither Agree nor Disagree (0, 0.0%),

Slightly Agree (0, 0.0%),

Agree (10, 55.6%),

Strongly Agree (1, 5.6%),

Very Strongly Agree

(6, 33.3%),

I Don't Know (1, 5.6%)

Median Score 7

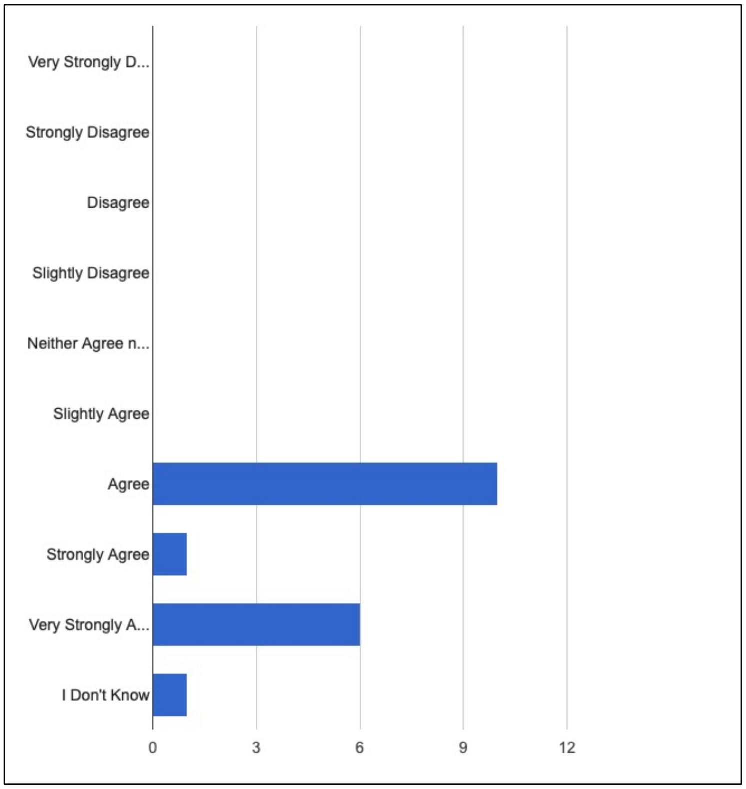

51. The best education for staff initially resistant to providing supportive end of life care in LTC is the practical knowledge they gain from managing symptoms in difficult situations.

Very Strongly Disagree (0, 0.0%),

Strongly Disagree (0, 0.0%),

Disagree (2, 11.1%),

Slightly Disagree (0, 0.0%),

Neither Agree nor Disagree (2, 11.1%),

Slightly Agree (4, 22.2%),

Agree (3, 16.7%),

Strongly Agree (2, 11.1%),

Very Strongly

Agree (2, 11.1%),

I Don't Know (3, 16.7%)

Median Score 6

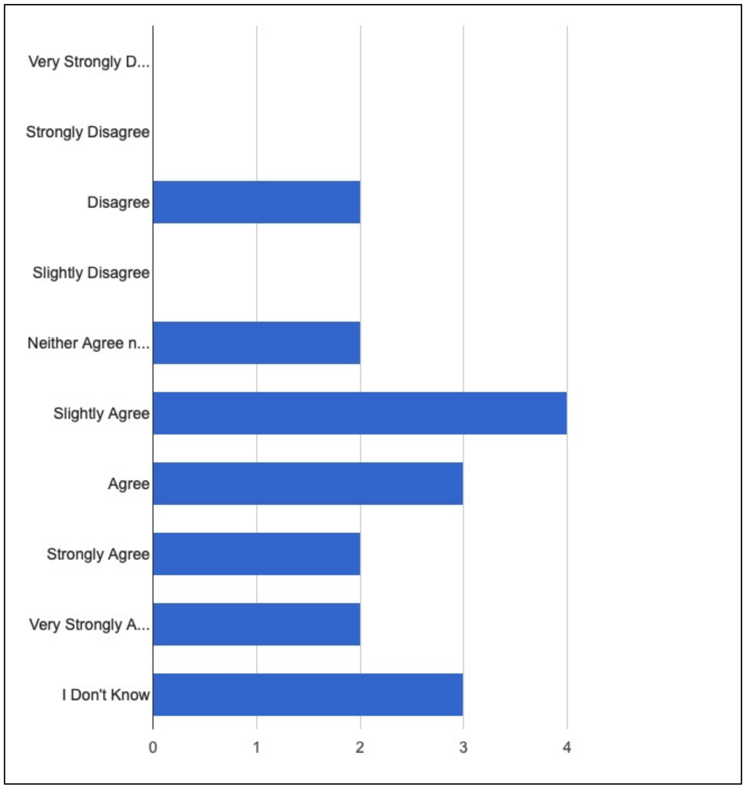

52. Access to palliative care consultants improves the provision of end of life care in LTC.

Very Strongly Disagree (0, 0.0%),

Strongly Disagree (0, 0.0%),

Disagree (0, 0.0%),

Slightly Disagree (0, 0.0%),

Neither Agree nor Disagree (2, 11.1%),

Slightly Agree (0, 0.0%),

Agree (6, 33.3%),

Strongly Agree (4, 22.2%),

Very Strongly Agree

(6, 33.3%),

I Don't Know (0, 0.0%)

Median Score 8

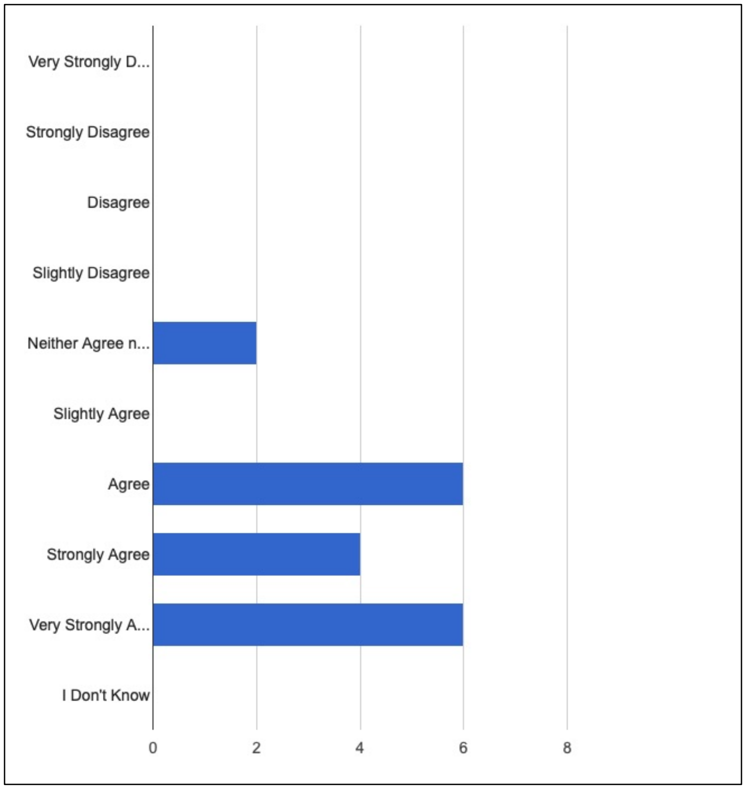

53. By working with palliative care consultants, LTC physicians learn to utilize palliative (supportive) care principles and practices to treat symptoms and care for residents from admission to end-of-life.

Very Strongly Disagree (0, 0.0%),

Strongly Disagree (0, 0.0%),

Disagree (0, 0.0%),

Slightly Disagree (0, 0.0%),

Neither Agree nor Disagree (0, 0.0%),

Slightly Agree (2, 11.1%),

Agree (3, 16.7%),

Strongly Agree (6, 33.3%),

Very Strongly

Agree (6, 33.3%),

I Don't Know (1, 5.6%)

Median Score 8

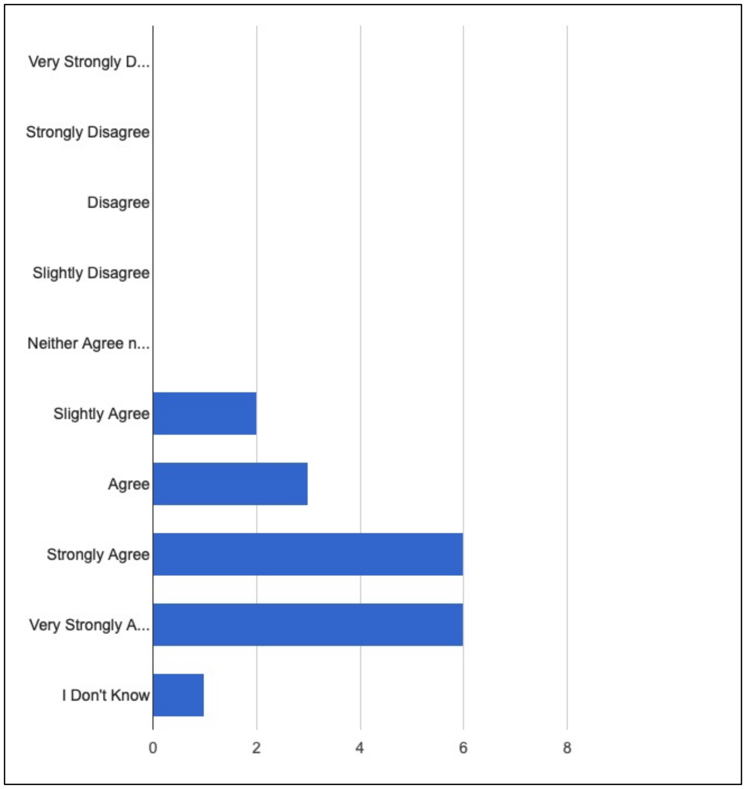

54. It can be difficult to understand and manage the diverse ethnic values and attitudes of LTC staff toward death.

Very Strongly Disagree (0, 0.0%),

Strongly Disagree (0, 0.0%),

Disagree (1, 5.6%),

Slightly Disagree (1, 5.6%),

Neither Agree nor Disagree (0, 0.0%),

Slightly Agree (0, 0.0%),

Agree (10, 55.6%),

Strongly Agree (5, 27.8%),

Very Strongly

Agree (0, 0.0%),

I Don't Know (1, 5.6%)

Median Score 7

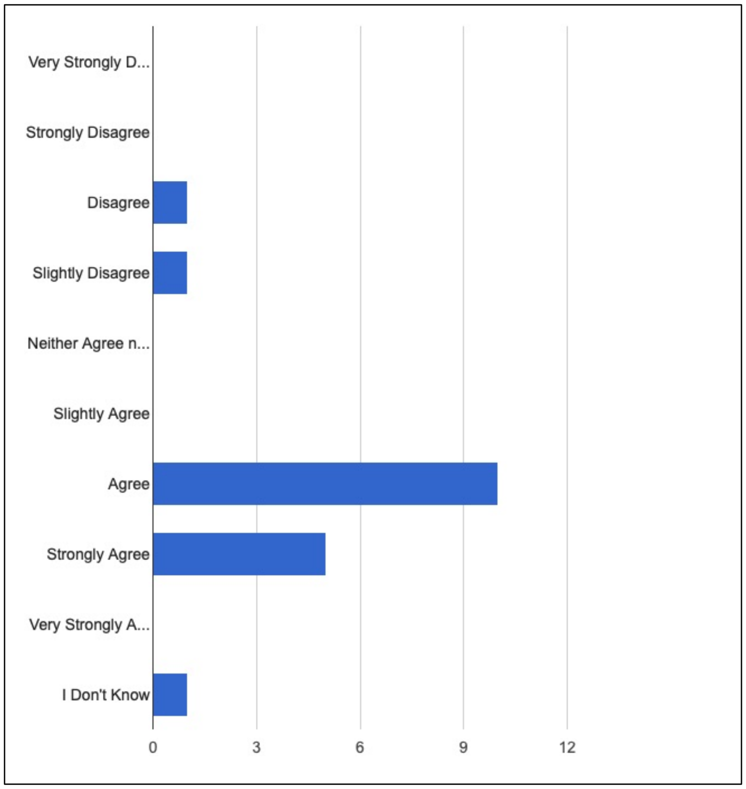

The Context of Providing Supportive End of Life Care within LTC Part II (11 Statements)

56. When they exist within a LTC facility, end of life protocols/pathways are followed inconsistently.

Counts/frequency:

Very Strongly Disagree (0, 0.0%),

Strongly Disagree (0, 0.0%),

Disagree (0, 0.0%),

Slightly Disagree (0, 0.0%),

Neither Agree nor Disagree (2, 11.1%),

Slightly Agree (2, 11.1%),

Agree (8, 44.4%),

Strongly Agree (2, 11.1%),

Very Strongly

Agree (0, 0.0%),

I Don't Know (4, 22.2%)

Median Score 7

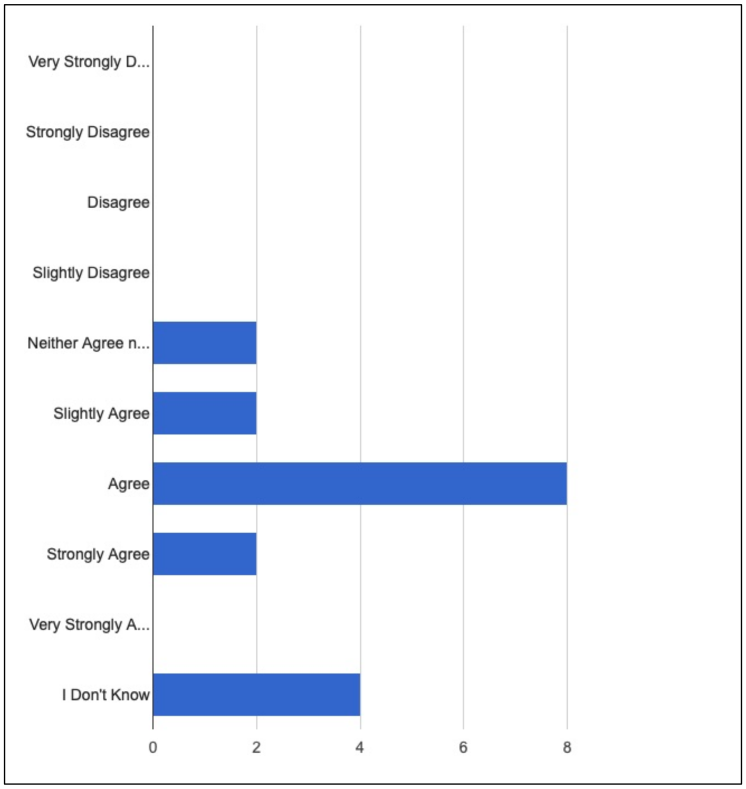

57. There are gaps in physician training and education in terms of the application of supportive end of life care practices within the LTC setting.

Very Strongly Disagree (0, 0.0%),

Strongly Disagree (0, 0.0%),

Disagree (0, 0.0%),

Slightly Disagree (0, 0.0%),

Neither Agree nor Disagree (2, 11.1%),

Slightly Agree (1, 5.6%),

Agree (7, 38.9%),

Strongly Agree (1, 5.6%),

Very Strongly Agree

(4, 22.2%),

I Don't Know (3, 16.7%)

Median Score 7

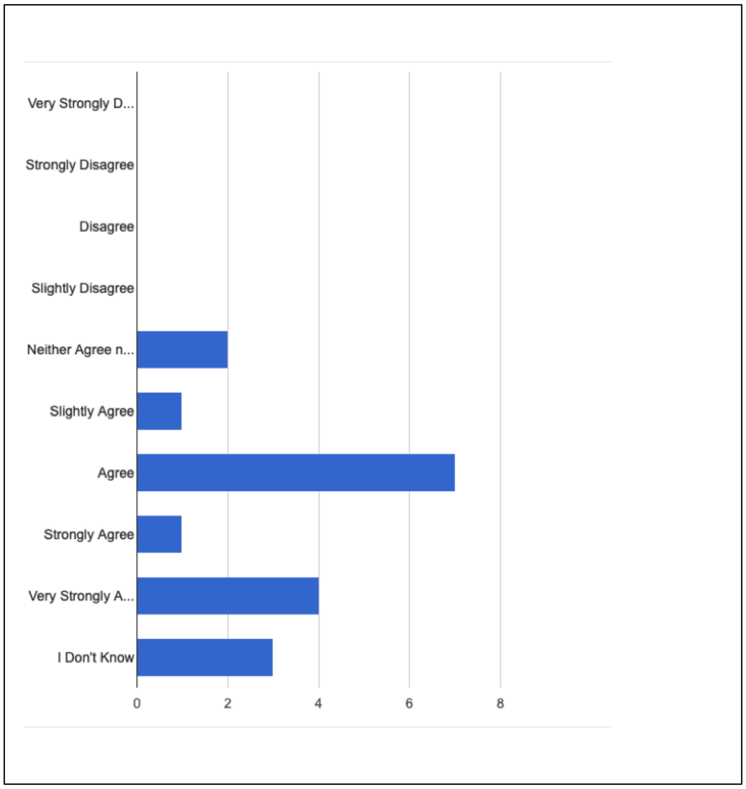

58. There are gaps in physician training and education in terms of the emotional aspects of delivering supportive end of life care in LTC.

Very Strongly Disagree (0, 0.0%),

Strongly Disagree (0, 0.0%),

Disagree (0, 0.0%),

Slightly Disagree (0, 0.0%),

Neither Agree nor Disagree (1, 5.6%),

Slightly Agree (1, 5.6%),

Agree (7, 38.9%),

Strongly Agree (2, 11.1%),

Very Strongly Agree (4, 22.2%),

I Don't Know (3, 16.7%)

Median Score 7

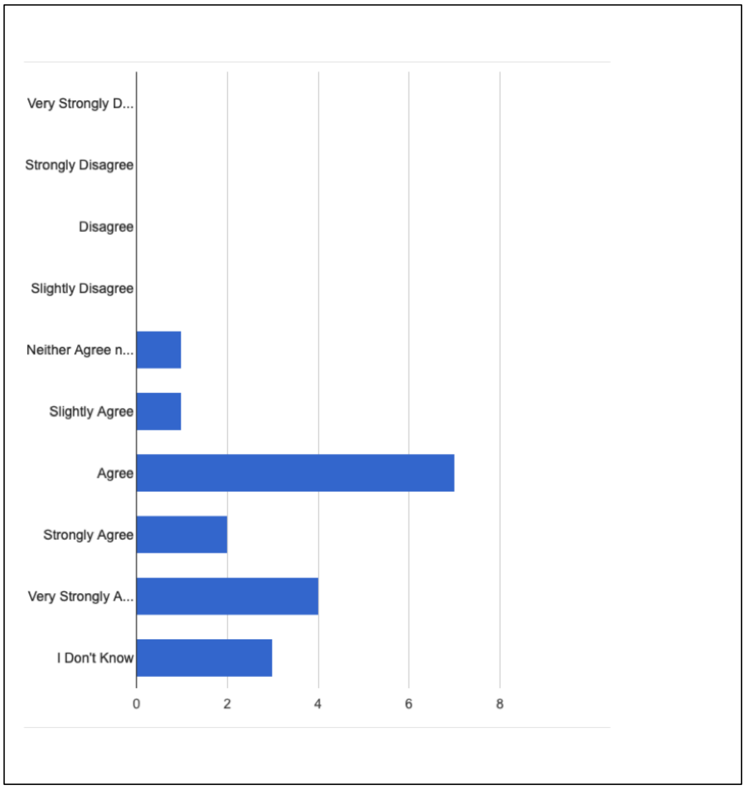

59. LTC physicians often do not consult palliative care early enough in a patient's end of life care path.

Very Strongly Disagree (0, 0.0%),

Strongly Disagree (0, 0.0%),

Disagree (2, 11.1%),

Slightly Disagree (1, 5.6%),

Neither Agree nor Disagree (0, 0.0%),

Slightly Agree (4, 22.2%),

Agree (5, 27.8%),

Strongly Agree (3, 16.7%),

Very Strongly

Agree (1, 5.6%),

I Don't Know (2, 11.1%)

Median Score 7

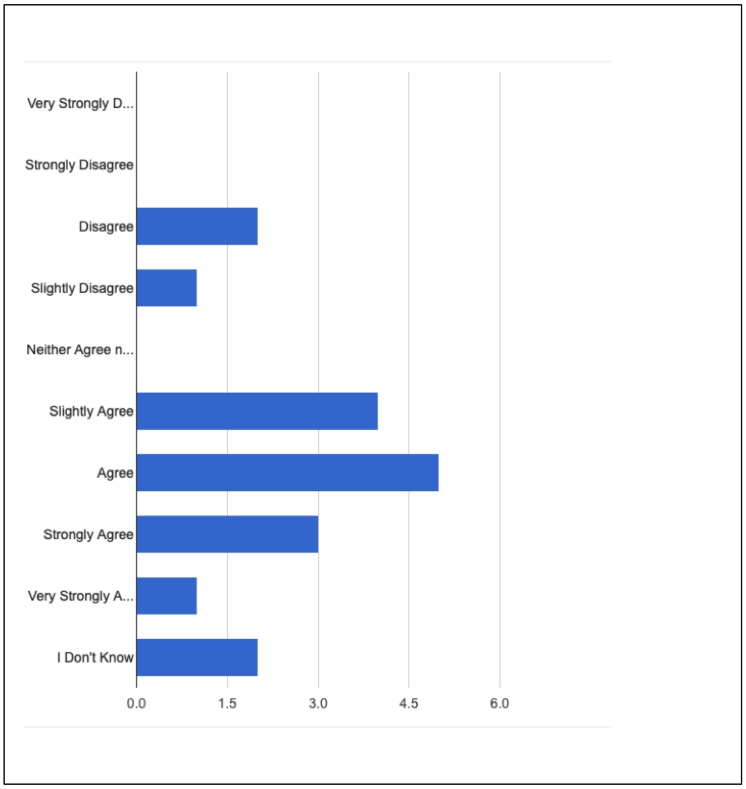

60. LTC physicians' ability to consult with colleagues external to the LTC facility is a facilitator to providing care (e.g. physician colleagues, EMS, Nurse Practitioners, Psychiatrist Services, Geriatric Mental Health Services, Occupational Therapist, Physiotherapist, and Dental services).

Very Strongly Disagree (0, 0.0%),

Strongly Disagree (0, 0.0%),

Disagree (0, 0.0%),

Slightly Disagree (0, 0.0%),

Neither Agree nor Disagree (0, 0.0%),

Slightly Agree (0, 0.0%),

Agree (10, 55.6%),

Strongly Agree (3, 16.7%), V

ery Strongly

Agree (3, 16.7%),

I Don't Know (2, 11.1%)

Median Score 7

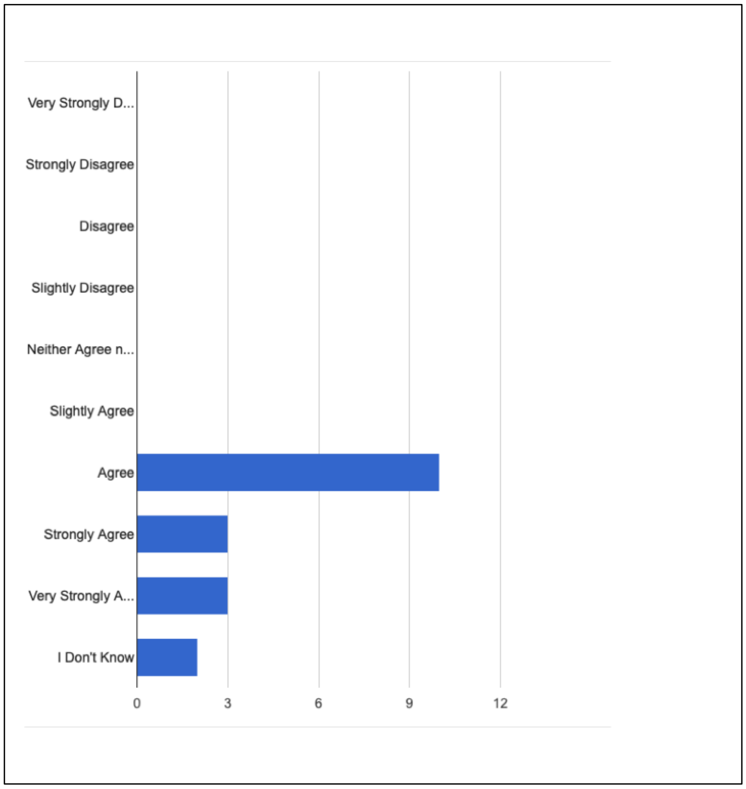

61. Having access to staff and colleagues trained in the application of supportive end of life care helps the LTC physician support the patient and family.

Very Strongly Disagree (0, 0.0%),

Strongly Disagree (0, 0.0%),

Disagree (0, 0.0%),

Slightly Disagree (0, 0.0%),

Neither Agree nor Disagree (0, 0.0%),

Slightly Agree (0, 0.0%),

Agree (9, 50.0%),

Strongly Agree (4, 22.2%),

Very Strongly Agree

(5, 27.8%),

I Don't Know (0, 0.0%)

Median Score 7.5

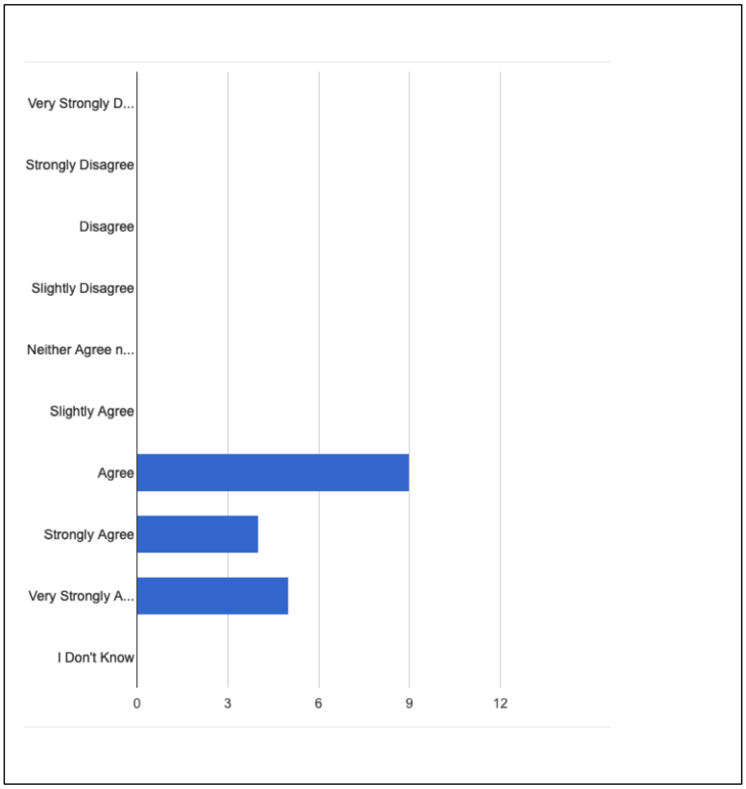

62. Nurse practitioners can help relieve the supportive end of life care burden in LTC.

Very Strongly Disagree (0, 0.0%),

Strongly Disagree (0, 0.0%),

Disagree (1, 5.6%),

Slightly Disagree (0, 0.0%),

Neither Agree nor Disagree (2, 11.1%),

Slightly Agree (1, 5.6%),

Agree (5, 27.8%),

Strongly Agree (3, 16.7%),

Very Strongly Agree

(5, 27.8%),

I Don't Know (1, 5.6%)

Median Score 7

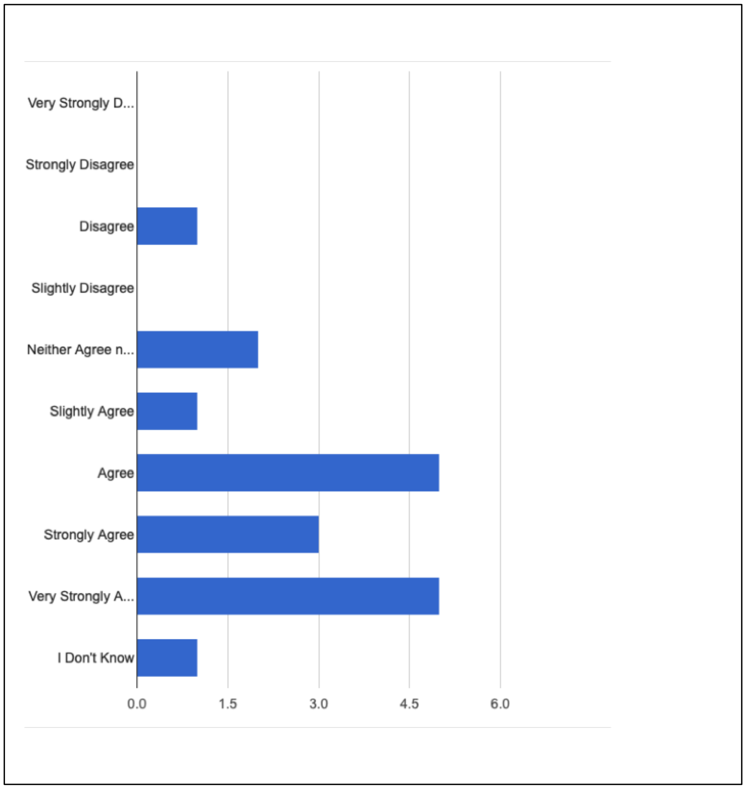

63. Changes in funding models for primary care physicians to work in LTC can improve physician involvement in LTC.

Very Strongly Disagree (0, 0.0%),

Strongly Disagree (0, 0.0%),

Disagree (2, 11.1%),

Slightly Disagree (0, 0.0%),

Neither Agree nor Disagree (1, 5.6%),

Slightly Agree (0, 0.0%),

Agree (5, 27.8%),

Strongly Agree (2, 11.1%),

Very Strongly Agree

(4, 22.2%),

I Don't Know (4, 22.2%)

Median Score 7

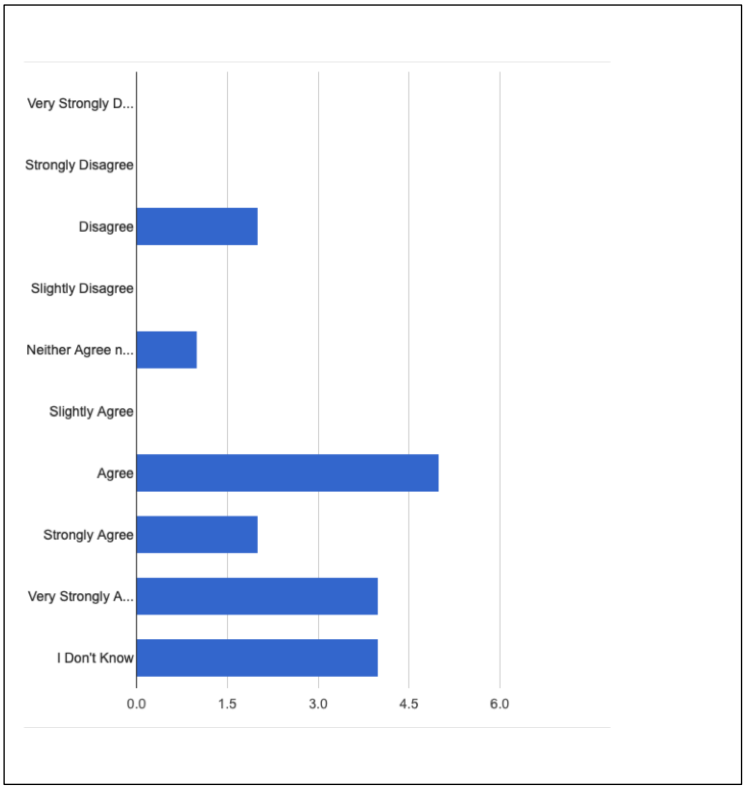

64. Public knowledge and negative perceptions about the quality of care provided within LTC influences a person's decision to attain a higher level of care at end of life (e.g. transfer to hospital).

Very Strongly Disagree (0, 0.0%),

Strongly Disagree (0, 0.0%),

Disagree (0, 0.0%),

Slightly Disagree (0, 0.0%),

Neither Agree nor Disagree (2, 11.1%),

Slightly Agree (0, 0.0%),

Agree (10, 55.6%),

Strongly Agree (1, 5.6%),

Very Strongly Agree

(3, 16.7%),

I Don't Know (2, 11.1%)

Median Score 7

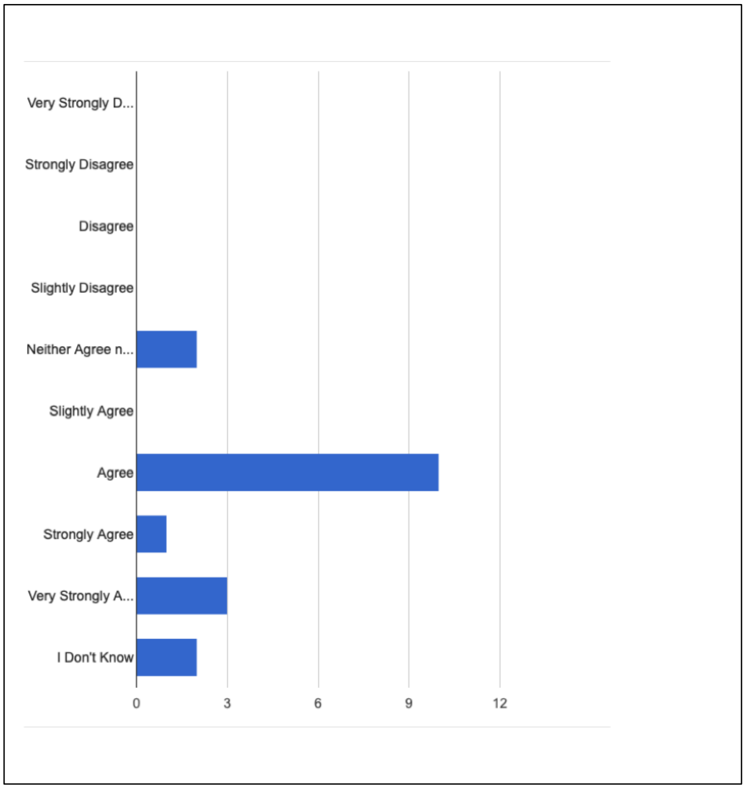

65. Highlighting positive stories about supportive end of life care provided within LTC could help change public perceptions about the quality of care provided within LTC.

Very Strongly Disagree (0, 0.0%),
Strongly Disagree (0, 0.0%),

Disagree (0, 0.0%),

Slightly Disagree (1, 5.6%),

Neither Agree nor Disagree (0, 0.0%),

Slightly Agree (0, 0.0%),

Agree (7, 38.9%),

Strongly Agree (5, 27.8%),

Very Strongly Agree

(5, 27.8%),

I Don't Know (0, 0.0%)

Median Score 8

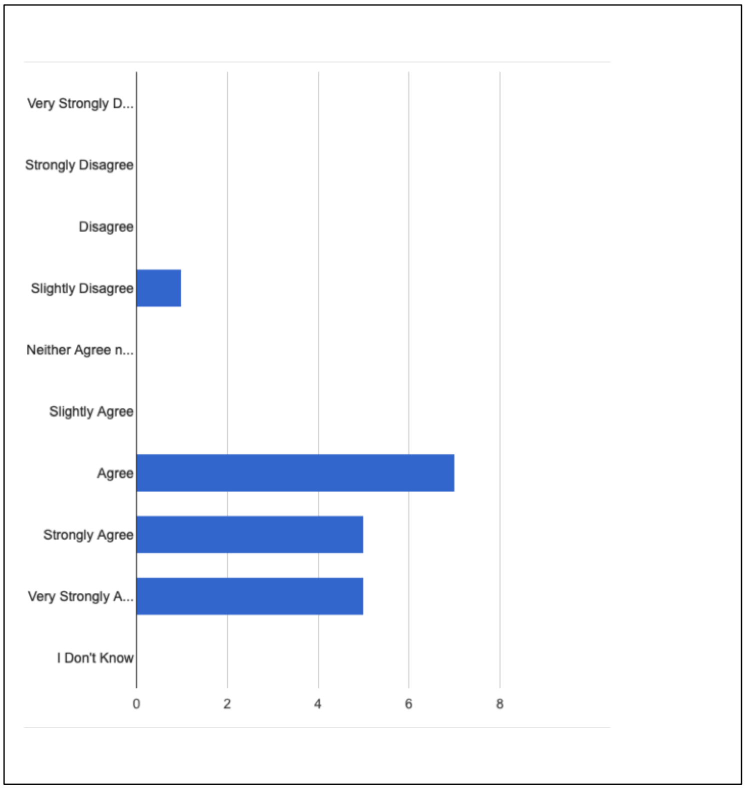

66. Senior management within LTC facilities can dictate how supportive end of life care is prioritized within their facilities through policy and procedures, as well as by influencing culture.

Very Strongly Disagree (0, 0.0%),

Strongly Disagree (0, 0.0%),

Disagree (0, 0.0%),

Slightly Disagree (0, 0.0%),

Neither Agree nor Disagree (0, 0.0%),

Slightly Agree (0, 0.0%),

Agree (7, 38.9%),

Strongly Agree (5, 27.8%),

Very Strongly Agree

(6, 33.3%),

I Don't Know (0, 0.0%)

Median Score 8

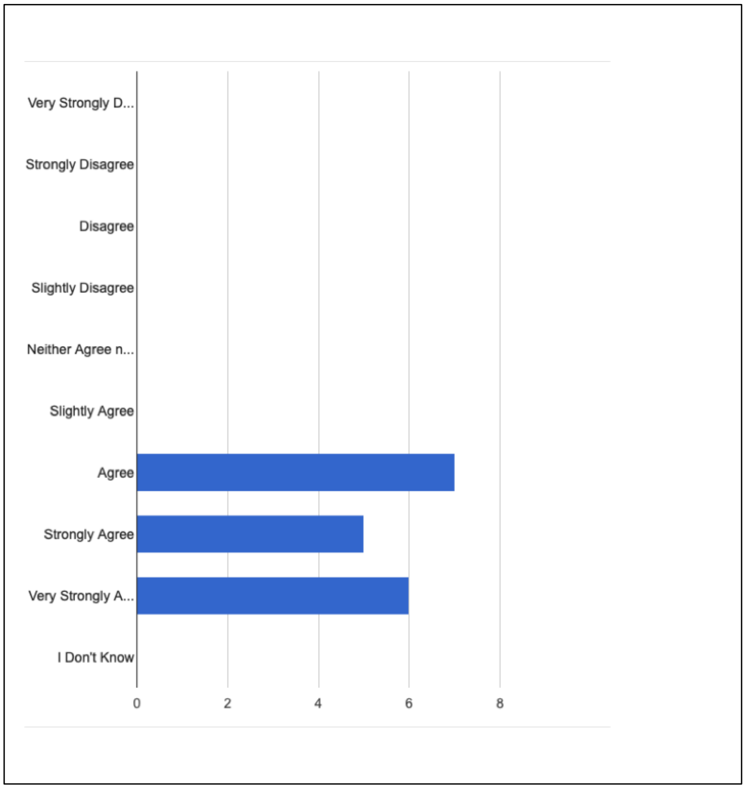

Topics Related to Families (11 Statements)

68. It is important to involve families in conversations about care.

Very Strongly Disagree (0, 0.0%),

Strongly Disagree (0, 0.0%),

Disagree (0, 0.0%),

Slightly Disagree (0, 0.0%),

Neither Agree nor Disagree (0, 0.0%),

Slightly Agree (0, 0.0%),

Agree (1, 5.6%),

Strongly Agree (6, 33.3%),

Very Strongly Agree

(11, 61.1%),

I Don't Know (0, 0.0%)

Median Score 9

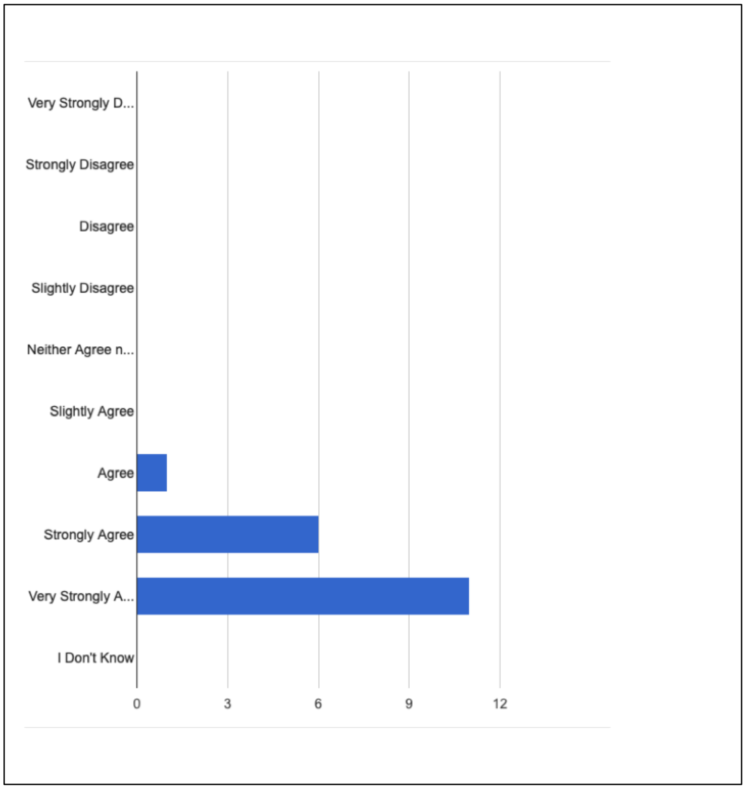

69. Family misperceptions of outcomes can make it challenging to meet their expectations of supportive end of life care treatments.

Very Strongly Disagree (0, 0.0%),

Strongly Disagree (0, 0.0%),

Disagree (0, 0.0%),

Slightly Disagree (0, 0.0%),

Neither Agree nor Disagree (0, 0.0%),

Slightly Agree (0, 0.0%),

Agree (4, 22.2%),

Strongly Agree (9, 50.0%),

Very Strongly Agree

(5, 27.8%),

I Don't Know (0, 0.0%)

Median Score 8

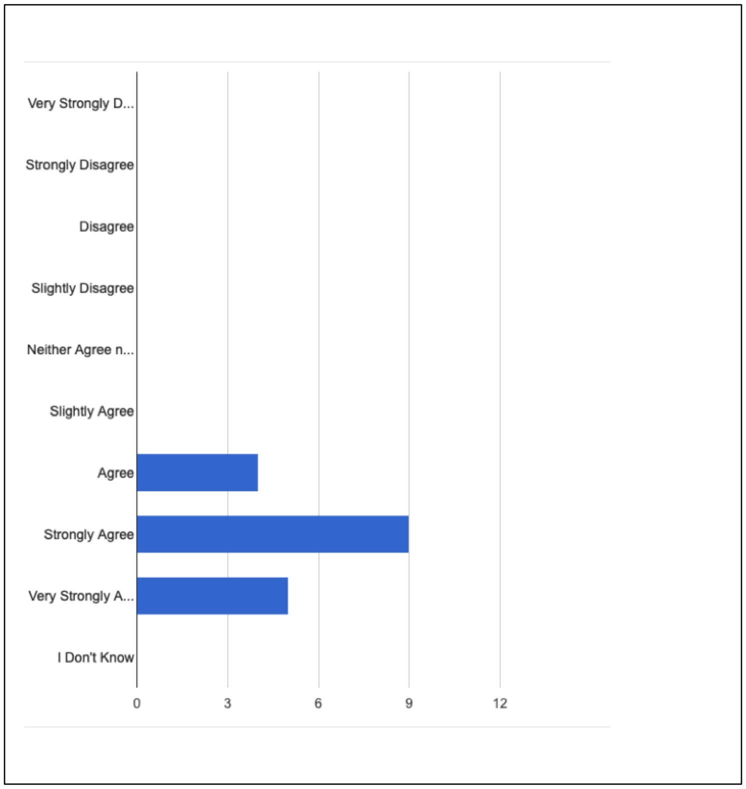

70. Family misunderstandings of supportive care in LTC causes them anxiety, worry, andconfusion.

Very Strongly Disagree (0, 0.0%),

Strongly Disagree (0, 0.0%),

Disagree (0, 0.0%),

Slightly Disagree (0, 0.0%),

Neither Agree nor Disagree (0, 0.0%),

Slightly Agree (2, 11.1%),

Agree (5, 27.8%),

Strongly Agree (6, 33.3%),

Very Strongly

Agree (5, 27.8%),

I Don't Know (0, 0.0%)

Median Score 8

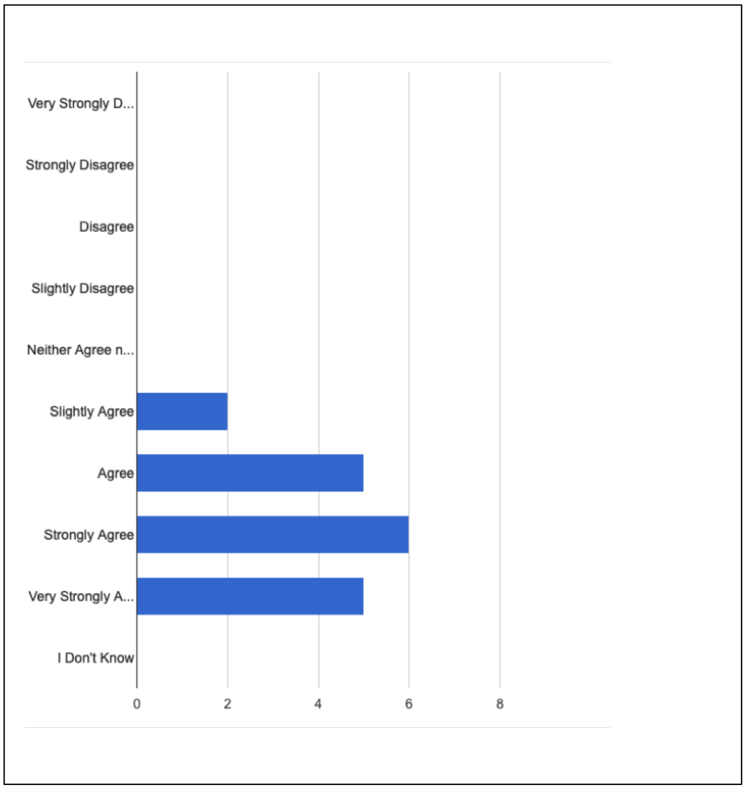

71. Physicians can help families better understand supportive care in LTC by encouraging them to consult credible on-line and written resources and support groups for more information.

Very Strongly Disagree (0, 0.0%),

Strongly Disagree (0, 0.0%),

Disagree (0, 0.0%),

Slightly Disagree (0, 0.0%),

Neither Agree nor Disagree (1, 5.6%),

Slightly Agree (3, 16.7%),

Agree (6, 33.3%),

Strongly Agree (3, 16.7%),

Very Strongly

Agree (4, 22.2%),

I Don't Know (1, 5.6%)

Median Score 7

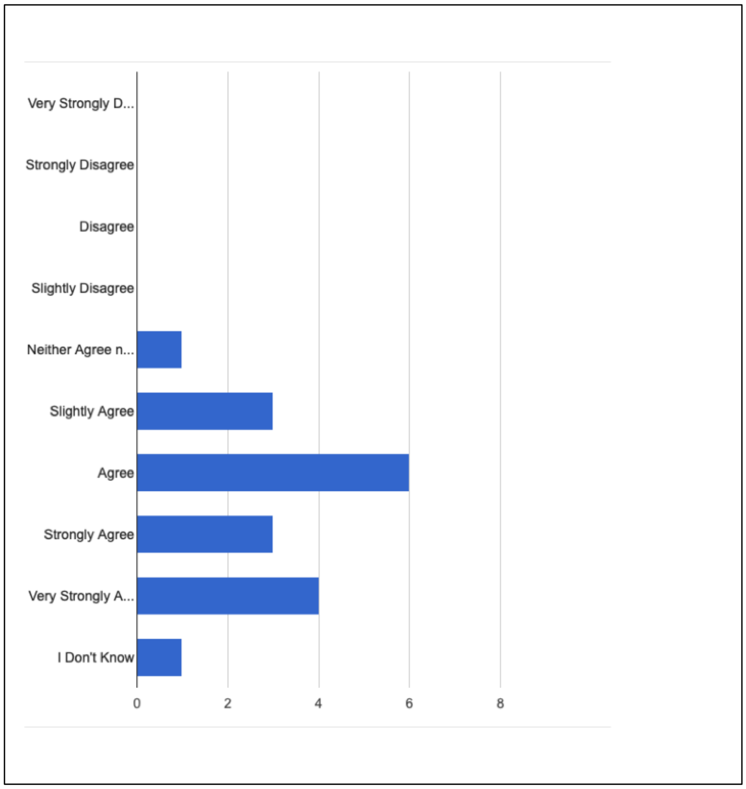

72. Involving palliative care consultants can help improve family members understanding of supportive care, including helping to manage family expectations.

Very Strongly Disagree (0, 0.0%),

Strongly Disagree (0, 0.0%),

Disagree (0, 0.0%),

Slightly Disagree (0, 0.0%),

Neither Agree nor Disagree (0, 0.0%),

Slightly Agree (1, 5.6%),

Agree (6, 33.3%),

Strongly Agree (4, 22.2%),

Very Strongly Agree

(6, 33.3%),

I Don't Know (1, 5.6%)

Median Score 8

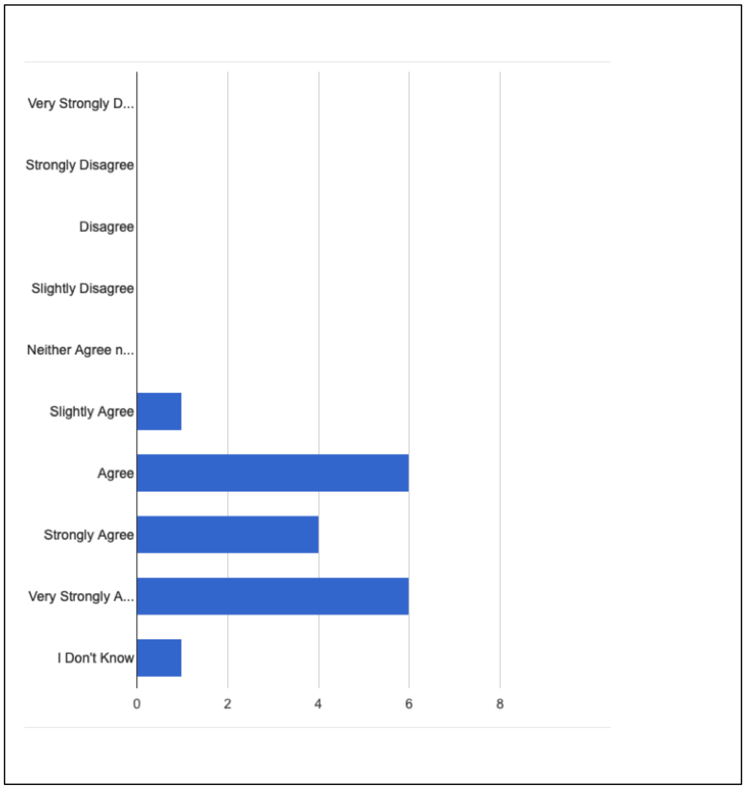

73. Family values are factors that determine the level of care provided.

Very Strongly Disagree (0, 0.0%),

Strongly Disagree (0, 0.0%),

Disagree (0, 0.0%),

Slightly Disagree (0, 0.0%),

Neither Agree nor Disagree (0, 0.0%),

Slightly Agree (1, 5.6%),

Agree (7, 38.9%),

Strongly Agree (3, 16.7%),

Very Strongly Agree

(6, 33.3%),

I Don't Know (1, 5.6%)

Median Score 8

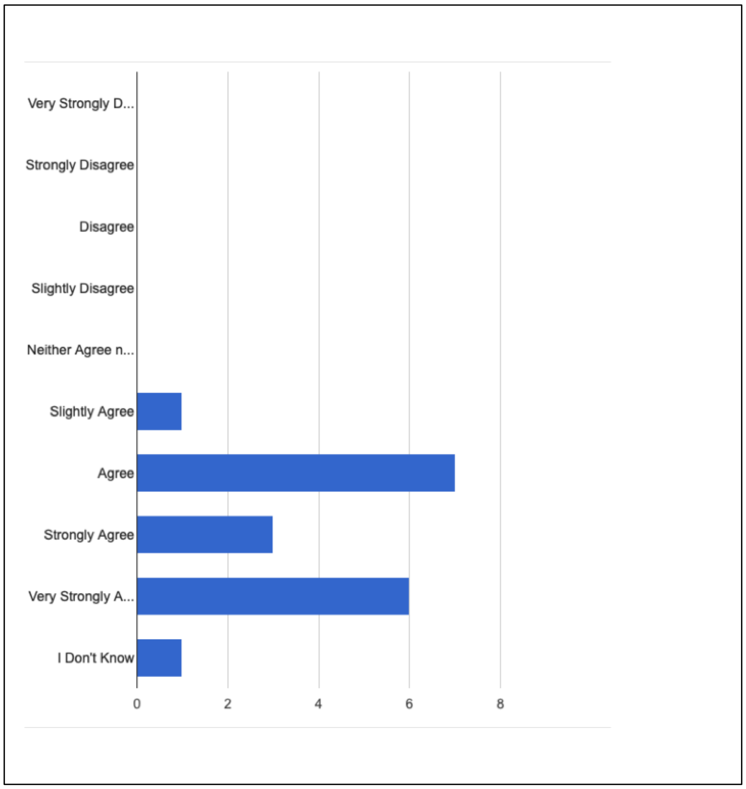

74. Goals of Care assessment tools help families understand what supportive end of life care means in LTC.

Very Strongly Disagree (0, 0.0%),

Strongly Disagree (0, 0.0%),

Disagree (0, 0.0%),

Slightly Disagree (1, 5.6%),

Neither Agree nor Disagree (0, 0.0%),

Slightly Agree (1, 5.6%),

Agree (7, 38.9%),

Strongly Agree (5, 27.8%),

Very Strongly Agree

(4, 22.2%),

I Don't Know (0, 0.0%)

Median Score 7.5

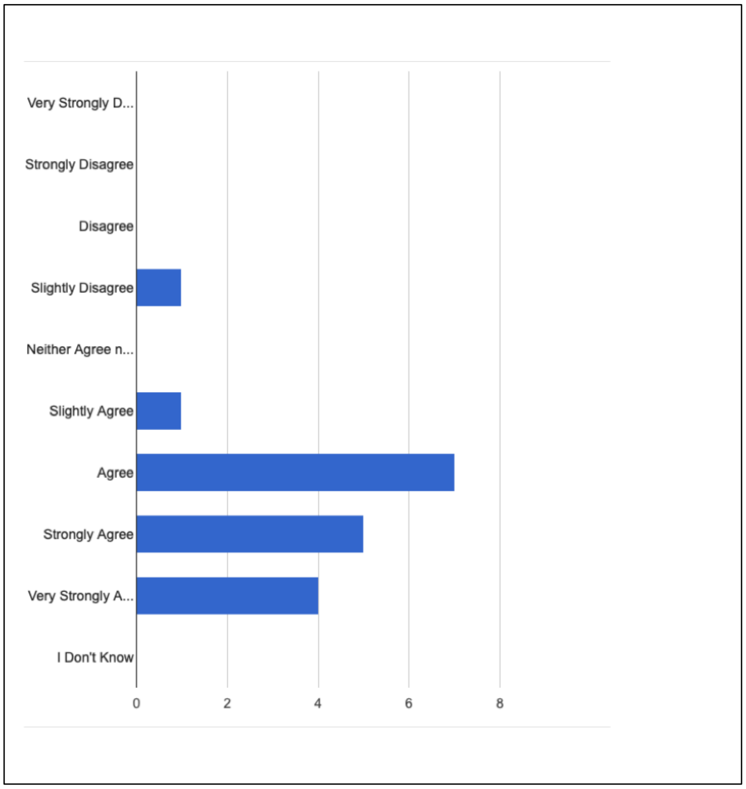

75. Goals of Care assessment tools help physicians manage care expectations and create care plans with the family.

Very Strongly Disagree (0, 0.0%),

Strongly Disagree (0, 0.0%),

Disagree (0, 0.0%),

Slightly Disagree (0, 0.0%),

Neither Agree nor Disagree (0, 0.0%),

Slightly Agree (0, 0.0%),

Agree (6, 33.3%),

Strongly Agree (7, 38.9%),

Very Strongly Agree

(5, 27.8%),

I Don't Know (0, 0.0%)

Median Score 8

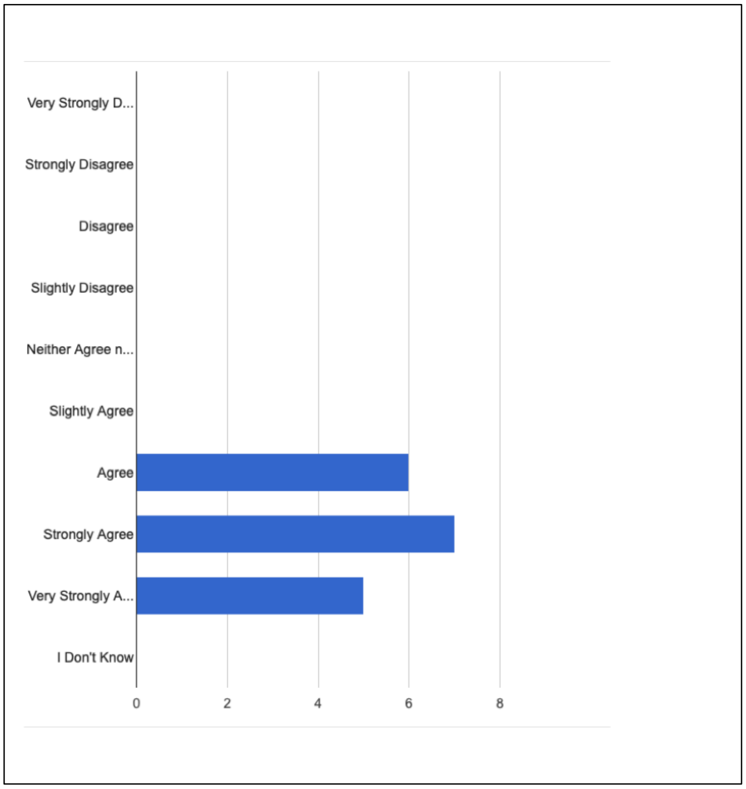

76. Families can be helpful when determining goals of care given their intimate knowledge of the patient's goals and values.

Very Strongly Disagree (0, 0.0%),

Strongly Disagree (0, 0.0%),

Disagree (0, 0.0%),

Slightly Disagree (0, 0.0%),

Neither Agree nor Disagree (0, 0.0%),

Slightly Agree (0, 0.0%),

Agree (5, 27.8%),

Strongly Agree (6, 33.3%),

Very Strongly Agree

(7, 38.9%),

I Don't Know (0, 0.0%)

Median Score 8

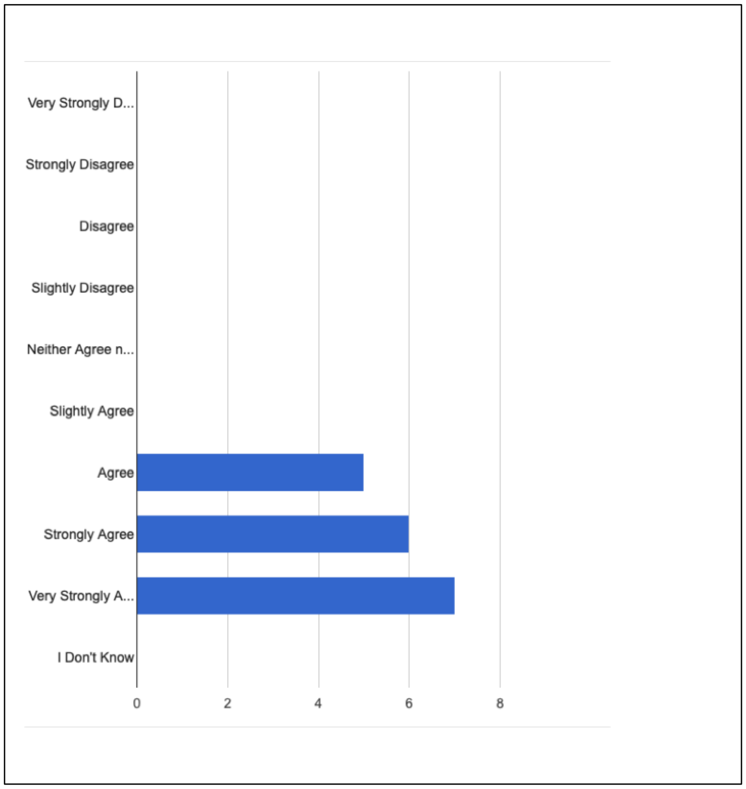

77. It is important to support patients and families by making it possible for them to access multidisciplinary care and support services.

Very Strongly Disagree (0, 0.0%),

Strongly Disagree (0, 0.0%),

Disagree (0, 0.0%),

Slightly Disagree (0, 0.0%),

Neither Agree nor Disagree (0, 0.0%),

Slightly Agree (1, 5.6%),

Agree (3, 16.7%),

Strongly Agree (6, 33.3%),

Very Strongly Agree

(8, 44.4%),

I Don't Know (0, 0.0%)

Median Score 8

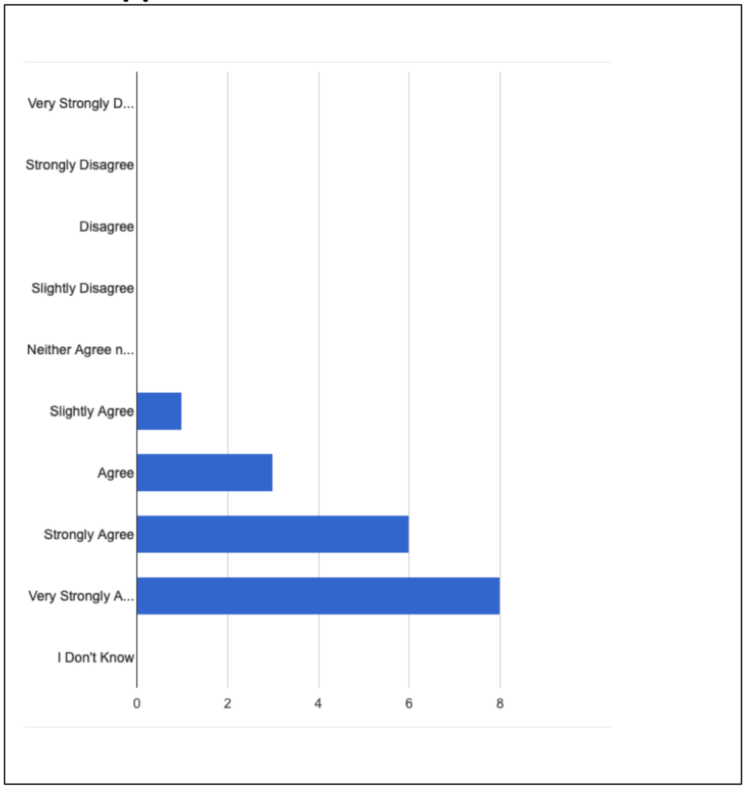

78. Having someone in the facility with some degree of authority helps families trust the decision to transition the patient from chronic supportive medical care to supportive palliative end of life care.

Very Strongly Disagree (0, 0.0%),

Strongly Disagree (0, 0.0%),

Disagree (1, 5.6%),

Slightly Disagree (0, 0.0%),

Neither Agree nor Disagree (3, 16.7%),

Slightly Agree (2, 11.1%),

Agree (2, 11.1%),

Strongly Agree (2, 11.1%),

Very Strongly

Agree (6, 33.3%),

I Don't Know (2, 11.1%)

Median Score 7.5

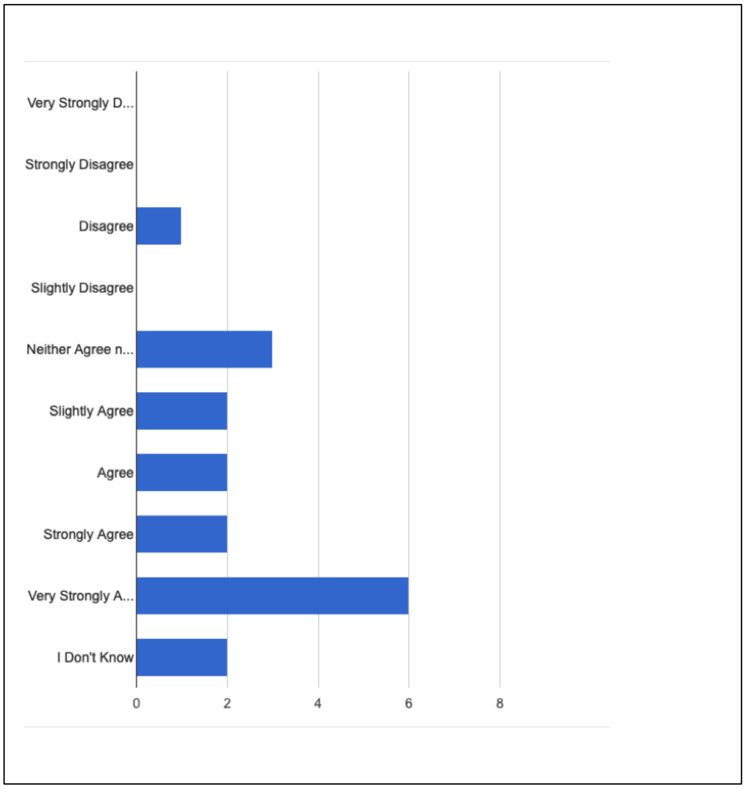

Delphi Two Questionnaire Median Results

1. What is your role in long-term care (LTC)?

Family Physician (3, 27.3%),

Specialist Physician (1, 9.1%),

Nurse (Includes RN, LPN) (1,9.1%),

Health Care Aide (0,0.0%),

Healthcare provider (other than physician, RN, LPN, Health Care Aide) (1,9.1%),

LTC Manager/Administrator (1,9.1%),

Resident Living in LTC (0,0%),

Family Member of Resident Living in LTC (2, 18.2%),

Researcher/Knowledge Translation Expert (2,18.2%)

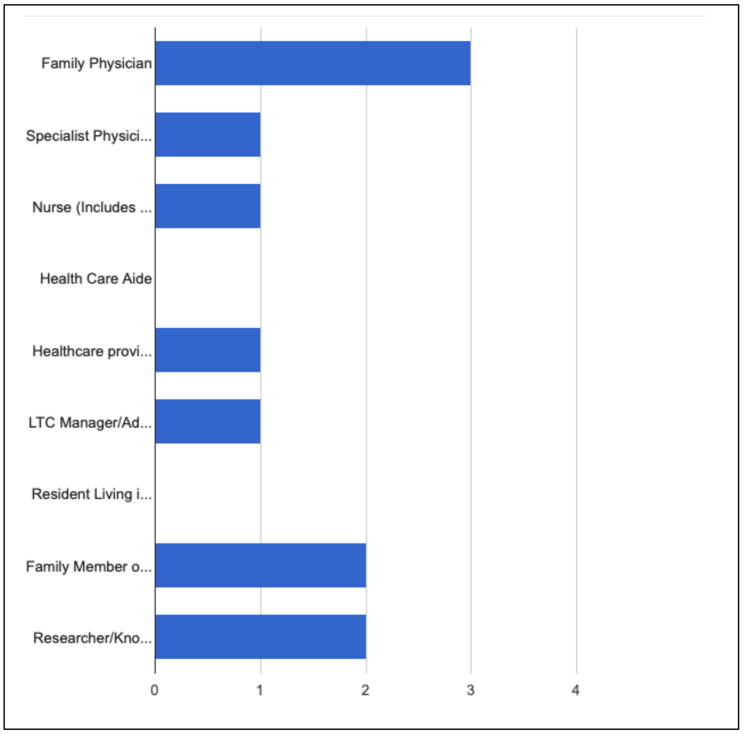

2. What is your age?

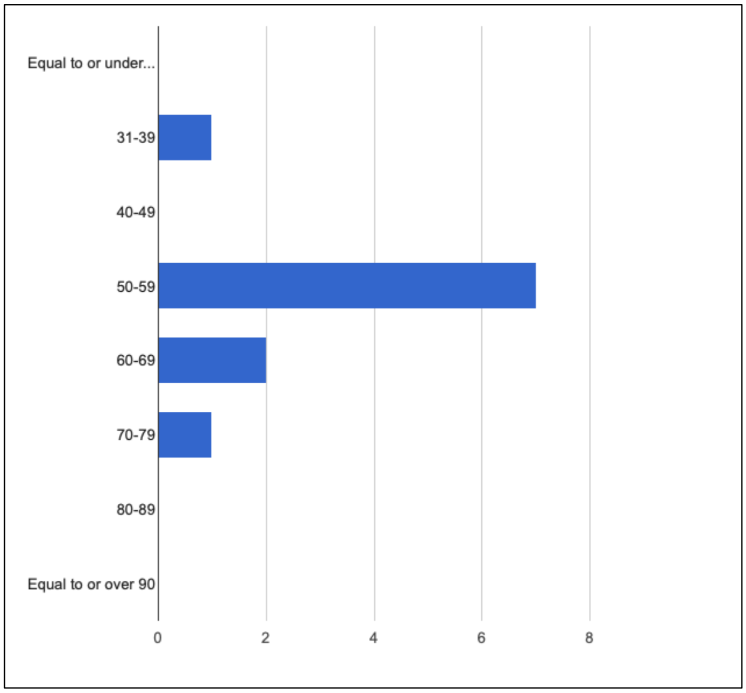

Equal to or under 30 (0,0%),

31-39 (1,9.1%),

40-49 (0,0%),

50-59 (7,63.3%),

60-69 (2,18.2%),

70-79 (1,9.1%),

80-89 (0, 0.0%),

Equal to or over 90

(0, 0.0%)

3. In what province do you live?

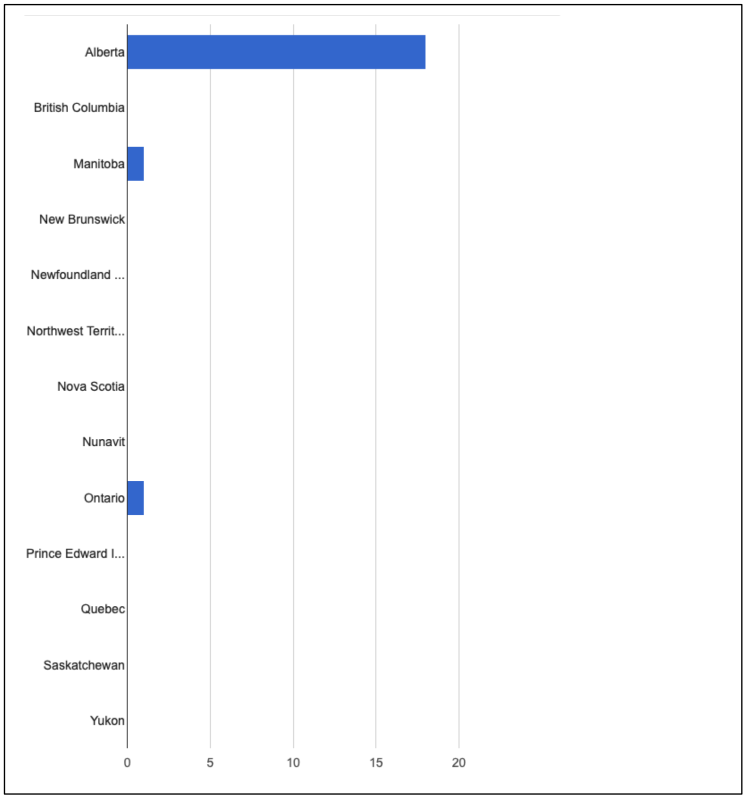

Alberta (9,81.8%),

British Columbia (0, 0.0%),

Manitoba (1, 9.1%),

New Brunswick (0, 0.0%),

Newfoundland and Labrador (0, 0.0%),

Northwest Territories (0, 0.0%),

Nova Scotia (0, 0.0%),

Nunavit (0, 0.0%),

Ontario (1, 9.1%),

Prince Edward

Island (0, 0.0%),

Quebec (0, 0.0%),

Saskatchewan (0, 0.0%),

Yukon (0, 0.0%)

4. **Based on population size, is your primary place of residence/work located in an urban or rural setting?**

Urban large size population

(100,00 and over) (10,90.9%),

Urban medium population

(between 30,000 and 99,999) (1,9.1%),

Urban small population

(between 1000 and 29,999) (2, 10.0%),

Rural - territory located outside

any size urban area

(0, 0.0%)

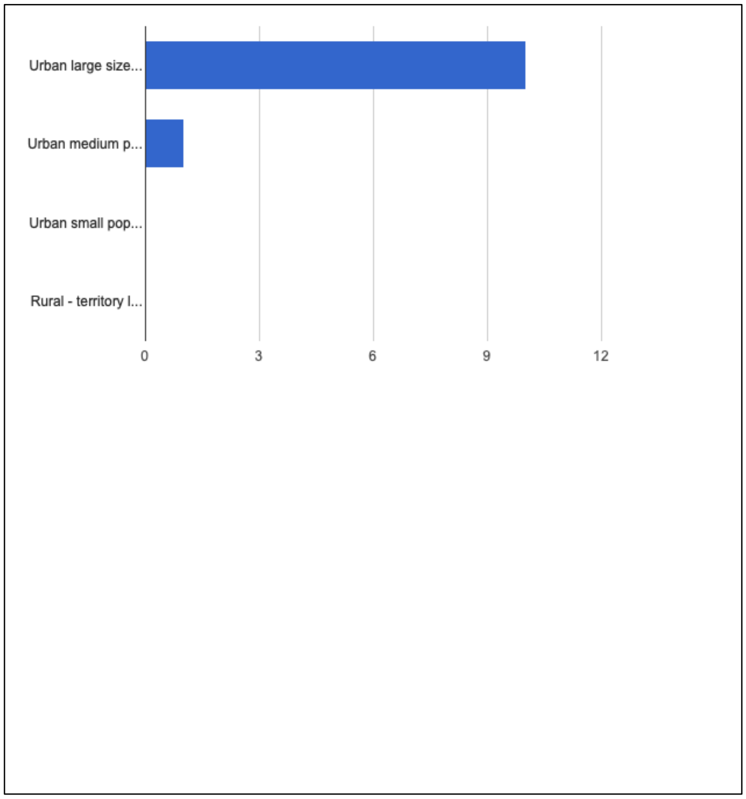

5. **Staff lack the time needed to use pain assessment tools.**

Very Strongly Disagree (1,9.1%),

Strongly Disagree (0, 0.0%),

Disagree (4,36.4%),

Slightly Disagree (0, 0.0%),

Neither Agree nor Disagree (0, 0.0%),

Slightly Agree (2,18.2%),

Agree (2, 18.2%),

Strongly Agree (1,9.1%),

Very Strongly

Agree (0,0.0%),

I Don't Know (1,9.1%)

Median Score 5.18

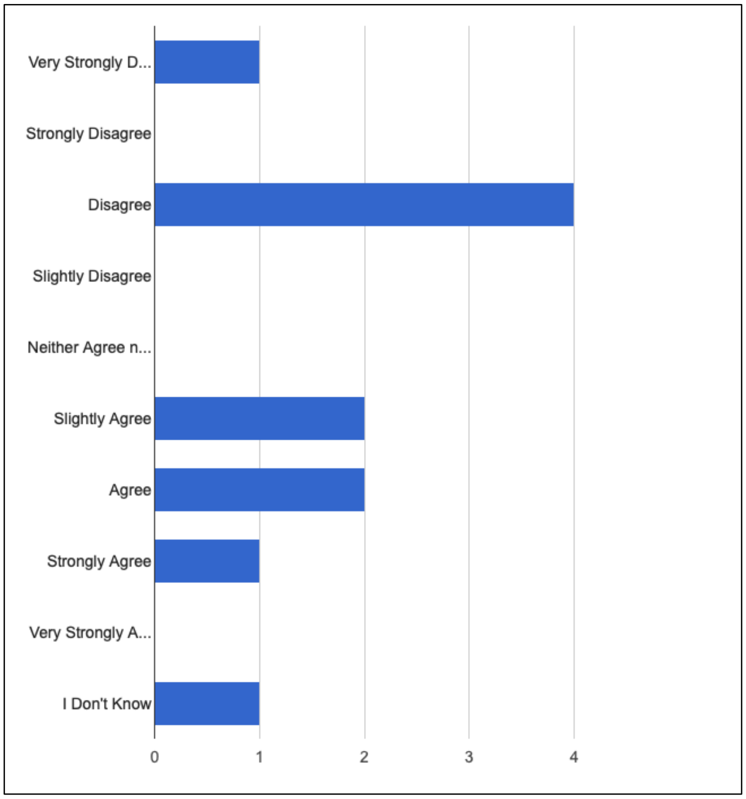

6. LTC staff are uncomfortable with providing supportive end of life care.

Very Strongly Disagree (0,0%),

Strongly Disagree (1, 9.1%),

Disagree (3,27.3%),

Slightly Disagree (0,0.0%),

Neither Agree nor Disagree (2, 18.2%),

Slightly Agree (1,9.1%),

Agree (2,18.2%),

Strongly Agree (2,18.2%),

Very Strongly Agree

(0, 0.0%),

I Don't Know (0,0.0%)

Median Score 5.18

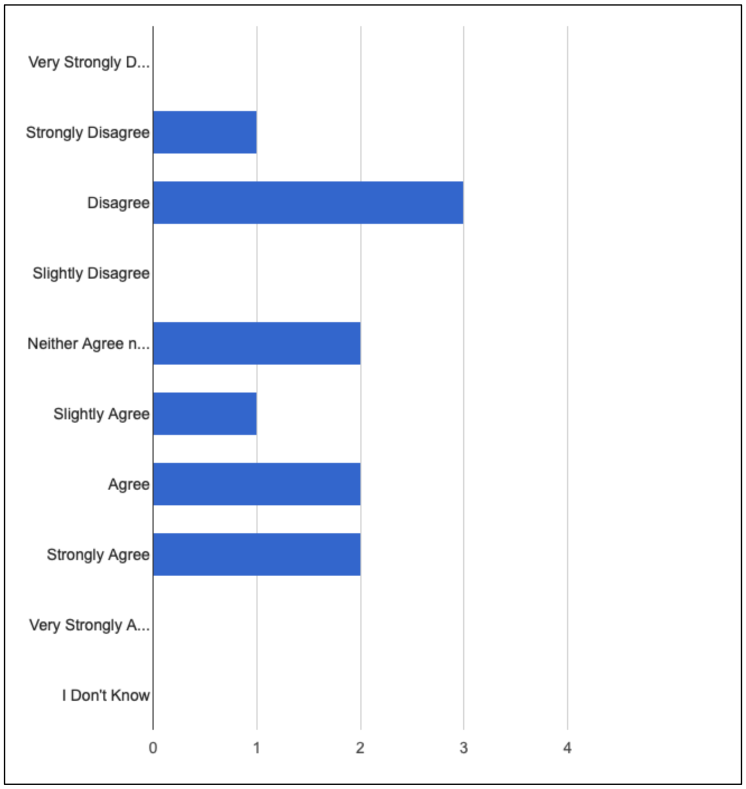

7. LTC staff are unknowledgeable and/or inexperienced in supportive end of life care.

Very Strongly Disagree (0,0.0%),

Strongly Disagree (2,18.2%),

Disagree (4, 36.4%),

Slightly Disagree (1,9.1%),

Neither Agree nor Disagree (1, 9.1%),

Slightly Agree (1,9.1%),

Agree (1,9.1%),

Strongly Agree (1, 9.1%),

Very Strongly Agree

(0,0.0%),

I Don't Know (0,0.0%)

Median Score 4.18

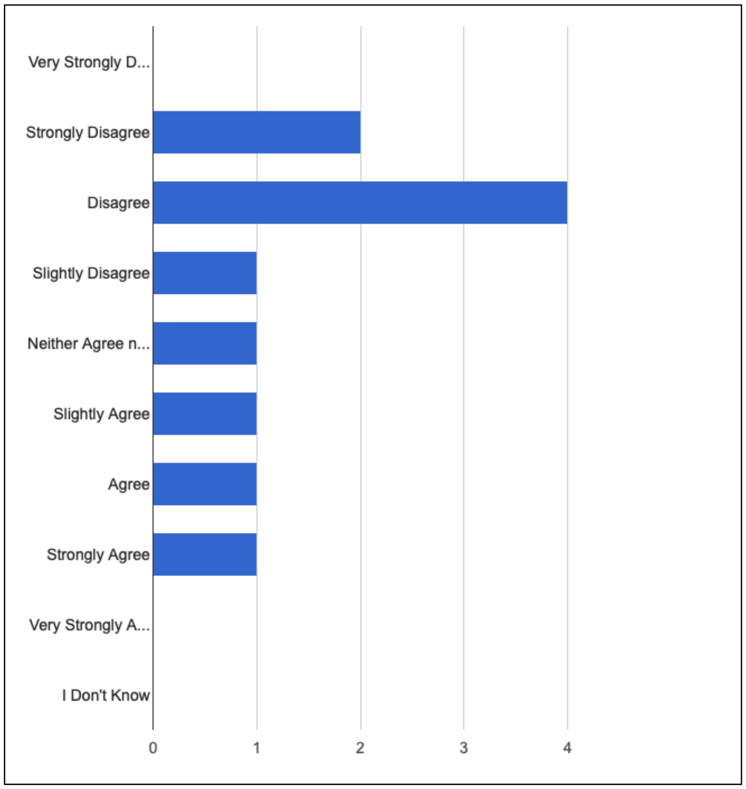

8. The best education for staff initially resistant to providing supportive end of life care in LTC is the practical knowledge they gain from managing symptoms in difficult situations.

Very Strongly Disagree (1,9.1%),

Strongly Disagree (0, 0.0%),

Disagree (0,0.0%),

Slightly Disagree (0, 0.0%),

Neither Agree nor Disagree (0,0.0%),

Slightly Agree (4, 36.4%),

Agree (3, 27.3%),

Strongly Agree (1,9.1%),

Very Strongly

Agree (1,9.1%),

I Don't Know (1,9.1%)

Median Score 6.63

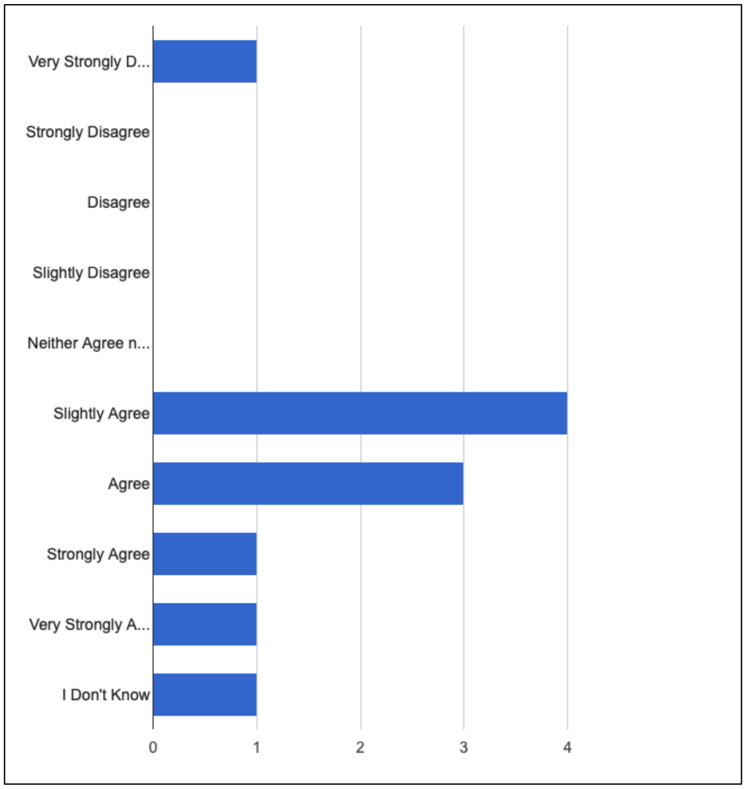

Supplement: Supplementary file 3 — Additional file 3. [file 12877_2021_2271_MOESM3_ESM.pdf]
